# Charting the human-specific properties of gene expression networks in the infant prefrontal cortex

**DOI:** 10.1126/sciadv.aea3316

**Published:** 2026-06-03

**Authors:** Jonathan Klavert, Djawad Radjabzadeh, Erlantz Gonzalez Sanchez, Bas Castelijns, Ilia S. Timpanaro, Joachim Boers, Federica Fabro, Gerjanne Vroeg in de wei, Eric Bindels, Ivanela Kondova, Joost Gribnau, Menno P. Creyghton

**Affiliations:** ^1^ Department of Developmental Biology, Erasmus University Medical Center, Wytemaweg 80, 3015 CN Rotterdam, Netherlands.; ^2^ Hubrecht Institute-KNAW & University Medical Center Utrecht, Uppsalalaan 8, 3584 CT Utrecht, Netherlands.; ^3^ Rudolf Magnus Brain Center, University Medical Center Utrecht, Heidelberglaan 100, 3584 XC Utrecht, Netherlands.; ^4^ Department of Hematology, Erasmus University Medical Center, Wytemaweg 80, 3015 CN Rotterdam, Netherlands.; ^5^ Biomedical Primate Research Center, Lange Kleiweg 161, 2288 GJ Rijswijk, Netherlands.

## Abstract

Human infancy is characterized by protracted brain development coinciding with sensitive periods of extensive synaptic remodeling. Whether this is supported by human infant–specific transcriptional programs is unknown as comparative material in closely related primate species was unavailable. Here, we analyze rare newborn chimpanzee and age-matched human and rhesus macaque brain samples using single-cell transcriptomics and epigenomics. We identify a human infant–specific transcriptional program in immature oligodendrocytes that is overrepresented in autism risk genes and patient gene expression changes. Furthermore, a human infant–specific transcriptional program in the neural lineage is overrepresented in Parkinson’s disease risk genes and patient gene expression changes. Both of these programs are part of a core transcriptional network that contains human-specific sequence changes in regulatory DNA and lacks cell lineage specificity. Our study provides insights into the stage-specific properties of human evolution during early infancy and sheds light on the human-specific propensities to neural disease.

## Teaser

Human infant–specific processes in the brain connect human evolution to neuropsychiatric and neurodegenerative disease.

## INTRODUCTION

Compared to the brains of nonhuman primates, the human brain has an increased volume, enhanced projections between cortical areas including the frontal cortex and regions involved in language processing, and an increased complexity and size of upper-layer neurons (*1*). These phenotypes are accompanied by an increased production of neural stem cells during early neurogenesis, accelerated volumetric growth during gliogenesis (*2*), as well as protracted myelination by oligodendrocytes (OLs) in infancy and childhood (*3*) and the emergence of human-specific transcriptional programs (*4*–*6*).

Human-specific (from here on defined as altered in human samples compared to the other nonhuman primates that were investigated) transcriptional and epigenomic changes have been well characterized in adults (*7*–*11*). However, studies of the equivalent changes during relevant developmental stages have been limited to either cell culture proxies (*12*–*14*) or comparisons to lower primate species (*15*, *16*). Most notably, there is little data for late fetal and early postnatal stages during which the brain undergoes gliogenesis, synaptogenesis, and myelination. This is primarily because of limited tissue availability in great apes and the difficulty of reaching these stages in culture (*17*, *18*). Given that key gene expression programs that dictate developmental processes are typically temporally restricted (*19*), these limitations have left a substantial gap in our understanding of human brain evolution during sensitive stages of brain development when language acquisition and socialization occur (*20*, *21*).

Here, we provide single-cell transcriptomic and epigenomic analysis of human, rhesus macaque, and chimpanzee brain tissue during early postnatal development. We characterize human infant–specific programs and cell states that are not observed in adults and correlate early life processes with Parkinson’s disease (PD) susceptibility in aged neurons. Furthermore, we demonstrate that these human infant–specific programs are enriched for human-specific insertions (*hINSs*) and human-specific deletions (*hDELs*) in regulatory DNA and that the networks that these elements are part of lack the typical lineage specificity observed in newly evolved elements but manifest in a cell type–specific manner. Our data provide important insights into both the human-specific properties of postnatal development and reveal clues as to the human-specific propensities for neural disease.

## RESULTS

### Single-nucleus RNA sequencing of the postnatal chimpanzee, human, and macaque prefrontal cortex

**Fig. 1.**
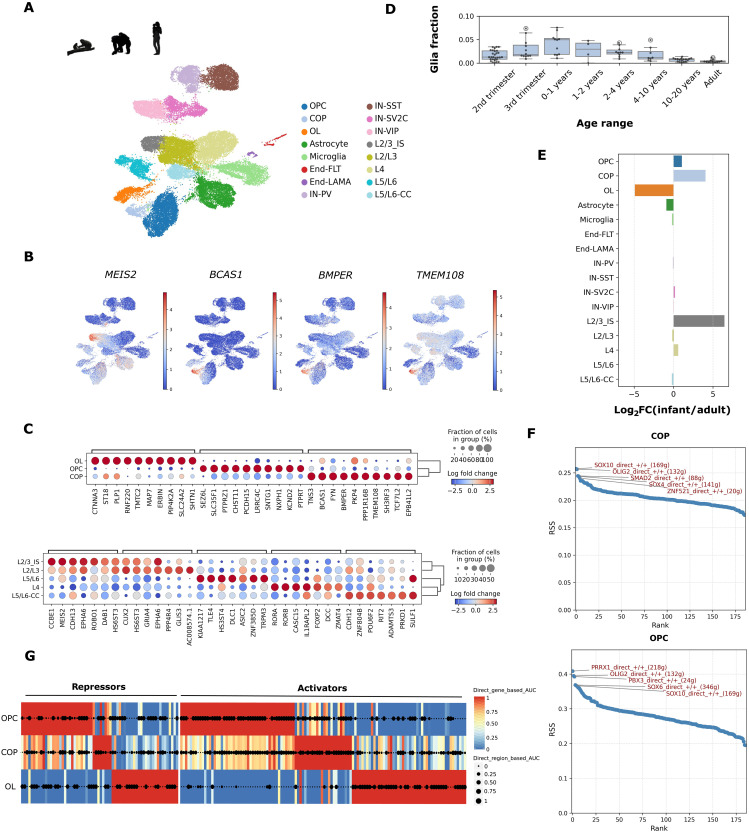
Generation of single-cell transcriptomics for the adult and infant PFC across primates. (**A**) Uniform manifold approximation and projection (UMAP) showing the Leiden clustering of snRNA-seq data from PFC tissue (*n* = ~38,000 nuclei post–quality control, 11 individuals) covering infant and adult human, chimpanzee, and rhesus macaque DLPFC samples following integration. Colors indicate clusters, and cell type labels were assigned using known cell type–specific markers (see fig. S2). (**B**) Marker gene expression for representative cell types. Indicated in this panel are markers for infant-specific L2/3 neurons (L2/3_IS; *MEIS2*) and COPs (*BCAS1* and *BMPER*). Coloring indicates log_2_(reads per 10,000 unique transcripts). (**C**) Dot plot of top marker genes (columns) per cell type (rows). Coloring indicates log_2_ fold change (FC) in expression. (**D**) Reanalysis of human brain atlas data for COP prevalence throughout human development (*n* = 106 individuals; ~170,000 nuclei) (*24*). Proportion of COPs is calculated per individual and compared between age groups. (**E**) Quantification of cell types enriched in the infant PFC over adult samples complementary to (D). Bars indicate log_2_FC comparing infants versus adults. Positive values indicate the proportional enrichment in infant or adult samples. (**F**) Top five regulons for COPs (top) and OPCs (bottom) as measured by regulon specificity score (RSS). (**G**) Single-cell regulatory network inference and clustering (SCENIC+) data analysis showing a regulon heatmap of OL lineage cells per cell type (columns). Rows indicate a regulon (TF + target genes + ATAC regions). Colors indicate TF-to-gene area under the curve (AUC) values, whereas dot sizes indicate region-to-gene AUC values. Activator and repressor regulons are separated as indicated for the three cell types with complementary data in figs. S6 and S7.

To identify the characteristics of early postnatal development of the human prefrontal cortex (PFC) in humans, we used single-cell RNA sequencing (RNA-seq) on dorsolateral prefrontal cortex (DLPFC) tissue from infant human (*n* = 3, 2 to 4 months), chimpanzee (*n* = 2, 0 to 5 months), and rhesus macaque (*n* = 2, 0 months) (Fig. 1A and table S1). All of these fall within stage 8 (0 to 6 months) of early primate development as described previously (*22*). As the samples included two rare infant chimpanzee specimens that were both male, the other samples were sex matched accordingly, and several adult samples were also added (*n* = 2 humans and 3 chimpanzees; table S1). None of the specimens were diagnosed with developmental defects or disease. We performed single-nucleus RNA sequencing (snRNA-seq) and generated gene expression profiles for 59,121 nuclei. Following stringent quality control and removal of nuclei with high amounts of ambient RNA (*23*), we proceeded with a total of 37,994 nuclei. Samples from different species were pooled and processed simultaneously to reduce experimental variation, as shown previously (*12*), with species identity being recovered during data analysis by species-specific genome alignment (fig. S1A; Materials and Methods). Median genes (*n* = 2178) and unique transcripts (*n* = 4192) per nuclei were well within quality parameters (*24*) and samples clustered according to cell type rather than batch or other quality parameters (fig. S1, B to D). Following batch correction using BBKNN-mediated data integration (*25*), unbiased clustering followed by marker gene analysis revealed species conserved cell types including four major classes of inhibitory neurons and five subclasses of excitatory projection neurons (fig. S2, A and B). Furthermore, eight nonneuronal cell clusters were subcategorized into three types of OLs, including oligodendrocyte progenitor cells (OPCs), mature OLs, and premyelinating or committed oligodendrocyte progenitors (COPs), as well as astrocytes, and microglia (Fig. 1, A to C; fig. S2, B and C; and table S1). These identified cell types are largely consistent with other studies (*24*, *26*, *27*).

### Characterization of infant-enriched immature cell types

The overall analysis of cellular content was similar between adults and infants with an expected bias toward OL progenitors over differentiated OLs in infants across species (Fig. 1E and table S2). Notably, we identified two additional cell clusters primarily associated with newborn samples (fig. S2B) that were robust to the clustering method used (fig. S1D). One of those infant-enriched clusters contained *CUX2*-positive L2/3 projection neurons that was only observed in infants and were therefore labeled infant-specific L2/3 (L2/3-IS). This L2/3-IS cell state in projection neurons was found in infants from all three primate species (fig. S2B). L2/3-IS cells were characterized by the expression of the transcription factor (TF) *MEIS2* (Fig. 1B), which is mutated in intellectual disability (*28*) as well as by genes involved in neural differentiation, neural projection, and attractive and repulsive signaling during axon guidance (table S1).

The second infant-enriched cluster contained *BCAS1*-expressing OLs (Fig. 1B), matching previously described rare premyelinating OLs in the mouse brain (*29*, *30*). These cells were more recently described as rare COPs in the adult human brain (*31*). We found that these cells were numerous in infants enabling their robust characterization and comparison across primate species. Unlike OPCs and OLs, infant-enriched COPs were defined by genes connected to the perisynaptic space, glial projection, and neuron projection guidance (table S1). Infant-enriched COPs were also characterized by high expression of *TMEM108* (Fig. 1C), which encodes a transmembrane protein that delays proliferation and myelination of OL lineage cells (*32*). This gene is also associated with several neuropsychiatric disorders as well as disease progression in PD (*33*).

### Temporal dynamics of infant-enriched cell types during human development

To characterize the temporal dynamics of the two infant-enriched cell states during human development, we leveraged single-cell RNA-seq data generated across pre- and postnatal developmental stages through childhood into adulthood in human cortical samples (*24*, *27*, *34*). The composite analysis spanned 171,175 glial nuclei and 374,463 excitatory neurons from 106 human individuals (table S2). BBKNN integration of this composite dataset revealed similar clustering of all major cell types including the two cell states identified above (figs. S3, A to C, and S4, A and B). Infant-enriched COPs showed a gradual increase in utero, peaking during the first year of life (0- to 1-year age group; ~5% of glial nuclei) and gradually decreasing during adolescence to adult levels (>20-year age group; ~0.4% of glial nuclei) (Fig. 1D and table S1). Last, we found that the L2/3-IS cells were distinguishable from other L2/3 neurons only during the first year of life whereas, during the third trimester, they were similar to progenitors (fig. S4, A and B). Thus, L2/3-IS cells, which are shared across primate species, represent a precise temporal cell state during the first year of life that coincides with the presence of high numbers of COPs.

### Primate conserved patterns of intercellular communication in the postnatal DLPFC

To identify possible cellular codependencies involving infant-enriched cell states, we leveraged the LIANA framework to predict intercellular communication. This analysis creates a ranked consensus score between several tools to predict high-confidence ligand-receptor (LR) interactions (*35*). We aggregated cells from the three primate species based on their identity and assessed cell type–specific signaling patterns. Predicted LR interactions showed a large degree of cell type redundancy as only 63 of 32,478 (~0.2%) demonstrated cell type specificity in a source-target relationship (fig. S4D and table S4). We identified L2/3-IS cells as receivers of specific output from glia progenitors in the form of WNT3-FZD3_LRP6 (LR) interactions (specificity rank = 0.031), as well as pan-neural WNT10B output primarily from canonical L2/3 cells (specificity rank = 0.048).

In contrast, we identified COPs as robust senders of several bone morphogenetic protein (BMP) ligands (BMP2, BMP4, and BMP8A), which was not observed from OPCs or mature OLs (fig. S3C). Notably, our data show that, in primates, COPs are characterized by expression of *BMPER* (Fig. 1C and fig. S3, A and B), which functions during development as a context-dependent rheostat for BMP signaling (*36*). These BMP ligands from COPs are likely received by L2/3-IS cells via the ACVR2A_BMPR1B complex, which was the strongest predicted interaction overall (specificity rank = 0.005) (fig. S3C and table S4). Together, our results show specific cross-talk between infant-enriched COPs and L2/3-IS, via BMP signaling, and between L2/3-IS and glial progenitors via Wnt signaling, demonstrating complex intercellular dependencies associated with the presence of these infant-enriched states.

### Multiomic characterization of infant-enriched COPs and L2/3 subtypes

To dissect the transcriptional networks driving the two infant-enriched cell states, we performed multiome analysis combining single-cell RNA-seq and single-cell assay for transposase-accessible chromatin sequencing (scATAC-seq) in the same cells using a chimpanzee and human infant sample (Fig. 1F and figs. S5 and S6). As regulatory DNA is typically different between species (*37*), we used the SCENIC+ framework (*38*) to systematically model the human/chimpanzee conserved relationship between TFs and gene regulatory elements (GREs) and target genes (i.e., a regulon). These conserved relationships are more likely to contain important cell state regulatory mechanisms (*39*). Profiling of each nuclei’s RNA content showed that we recovered the same cell types as our snRNA-seq dataset, with similar compositionality (fig. S5). In total, we identified 186 high-confidence regulons consisting of 120 activators and 66 repressors that largely display cell type/lineage-specific activity (figs. S6 and S7 and table S8). We found that the L2/3-IS cell state was strongly associated with open chromatin containing MEIS2 binding sites, which is consistent with *MEIS2* being a marker for these cells (fig. S7B). Top regulons specifying L2/3-IS cells other than *MEIS2* (146 genes and 295 regions) were *ZNF595* (18 genes and 44 regions) and *NEUROD2* (27 genes and 32 regions).

We next analyzed COPs and found that they contained not only several regulons shared with OPCs and OLs but also several activator regulons specific for the COP cell state (Fig. 1F). These included *SMAD2* (88 genes/120 regions), a BMP target gene, *LCORL* (28 genes/29 regions), and SOX family members *SOX4* (141 genes/681 regions) and *SOX10* (169 genes/984 regions), which all peak during the COP stage. As such, the global GRE profile of COPs resembles a distinct intermediary between OPCs and OLs (Fig. 1, F and G). Similarly, we also observed dynamic regulation of repressor regulons throughout OL differentiation, with up-regulation of the BMP target *SMAD3* as well as *IRF8*, *GLIS3*, and *ATF7* repressor activity and down-regulation of *ZNF596*, ZNF595, and STAT1 repressor activity (table S8). Consistent with our intracellular communication analysis, these data suggest that the COP state is supported by autoregulatory BMP signaling.

### Identification of human-specific transcriptional divergence in the postnatal DLPFC

**Fig. 2.**
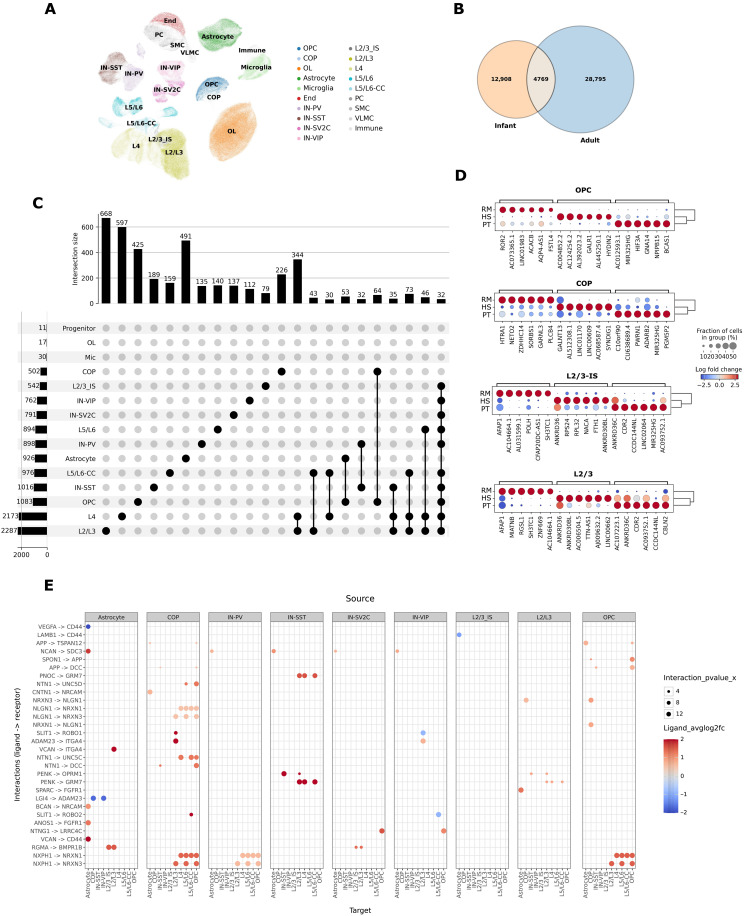
Identification of cell type–resolved human infant–specific gene expression profiles in the DLPFC. (**A**) BBKNN integration and Leiden clustering of infant PFC data with additional adult human chimpanzee and rhesus macaque PFC samples (*26*) totaling 499,245 nuclei. Cell type labels were reannotated following integration with infant datasets due to minor convention differences (see label transfer heatmap in fig. S8). (**B**) Differential expression analysis using pyDESEQ2 (log_2_FC > 0.5; FDR < 0.05). Venn diagram indicates human infant DE genes (versus chimpanzee and rhesus infants; left set) and human adult DE genes (versus chimpanzee and rhesus adults; right set). Counts indicate genes across all cell types. Intersecting genes are DE in the same cell type and in the same direction. (**C**) Upset plot of infant-specific sets from (B). Horizontal bar plots indicate the count of DE genes for the indicated cell type, and vertical bar plots indicate sizes of intersection sets (cell type memberships indicated in linked dots). Genes are considered to intersect if they are DE in the same direction. (**D**) Dot plots per cell type showing examples of top DE genes between species in the infant PFC. Dot sizes indicate the percentage of cells expressing the gene, and colors show the average log_2_FC versus the rest of species. HS, human; PT, chimpanzee; RM, rhesus. (**E**) Cell type–specific LIANA modeling of human infant DE profiles. Dot plot visualization of top LR interactions. The color bar indicates the log_2_FC of ligand expression levels, and increasing dot size indicates the increasing statistical significance of the LR interaction as determined by a Wald test. Each row indicates a unique LR interaction. Each separate panel indicates the source (sender) cell type indicated on top, whereas subcolumns indicate the target (receiver) cell type.

To identify human-specific expression changes that are characteristic of postnatal development, we enhanced our dataset by leveraging additional adult samples [*n* = 4 for each species with similar age ranges and comparable Postmortem interval (PMI) to our infant samples] (table S1) (*26*). Following BBKNN-mediated data integration, the total dataset consisted of 499,245 nuclei, revealing the same cell types and states recovered above (Fig. 2A and fig. S8) (*24*, *26*, *27*). We analyzed differentially expressed (DEA) genes between human, rhesus macaque, and chimpanzee using pyDESEQ [absolute log fold change > 0.5; false discovery rate (FDR) < 0.05] for both infants and adults separately (Fig. 2B and fig. S9A). Comparing human infants to nonhuman primate infants, we detected 10,567 human-specific increases in gene expression (gains) and 7110 decreases in gene expression (losses) distributed across the 15 distinct cell types identified in infants (Fig. 2, B to D; fig. S9A; and table S3). Many of the expression changes specific to human infants (~20 to 50%) were also cell type specific. Such skewing toward cell type–specific expression changes between species has also been noted in other transcriptional comparisons of adult cortical data (*5*, *26*, *31*, *40*) and supports the notion that pleiotropic changes across evolution are generally detrimental and selected against (*41*). As control datasets, we also determined chimpanzee-specific gene expression gains and losses in infant samples (table S3). The enhanced density of the adult data then enabled us to robustly identify 12,908 human infant-specific (7420 gains; 5488 losses) gene expression changes across cell types of which 5840 are unique genes (based on ~45,000 GENCODE features). As a control, we also identified 12,748 chimpanzee infant–specific (7853 gains; 4895 losses) gene expression changes across cell types that were not identified as evolutionarily DE in human or chimpanzee adults (Fig. 2B and table S3). These are also referred to as human infant–specific DE (hiDE) and chimpanzee infant–specific DE (ciDE).

To control for interindividual variability, we flagged hiDE genes that were listed as highly variable between individuals using single-cell transcriptomic data generated in 388 individuals (*42*). Less than 1% of human infant–specific gains or losses were annotated as highly variable, which was not different from the proportion of all genes that are annotated as highly variable (*P* = 0.99, hypergeometric test). This suggests that interindividual variability coupled to low sample size is not a major confounder in our analysis. PMI for the different primate species roughly matched between adults and infants, with human samples having consistently increased PMI. Thus, the infant-specific changes we observed are unlikely to stem from differences in PMI as these would be shared between infants and adults. Furthermore, PMI variable genes were not enriched in our data [*n* = 268/5840 unique infant DEA genes, ~4%; odds ratio (OR) = 0.92 and *P* = 0.95, hypergeometric test]. To ensure that hiDE genes were not skewed due to the wider age range of chimpanzee versus human infants (0 to 5 months versus 2 to 4 months, respectively), we added an additional 0-month human female infant sample from a different dataset (*27*). This generated highly similar results (DEA consistency fraction = ~97 to 99%; Jaccard index = 0.93 across all cell types).

We used gene set enrichment analysis to characterize hiDE genes for each cell type. We found that, among hiDE genes that showed human infant–specific gains in expression that were shared by both COPs and OPCs, there was an enrichment for genes related to (post)synaptic localization and density (table S3). Conversely, human infant–specific gains but not chimpanzee infant–specific gains in COPs are specifically enriched for positive regulators of migration, axon guidance, and apoptosis (table S3). This was not found in OPCs or mature OLs, suggestive of a specific role for COPs in directing synaptic development in humans. In contrast, genes with human infant–specific gains expression specifically in L2/3-IS were most strongly overrepresented by genes with RNA binding activity and various processes related to ribosome biogenesis and translation (table S3). This is consistent with the enhanced size and complexity of human neurons (*43*) and was not specific to L2/3-IS but was also found in other neuronal cell types.

### Human infant–specific expression changes converge on immature OLs and synaptic development

To investigate the intercellular cross-talk specific to postnatal humans, we leveraged our differential expression analysis results to guide the LIANA framework. We used only DE genes for human and chimpanzee as input, which were specific to either infants or adults. A high level of predicted signaling output in human infants came from COPs (*n* = 65 significantly affected LR interactions; specificity rank < 0.05 and magnitude rank < 0.05) (fig. S10A) primarily toward OPCs, L2/3 neurons, and astrocytes. Specifically, OPCs and COPs were characterized by human infant–specific increases in neurexin-neuroligin signaling toward several neural subtypes. These are synaptic cell adhesion molecules that connect presynaptic and postsynaptic neurons at synaptic terminals. This interaction was not observed for infant chimp-specific signaling in OPCs and COPs (fig. S10 and table S4) nor in adults (fig. S11 and table S4). To further confirm that the increased COP-mediated intercellular communication is specifically elevated in human infants and not an artifact of LIANA modeling, we performed the same analysis for other chimpanzee infants and adults and found that such a trend was only found in human infants (figs. S10A and S11A). We also observed a predicted increase in TNFA-ERBB4 signaling between COPs and OPCs that was largely driven by human infant–specific up-regulation of *ERBB4* in OPCs and COPs (table S4). ERBB4, a receptor for neuregulins, is associated with schizophrenia and regulates social experience–mediated myelination and OL maturation, especially during sensitive periods of social conditioning (*20*, *21*). Together, these results suggest that human but not chimpanzee evolution converges on stage-specific processes in infants that are characterized by alterations in immature OLs.

### Human infant–specific gene expression gains in immature OLs are enriched in both ASD risk genes and gene expression losses in patients with ASD

**Fig. 3.**
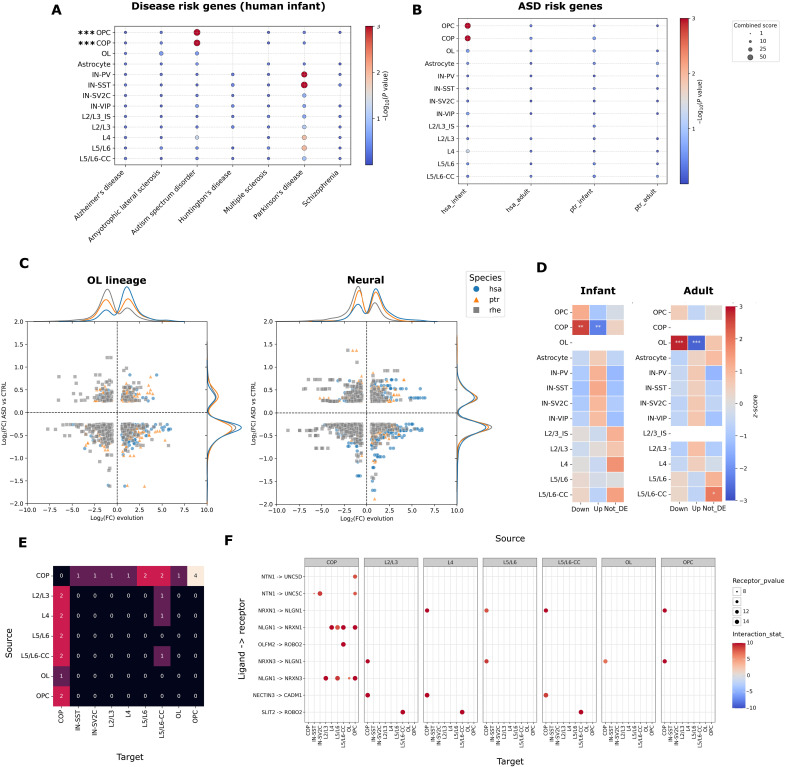
Human infant–specific gene expression gains in OL progenitors are overrepresented in ASD risk genes and gene expression losses in patients with ASD. (**A**) Dot plot per cell type (rows) across representative diseases risk genes (columns) (*47*). Dot size indicates overrepresentation scores calculated by EnrichR (based on OR and *P* value). The color bar indicates the significance (hypergeometric test), and asterisks indicate FDR-corrected *P* values (***P < 0.001). (**B**) Visualization of ASD risk genes from (A) between infant and adult human, chimpanzee, and rhesus macaque. Dots and colors are as represented in (A). (**C**) Scatterplot of evolutionary DE genes in the infant PFC and cell type–matched DE genes from the PFC of patients with ASD (*48*) for the different primate infants indicated by colors. The *x* axis indicates the evolutionary log_2_FC in the species indicated versus the remaining two primate species, and the *y* axis indicates the disease log_2_FC (ASD versus control). Margins indicate the point density along each axis (species normalized). (**D**) Overrepresentation heatmaps (chi-square test of independence) for human infants (left) and adults (right) per cell type (rows), indicating enrichments for expression gains intersecting ASD DE genes. Empty cells indicate that cell types dropped due to low/absent counts. The color bar indicates the *z*-score of residuals, and asterisks indicate the statistical significance (**P < 0.01; ***P < 0.001). (**E**) LIANA sender-receiver matrix showing the number of unique interactions sent by a cell type (rows) and received by a cell type (columns) in putative human infant evolution/ASD interactions. (**F**) Dot plot visualization of human infant gains in (E) showing LR interactions lost in ASD. The color bar indicates the log_2_FC of receptor expression in human infants, and dot size indicates the increasing statistical significance (Wald test). Each row indicates unique LR interactions. Panels indicate the source cell type, whereas subcolumns indicate the target cell type.

As neurexins and neuroligins are strong risk genes for neuropsychiatric disease (*44*), we asked whether infant-specific processes that recently evolved could shed further light on previously proposed interactions between human evolution and neuropsychiatric disorders (*45*, *46*). We compared human infant–specific gains and losses to disease risk genes linked to several neuropsychiatric and neurodegenerative disorders (*47*). We found that human infant–specific gene expression gains in the OL lineage, in particular OPCs (OR = 3.54; FDR = 4 × 10^−6^) and COPs (OR = 3.89; FDR = 2.8 × 10^−5^) were significantly enriched in autism spectrum disorder (ASD) risk genes (Fig. 3, A and B, and table S5). This was not observed for chimpanzee infant–specific gains (table S6) or human gains that were either adult specific (table S6) or shared between adults and infants.

To further substantiate this interaction, we leveraged snRNA-seq data profiling ASD versus control brains (*n* = 63; 33 patients versus 30 controls) (*48*) to obtain an ASD signature set of *n* = 2293 up-regulated genes and *n* = 4735 down-regulated genes over 35 cellular (sub)types (Materials and Methods; table S6). Intersecting our data, we found that our set of human infant–specific gene expression gains was enriched in genes that are down-regulated in patients with ASD in both in COPs (Pearson residual = 23.7; *P* < 0.0001) and OPCs (Pearson residual = 14.08; *P* < 0.0001) (Fig. 3, C and D). This was not observed for chimpanzee infant–specific gene expression gains (table S6). LIANA modeling of these hiDE genes that were also dysregulated in ASD revealed that almost all significant LR interactions (*n* = 24/27) are either sourced from COPs or targeted toward them (Fig. 3, E and F, and table S4). Most LR interactions (*n* = 27/29) predicted signaling changes that involved human infant–specific gains in signaling that are down-regulated in ASD. Similar analysis of adult samples also revealed enrichment of human adult-specific gains in transcriptional changes that are expressed at a lower level in ASD. However, this was only found for mature OLs (Pearson residual = 12.41; *P* < 0.0001). Furthermore, these genes did not include the typical ASD risk genes that were found in the infant dataset. Thus, our data provide evidence for an age-restricted correlation between transcriptional changes occurring in both human evolution and ASD, which particularly involves the OL lineage.

### Human infant–specific gene expression gains in neurons are enriched in PD risk genes and in genes dysregulated in patients with PD

**Fig. 4.**
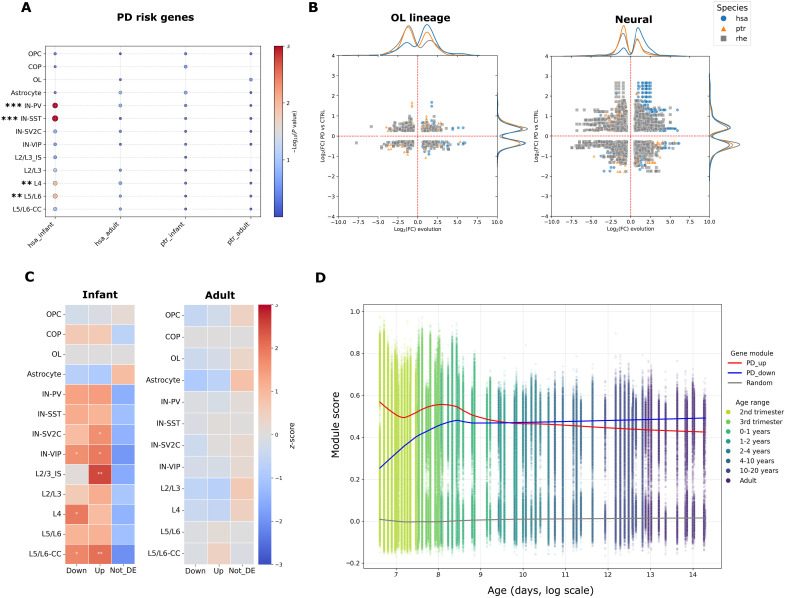
Human infant–specific gene expression gains in neurons are enriched for PD risk genes and genes undergoing transcriptional changes in patients with PD. (**A**) Dot plot per cell type (rows) for PD risk genes (*47*) for infant and adult datasets. Dot size indicates the magnitude of the overrepresentation score based on the OR and *P* value, and the color bar indicates the statistical significance (hypergeometric test with FDR correction, **P < 0.01; ***P < 0.001). (**B**) Scatterplot of evolutionary DE genes in the infant PFC and cell type–matched DE genes from substantia nigra (matched cell types only) between patients with PD and controls (*48*) shown for the different primate infants as indicated by colors. The *x* axis indicates the evolutionary FC per species indicated versus the remaining two primates, and the *y* axis indicates the disease FC (PD versus control). Margins indicate densities of underlying points along each axis (normalized to 1 per species). Data points are colored according to species. (**C**) Heatmaps of grouping overrepresentations (chi-square test of independence) for human infants (left) and adults (right) per cell type (rows). Indicated are enrichments for evolutionary gains compared to overall lineage-matched DE genes between patients with PD and controls. The color bar indicates the *z*-score of Pearson residuals, and asterisks indicate the statistical significance (*P < 0.05; **P < 0.01). (**D**) Scatterplot showing module scores for human infant–specific neural gains reactivated (red fitted line) or down-regulated (blue fitted line) in patients with PD across normal human development into old age (*n* = 374,463 excitatory neurons; 106 individuals) (*24*). The *y* axis indicates normalized module scores of gene sets, with a randomized gene set as background (gray fitted line) for each nucleus (scatter points). The *x* axis indicates the age in years (log_2_ scale). The scatterplot color map indicates the age group ranging from prenatal (yellow points) to adult (blue points).

We also observed an overrepresentation of PD risk genes in human infant–specific gene expression gains across the neuronal lineage (FDR < 0.05, hypergeometric test; Fig. 4A). These gene expression gains were present in several excitatory and inhibitory neural subclasses (Fig. 4A). Similar enrichment of PD risk genes was not observed in human adult-specific gene expression gains or in chimpanzee infant–specific or adult-specific gene expression gains (fig. S12). This suggests that the increased propensity for PD in humans that was previously proposed (*49*) may have an origin in infant-specific processes. The top PD risk genes showing broad neural up-regulation in human infants include *TMEM72-AS1*, *AC119673.2*, and *TSBP1-AS1* (table S5).

We next asked whether these risk genes would gradually reactivate during normal aging in the human brain. We compared our data to recent data tracking gene expression changes in the frontal cortex at cell type resolution into very old age (*50*). There was no activation of these PD risk genes associated with old age except for a single noncoding RNA (*AC119673.2*) in the PARK16 locus. Significant *AC119673.2* up-regulation in human infants was observed across almost all cell types analyzed. This RNA shares a genomic location with *PM20D1*, a gene that inhibits synuclein aggregation (*51*) and transcribes in the opposite direction. Its reduction may also enhance mitochondrial respiration and energy production, which would be beneficial during developmental stages (*52*, *53*).

To further assess the putative link between human evolution and PD, we leveraged an snRNA-seq dataset profiling substantia nigra in patients with PD versus age-matched controls (*n* = 24 patients versus 9 controls) (*54*). Intersecting DE genes between patients with PD and controls with hiDE genes on a lineage-matched basis revealed a notable overlap in neurons (~80% of which were human infant–specific expression gains). This was not found for chimpanzee infant–specific expression gains in neurons (~35% of intersections accounted for expression gains in chimpanzee) (FDR < 0.05, hypergeometric test; Fig. 4, A and B, and table S7). Analysis of directionality profiles revealed that genes with human infant–specific expression gains in neurons are enriched in both PD gene expression gains and PD losses compared to chimpanzee infant–specific expression gains (*P* < 0.001, chi-square test of independence; Fig. 4, B and C). This overrepresentation of PD-dysregulated genes was not specific to a neural subtype as it was found across multiple inhibitory and projection neurons (Fig. 4C). Gene module dynamics throughout human development confirmed enrichment of these genes during early postnatal and late prenatal stages with gradual down-regulation into adulthood, which was not observed in a randomized gene module (Fig. 4D).

Gene set enrichment analysis of neural human infant–specific expression gains that were also expressed to a higher level in patients with PD were enriched for positive regulators of TAU kinase activity (OR = 83.7 and FDR = 3.4 × 10^−2^; table S7) such as the heat shock proteins HSP90AA1 and HSP90AB1. These are essential during neurodevelopment, regulating both neural migration and neurite outgrowth (*55*). Under pathological conditions, these chaperones may instead stabilize disease-associated proteins such as mutated LRRK2, α-synuclein, and Pink1 in Parkinson’s disease, as well as Tau kinases in Alzheimer’s disease (*56*, *57*). Similarly, *SYN3*, a gene that is involved in neurogenesis and axonogenesis and found mutated in behavioral disorders, is part of α-synuclein fibrils in Lewy bodies and a potential therapeutic target in PD (*58*, *59*). This suggests a link between altered axonogenesis during infancy and PD susceptibility later in life. Furthermore, *ERBB4*, which was a human evolutionary gain in OPCs and COPs, as well as *BCAS1*, which is a marker for COPs, are also induced in PD neurons (*60*, *61*). This suggests that the human infant–specific gains in gene expression in the OL lineage cells may play a role in PD susceptibility in neurons later in life.

### A core human infant network that lacks lineage specificity regulates gene sets enriched for ASD-deregulated genes in OLs and PD-deregulated genes in neurons

To analyze what drives the evolutionary emergence of human infant–specific gene expression networks in disease, we refined our analysis to identify TF/gene pairs (nodes) that are coregulated and coupled to open chromatin domains (edges) using our multiome analysis in SCENIC+. We specifically focused on TF/gene nodes representing hiDE gains that were coupled to open chromatin domains containing known human-specific DNA sequence changes, including hINSs, hDELs (*62*), or human accelerated regions (HARs) (i.e., DNA that altered markedly after the split between humans and chimpanzees) (*63*, *64*). This human infant–specific network that was coupled to human DNA alterations in open chromatin is subsequently referred to as the human infant DE core (hiDECORE) network (figs. S9A and S13A).

To validate our hiDECORE open chromatin to target gene interactions that were derived from SCENIC+, we analyzed recently published capture Hi-C data (CHi-C) performed in human neural stem cells (*64*), which identified genes with direct binding to HAR elements. Of the 65 distinct HARs in our hiDECORE peaks that were also captured in the CHi-C dataset, 61 had at least one gene target that was successfully identified in CHi-C (~94%). Furthermore, ~90% (*n* = 55/61) of HAR-to-target gene interactions matched that of our hiDECORE network, indicating a high degree of concordance (fig. S13D). hiDECORE genes were not enriched in variable genes between human individuals in either the neural lineage (*n* = 2/68; *P* = 0.95) or OL lineage (*n* = 1/64; *P* = 0.99) nor were they enriched in male-specific developmental genes (neural lineage hits *n* = 9/68, *P* = 0.99; OL lineage hits *n* = 5/64, *P* = 0.95) (*42*) or PMI-related genes (neural lineage hits *n* = 2/68, *P* = 0.78; OL lineage hits *n* = 2/64, *P* = 0.73, hypergeometric test). To ensure that hiDECORE genes were not affected by the narrower age range of the human samples (2 to 4 months), we added a 2-day-old female human infant sample from a different study, bringing the age range (0 to 4 months) closer to that of the chimpanzee samples (0 to 5 months). Results were still highly similar, with only one hiDECORE gene (*VSTM2L*) not found in this analysis.

We next asked whether the hiDECORE network displays the same cell type specificity typically seen for DE genes. Unexpectedly, we observed high similarity of this hiDECORE network between OL lineage cells and neural lineage cells (~65% overlap of gene expression gains between the two lineages; OR = 15.2 and *P* = 5.98 × 10^−19^, hypergeometric test). In contrast, the expression gains for the OL and neural lineage that were not included in the hiDECORE network because they lacked human-specific sequence changes showed only limited overlap (~12% of unique DE genes). Thus, hiDECORE genes largely lack the cell type specificity that is typically observed for genes with species-specific expression (*5*, *26*, *31*, *40*).

**Fig. 5.**
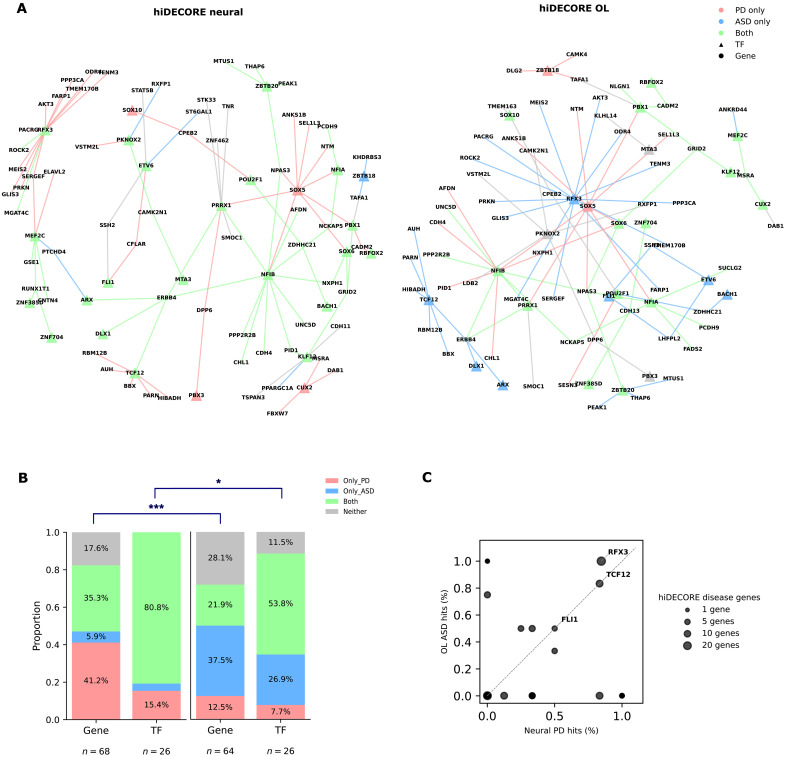
A lineage shared human infant–specific core network (hiDECORE) is enriched in PD-deregulated genes in neurons and ASD-deregulated genes in OLs. (**A**) Visualization of the hiDECORE network and its predicted link to neural disease. From the entire multiome SCENIC+ network of the infant DLPFC, we extracted per lineage all nodes (TFs/genes) that are hiDE gains and correlated to open chromatin regions (single-nucleus ATAC peaks; edges) containing either HARs and/or hINSs/hDELs. We retained TF-to-gene relationships only if (i) the given TF-to-gene relationship was mediated by such ATAC regions of interest, (ii) this TF-to-gene relationship is positively correlated, and (iii) this region-to-gene relationship is also positively correlated. Nodes containing genes or TFs that are also deregulated in ASD are shown in blue, and those containing genes that are deregulated in PD are shown in red. Nodes containing genes that are deregulated in both PD and ASD are shown in green. For visualization of each lineage’s hiDECORE network, only TFs/genes implicated in disease are shown. (**B**) Stacked bar plot quantifying categories per lineage in the overall hiDECORE network, showing the fraction of genes in the hiDE for the neuronal lineage (left) and OL lineage (right) implicated in disease with colors matching those in (A). The hiDECORE lineage difference in disease outcome was quantified by intersecting each lineage’s network with patient snRNA-seq datasets from Figs. 3 and 4, annotating disease status per lineage per disease (disease or not) with Fisher’s exact test (*P < 0.05; ***P < 0.0001). (**C**) Scatterplots quantifying PD neural-dysregulated genes (*x* axis) versus ASD OL-dysregulated genes in gene targets of hiDECORE TFs. Top 3 TFs based on total disease-hit proportions are indicated in the figure.

To further explore the properties of this network, we analyzed the subset of shared hiDECORE TFs between OL and neural lineage cells (*n* = 26 unique TFs; fig. S13A) along with their target genes with genes deregulated in patients with ASD and PD. hiDECORE genes (~80%) were found deregulated in either ASD or PD or in both (Fig. 5, A and B). Despite hiDECORE genes being largely shared between OL lineage and neural lineage cells, we observed substantial divergence of hiDECORE genes in disease overlap (Fig. 5B). Specifically, most hiDECORE OL lineage genes represented genes deregulated in OLs of patients with ASD (*n* = 24; ~38% of hiDECORE OL target genes), which is significantly enriched versus hiDECORE neural genes (*n* = 4 dysregulated genes in ASD neural cell types; ~6% of target genes) (OR = 9.6 and *P* < 0.001, Fisher’s exact test). We also observed this enrichment when analyzing TFs only (*n* = 7/26 TFs versus 1/26 neural TFs; OR = 9.2 and *P* = 0.024). Conversely, there was an inverted enrichment in the neural lineage hiDECORE genes for PD-dysregulated neural genes (*n* = 26; 41%) versus OL lineage hiDECORE genes (*n* = 8, ~12% of target genes; OR = 4.9 and *P* < 0.001, Fisher’s exact test; Fig. 5B).

This indicates that, although the core TF network itself lacks cell specificity, it has lineage-specific manifestations that are enriched for different disease-associated genes. For instance, *NFIB*, which is a known regulator of the neurogenic-to-gliogenic switch in mice and acts as a brake on OL differentiation (*65*), targeted common PD- and ASD-associated genes in both cell types. In contrast *TCF12*, a gene that is linked to intellectual disability and required for OL proliferation (*66*), targeted mostly PD-deregulated genes in neurons and ASD-deregulated genes in OLs (Fig. 5, A to C). Collectively, these results demonstrate that infant-specific expression signatures that recently evolved in humans can manifest as distinct disease-associated signatures in a cell type–specific manner.

## DISCUSSION

Although human postnatal development contains several sensitive periods affecting visual and auditory development as well as language acquisition and social conditioning (*20*, *21*), the human-specific processes during these sensitive periods have largely remained elusive. Here, we leveraged two rare infant chimpanzee samples to shed light on putative human-specific processes during early postnatal development. Although we find several interesting interactions, our study is limited by reduced infant sample size and several interprimate variabilities that are difficult to control. For instance, although our samples all fall withing the same developmental stage matched between primates (*22*), precise developmental state matching between primate species is challenging. Furthermore, species-related ambiguities in matching precise cortical areas to functionally conserved domains can similarly lead to noise in the data.

Nevertheless, when analyzing early postnatal stages of development, we find infant-specific cell states in L2/3 neurons and expansion of BCAS1+ premyelinating OLs across primates, enabling their robust characterization. These cells have recently been described as COPs (*31*) in single-cell analysis; however, their status as being fully committed to myelination is still unclear (*29*). We find that COPS but not OPCs or OLs are robust senders of BMP signals to various cell types in the brain including autoregulatory signals, suggesting that their function could be supportive as opposed to being a transient cell state. In addition, human infant–specific gene expression gains contain COP marker genes and *BMPER*, a rheostat for BMP signaling (*36*), suggesting that the infant COP state may be stabilized in humans. As BMP signaling limits OL differentiation and myelination (*67*), these cells could play a role in the delayed myelination and neoteny observed in human infants (*1*).

In addition, we find human-specific cross-talk between COPs, OPCs, and neurons involving neurexins and neuroligins, which are synaptic proteins that aid maturation of synaptic junctions indirectly affecting synapse stability and are strongly associated with neuropsychiatric diseases in humans (*68*). Notably, ASD has been genetically linked to human evolution (*45*, *69*). Expanding on this, we show that human-infant but not human-adult gene expression gains are overrepresented in ASD risk genes as well as in genes deregulated in brains of patients. These genes converge on COPs and OPCs in infants, with a weaker signal in adult OLs that does not involve the typical risk genes. As many of these genes have a temporal profile peaking in infancy, this explains why these interactions have remained invisible in adults. ASD gene mutations in OLs can contribute to ASD-like pathology in mice (*70*). Nevertheless, as ASD is mainly characterized by altered gene expression in neurons (*71*) and also involves other cell types such as astrocytes and microglia, it is unclear whether and how the evolved gene expression signatures described here in OLs are causally related to the disease.

Last, we find an unexpected correlation between human evolution and Parkinson’s disease, with human infant–specific gene expression gains in neurons enriched for PD risk genes as well as genes with altered expression in patients with PD. It has been suggested that humans are exceptionally vulnerable to neurodegenerative diseases including PD as similar disease pathology has not clearly been observed in other primate species (*49*, *72*). We show that the human infant–specific expression changes that are overrepresented in PD-deregulated genes are enriched in synaptic developmental genes. Imbalances in synaptic function can strongly affect neural vulnerabilities, especially in larger projection neurons. As such, pathological deregulation of such a program may play a role in PD susceptibility. It is interesting that our findings connect human evolution to Parkinson’s disease only and not Alzheimer’s disease as the latter has also not clearly been observed in other primates (*72*). Substantia nigra pars compacts (SNc) dopaminergic neurons have extraordinarily large, highly ramified axonal trees that require extensive maintenance of axonal transport, synaptic vesicle cycling, and mitochondrial distribution. This increases vulnerability to disruptions in these processes and in proteins implicated in PD (e.g., PINK1/Parkin and α-synuclein) and increases dependency on OLs for support (*73*). As such, α-synuclein pathology in OLs can similarly lead to loss of dopaminergic function in multiple system atrophy. Instead, AD-vulnerable neurons have different energetic profiles, likely making them less sensitive to the same insults (*74*).

To explain what changes may have driven the putative link between human infant evolution and disease, we identified a core network of human infant–specific expression changes that are characterized by human-specific DNA sequence changes in regulatory DNA. Unexpectedly, this network lacks the lineage specificity that is typically observed for newly evolved regulatory DNA and, as such, operates both in the OL and neuronal lineage, having different downstream putative target genes in each lineage. In the OL lineage, these downstream target genes are overrepresented in ASD risk genes, whereas in the neural lineage, PD genes are enriched. It is therefore possible that these pathologies could be maintained in the human lineage by complex antagonistic interactions across developmental stages. Patients with ASD are also more likely to develop PD (*75*, *76*), a link that is currently not understood. Our analysis linking the two diseases through a common network could provide insight into common biology between the two diseases. Thus, our study provides unique datasets, shedding light on a previously inaccessible stage of human evolution and discovering human infant–specific processes as well as clues as to human-specific propensities to neural disease.

## MATERIALS AND METHODS

Full methods accompany this paper (see Supplementary Text). Briefly, human brain samples were obtained from the Netherlands Brain Bank (NBB) (http://brainbank.nl/) and the NIH Human Brain Collection Core (HBCC; project number: 20181116) (table S1). Informed consent was acquired, meeting all ethical and legal requirements for autopsy, tissue storage, and use of tissue and clinical data for research. Primate samples were obtained from the Biomedical Primate Center [BRPC; http://bprc.nl/ (veterinary control number: 7962)]. No primate animals were experimented on or euthanized for the purpose of this work.

The frozen PFC was dissected from cortical slabs in a cold room on dry ice including using tissue punches to include all cortical layers followed by dissection to remove most of the underlying white matter. The tissue was pulverized using a precooled pestle and mortar on dry ice and homogenized using a glass douncer. To separate the nuclei from other cell organelles, an OptiPrep density gradient was used followed by sorting using a BD FACSAria Fusion flow cytometer to sort for single DAPI-positive nuclei while removing leftover debris and doublets. To limit the influence of technical variation, we combined cells from separate species on a 1:1 cell count basis per sequencing sample for processing using the 10x Chromium V3 Single Cell RNA Gene Expression kit according to the manufacturer’s protocols. Nuclei were loaded, aiming to recover 10,000 nuclei per sample. Single-cell RNA-seq samples were sequenced on an Illumina HiSeq 2500. Single-cell libraries for multiome analysis were prepared using the Single Cell Multiome ATAC + Gene Expression kit (10x Genomics), and 10,000 nuclei were loaded per lane. Multiome samples were sequenced using the NovaSeq 6000 platform.

## Supplementary Materials

### The PDF file includes:

Supplementary Text Figs. S1 to S13 Tables S1 and S2 Legend for data S1 References
