# Supplementary material for: Charting the human-specific properties of gene expression networks in the infant prefrontal cortex
Source: Sci Adv. 2026 Jun 3;12(23):eaea3316. doi: 10.1126/sciadv.aea3316 (PMC13232592; doi:10.1126/sciadv.aea3316)
Supplement: Supplementary file 1 — Supplementary Text Figs. S1 to S13 Tables S1 and S2 Legend for data S1 References [file sciadv.aea3316_sm.pdf]

Science Advances
AAAS

Supplementary Materials for

Charting the human-specific properties of gene expression networks in the infant prefrontal cortex

Jonathan Klavert *et al.*

Corresponding author: Menno P. Creyghton, m.creyghton@erasmusmc.nl

Sci. Adv. **12**, eaea3316 (2026)
DOI: 10.1126/sciadv.aea3316

The PDF file includes:

Supplementary Text
Figs. S1 to S13
Tables S1 and S2
Legend for data S1
References

Other Supplementary Material for this manuscript includes the following:

Data S1

Supplementary Text

Sample collection

Human prefrontal cortex samples were obtained from the Netherlands Brain Bank (NBB) and the Human Brain Collection Core (HBCC) with informed consent obtained for all human donors. Prefrontal cortex samples from adult chimpanzees were resampled from our previous work(*4*, *11*) and dorso-lateral prefrontal cortex (DLPFC) samples from infant chimpanzee and macaque were collected at the Biomedical Primate Research Centre (BPRC) in Rijswijk, the Netherlands (http://www.bprc.nl), roughly corresponding to Brodmann area 46/9 in humans also see Table S1 below. Human infant samples from the HBCC were similarly sampled from Brodmann area 46/9 which at the infant state are hard to distinguish. All non-human primate samples represent rest material involving no experimentation. Pathological examination revealed no signs of brain abnormality or disease.

Nuclear isolation and sample preparation

Frozen prefrontal cortex was dissected from cortical slabs in a cold room on dry ice including using tissue punches to include all cortical layers followed by dissection to remove most of the underlying white matter. 50 mg of tissue was pulverized using a precooled pestle and mortar on dry ice. Powdered tissue was then homogenized by vortexing in 2 mL EZ buffer (Nuclei isolation kit, Sigma NUC101) using a glass douncer (Kontes Glass Co.), followed by a 5' incubation on ice. Homogenates were centrifuged for 15' at 100 x g at 4 ºC, resuspended in 2 mL EZ buffer and incubated for 5' on ice. Samples were centrifuged again for 15' at 65 x g at 4 ºC, resuspended in 4 mL nuclear suspension buffer (NB: 0.01% BSA (Sigma, A9418), 1x 'complete' protease inhibitor cocktail (Roche, 11873580001) in MQ water) and filtered through a 40 µM cell strainer (Falcon, 352340). Following this, samples were centrifuged for 15' at 65 x g at 4 ºC. To separate the nuclei from other cell organelles a density gradient was created with OpitPrep (Sigma D1556). Pellets were resuspended in 30 mL NB or cold PBS after which 10 mL of 30% OptiPrep was carefully added at the bottom with a syringe and needle, followed by another 5 mL layer of 60% OptiPrep underneath. Samples were centrifuged for 15' at 1,000 x g at 4 ºC. Nuclei were retrieved from between the 30% OptiPrep and PBS layers with a syringe and needle and checked for quality under a bright field microscope. Nuclei were stained using Hoechst-33342 (ThermoFisher, H3569, 1:1000 dilution) and filtered through a 35 µm strainer (Corning, CLS352235) before sorting. Sorting was performed using a BD FACSAria Fusion flow cytometer to sort for single DAPI+ positive nuclei while removing leftover debris and doublets. Notably sorting also reduces the influence of ambient

RNA(*23*). After sorting, nuclei were spun down for 5 min at 500×g and resuspended in PBS + 1% BSA to be loaded on the 10x microfluidic chip device. To limit the influence of technical variation we combined cells from separate species on a 1:1 cell count basis per sequencing sample for processing using the 10x Chromium V3 single cell RNA Gene Expression kit according to the manufacturer's protocols. Nuclei were loaded aiming to recover 10,000 nuclei per sample. Single cell RNA-Seq samples were sequenced on an Illumina Hiseq 2500.

For multiome single cell data, nuclei from one infant human brain and one infant chimp brain was prepared as described above for RNA-seq with minor changes. Specifically, 0.01% digitonin was added to the lysis buffer, and 2 U/µL RNase inhibitor (Roche) was added to all nuclear preparation buffers. After preparation of nuclei, droplets and single cell libraries were prepared using the Single Cell Multiome ATAC + Gene Expression kit (10x Genomics) and 10,000 nuclei were loaded per lane. Multiome samples were sequenced using the NovaSeq 6000 platform.

<u>snRNAseq data preprocessing, species demultiplexing, and ambient RNA removal</u>

Raw snRNAseq data from 10x Genomics platform was processed using cellranger (v6) pipeline. BCL files were converted into FASTQ reads using cellranger-mkfastq under default settings. In order to minimize non-biological sources of variation which are well described in snRNAseq studies, different species were combined per individual sequencing run. Species identity per nuclei was recovered by separate alignments to human (hg38), chimpanzee (pt3), and rhesus (rm8) genomes obtained from Ensembl database (https://www.ensembl.org/info/data/ftp/index.html) using HISAT2 with default settings (mismatch penalty = 6, minimum alignment score = 20% of read length). For species annotation we retained only nuclei barcodes with high species-specificity (>7% difference in genome alignments) to remove potential cross-species doublets, and removed nuclei if its best alignment to either genome was below 40% (Fig. S1A).

snRNAsq reads were then aligned to a humanized consensus genome generated in this study. FASTA files for both human (GRChg38) and chimpanzee (PanTro3) genomes were downloaded from Ensembl database and aligned to each other using GSAlign with default parameters (minimum sequence identity = 70, maximum indel size = 25). Diverged sites on the human genome were then masked using BEDtools (v2.3) in order to create the consensus genome in hg38 coordinates. For gene expression quantification, we used cellranger-count pipeline (v6) with –include-introns flag and otherwise under STAR aligner default settings (max. mismatch = 0.04, minimum intron length = 20) for a total of 59,120 nuclei.

For ambient RNA removal, raw gene-by-cell count matrices from cellranger-count were used for training CellBender (v0.3.1) in identifying likely sources of ambient RNA contamination (training epochs = 150, false positive rate = 0.01). We further performed subcluster cleaning in order to remove nuclei with high levels of identified ambient RNA. We removed all nuclei containing less than 2,000 UMI or 500 detected genes, along with those containing >5% mitochondrial genes, leaving a total of 37,994 nuclei successfully passing all QC and CellBender subcluster cleaning.

Dataset normalization, dimension reduction, and batch integration

Downstream snRNAseq analysis was performed in scanpy (scanpy v1.10; python v3.9) using standardized workflows. Following QC filtering each nuclei's raw gene counts were depth-normalized using the scanpy.*normalize_total* function under default settings per 10,000 unique UMIs (target_depth = 10,000). Depth-normalized counts were further processed by natural log-transformation using scanpy.*log1p* function prior to dimensionality reduction.

For dimension reduction, we utilized scanpy.*highly_variable_genes* function under default settings (n_top_genes = None, max_mean = 3, min_mean = 0.0125, min_dispersion = 0.5, max_dispersion = None). Following HVG selection we performed principal component analysis (PCA) using the scanpy.*pca* function under default settings (n_pcs = 50, svd_solver = 'arpack', zero_center = True). Following PCA, nuclei from all species and batches were integrated using batch-balanced k nearest neighbour (BBKNN)(*25*) software (v1.3.6) under settings (batch_key = species + age group, n_pcs = 30). Subsequently, an integrated UMAP embedding was drawn using scanpy.*umap* function under default settings (min_dist = 0.5, spread = 1, n_components = 2, gamma = 1) and cell type clusters were detected using scanpy.*leiden* function under default settings (flavor = 'leidenalg', resolution = 1, n_iterations = -1). However, as under default resolution settings we find cluster splitting with no well-defined markers, we proceeded with lower resolution clustering throughout the study (0.5-0.8, depending on dataset combinations).

Cell type annotation and validation

Following leiden cluster detection, we cross referenced each cluster's top (positive) marker genes obtained using scanpy.*rank_genes_groups* function under default settings (groupby = 'leiden', groups = 'all', reference = 'rest', method = 't-test_overestim_var', corr_method = 'benjamini-hochberg') against commonly known cell type markers obtained from literature(*26*, *27*). Following marker analysis, we opted to re-cluster the cells under lower resolution (0.5), as differences in marker expression between closely related leiden clusters at default resolution=1 were not evident.

In order to ensure our findings are robust to differences in analysis methods, we further performed the above steps in Seurat (v4, R 4.3)(*77*). Datasets were depth-normalized using *NormalizeData* and transformed to unit variance using ***ScaleData*.** Integration was performed with canonical correlation analysis (CCA) via *FindIntegrationAnchors* (dims = 1:30, k_anchor = 5, k_filter = 200, k_score = 30), followed by clustering (*FindNeighbors and FindClusters)*. Cluster markers were obtained with *FindAllMarkers* function followed by comparison with known cell type specific markers(*26*, *27*).

Comparison of the two workflows was performed by recovering each nuclei's cell type label in the Scanpy workflow and the same nuclei's cell type label in the Seurat workflow represented as a confusion matrix (Fig. S1D). Coherent and incoherent classifications were then quantified and shows >97% coherence between the two frameworks in cell type labeling. We opt to proceed with Scanpy workflow for all further steps due to performance considerations with atlas-level datasets in later sections.

Integration with public snRNAseq datasets

Additional human, rhesus macaque and chimpanzee adult PFC data(*26*) was downloaded from the Gene Expression Omnibus (GEO (GSE206994)) by obtaining the raw barcode-gene count matrix from along with each metadata table. Raw counts were depth-normalized and log-transformed in Scanpy, and integrated with our infant datasets using BBKNN under settings (batch_key = species + age group, n_pcs = 30) as described above for the infant datasets (Fig. 3A). For cell type label transfer/query with public datasets, we generated confusion matrices with a cell's newly called Leiden cluster and its prior label.

Human brain atlas data covering a variety of human developmental and adult stages(*24*) was downloaded in similar fashion (https://cells.ucsc.edu/?ds=pre-postnatal-cortex), separating the glial and neural lineages. As a negative control for integration overfitting, we included an extra cell type not present in the atlas from our dataset (e.g. our interneurons in excitatory neuron integration) to verify that this extra cell type would remain separated in the newly integrated embedding.

Differential expression analysis

Differential expression (DE) analysis (DEA) was performed by pseudo bulking cell types using pyDESEQ2 (v0.5.0), as single-cell DE analysis methods have been shown to generate large numbers of false-positives and sample/batch-specific biases(*78*). For each cell type we aggregated gene counts per condition (species + age group) that were randomly redistributed into three pseudo-replicates to better analyze rare cell types and mitigate the effects of interindividual variability in

cell number. Following pseudo-replicate generation, pyDESEQ2 was run under default settings (design = "~condition", refit_cooks=True) and retaining only genes with total counts > 10 in our dataset. DEA between the three primate species was performed in three pairwise comparisons covering all possible combinations. Statistical significance was determined by a Wald test result of absolute(log2foldchange) > 0.5 and Benjamini-Hochberg adjusted (p-value) < 0.05.

To ensure that our approach did not yield spurious results, we ran both pyDESEQ2 without pseudo-replication (by aggregating counts per biological sample, per cell type) and dreamlet under default settings(79) to assess condition effects per age group and obtained similar LOGFC distributions We further compared effect sizes of each model along with overlap of DE genes per cell type to assess consistency fraction and correlation of findings, revealing nearly identical effect size (Pearson's r = 0.99 for DESEQ pseudobulk; r = 0.95 for dreamlet pseudobulk) between methods and overall DEA recovery (Fig. S9B; DEA consistency fraction ~80-99% per cell type (~70%) consistent hits (matching both cell type and direction). Jaccard index was 0.65 for pseudobulk and 0.38 for dreamlet, the latter likely reflecting limited performance of LIMMA-based approaches for low n sample analyses (*80*)(*81*). Finally we made sure our data was not driven by individual variability by analyzing enrichment for genes with high interindividual variabilities(*42*).

As confounder analysis is challenging using low n samples, we employed several additional checks to deal with these. To mitigate batch effects, we mixed human and non-human primate samples in single batches (see species demultiplexing) as shown previously (*12*). As human samples have typically higher post mortem interval (PMI), PMI was selected to match between adult and infant samples (See Table S1) and as such is not expected to contribute to infant specific genes. Sex was maintained constant for infant samples. Genes linked to interindividual variability or skewed towards sex were flagged based on a study of 388 human brains(*42*) and not found enriched in any of our gene sets.

Variance partitioning on our data was performed using LinearRegression module from scikit-learn (v1.4.1) on depth-normalized, log transformed counts, pseudobulked per individual per cell type with GLM design (Expression ~ Species + donor + batch + n_genes + cell type + PMI) shows that PMI explains the least variation overall (median 0.4% variance across all genes), followed by n_genes (2.2%), sequencing batch (2.2%), individual (2.6%), species (3.6%), and cell type (30.3%). Genes flagged to be correlated to n_genes (n= 1,192 genes, top ~5% of all tested genes) are not overrepresented in our human infant DEA (n= 191/5,840 unique human infant DEA genes, ~3% of

DEA genes; OR= 0.65, p= 1.0 hypergeometric test). Neither is batch (n=207/5,840, ~3% of DEA genes; OR = 0.71, p=1 hypergeometric test) nor PMI (n=268/5,840 unique infant DEA genes, ~4%; OR = 0.92, p=0.95 hypergeometric test). Conversely, the global linear model reveals that top 5% of species-driven genes (median variance captured = 19%) are significantly overrepresented in our human infant DEA (n=409/5,840, ~7%; OR=1.4, p=2.9e-15 hypergeometric test) and the top 5% of cell type-driven genes (median variance captured = 67%) are also significantly overrepresented in our human infant DEA set (n= 439/5,840; ~7.5%; OR=1.49, p=1.4e-21 hypergeometric test).

Species-specific gains or losses were categorized on basis of being uniquely upregulated or downregulated compared to the other two species (Fig. S9A). Primate adult DEA was performed using public data only(*26*) to entirely remove potential confounders between studies/labs. Following definition of species-specific gains and losses per age group (infant and adult), we compared between infant DEA and adult DEA within each species to address stage-specific changes in a cell type matched fashion. Genes were considered to overlap in comparisons if differential expression matched both in the same cell type and the same direction (i.e. gains or losses).

<u>Gene set overrepresentation analysis</u>

Gene set overrepresentation was performed using pyGSEA (v1.1.2) utilizing the EnrichR module. Gene lists of interests (i.e. infant hDE, infant hDE x ASD/PD, cell type markers, etc.) were queried from three Gene Ontology databases (biological processes, molecular function, cellular component; 2023). Significant enrichment was defined as FDR q < 0.05 of a hypergeometric test (versus all detected genes as background) under default settings, and all such terms were retained.

Due to evolutionary symmetry in DEA profiles (i.e. genes representing human gains will represent losses in chimpanzee or rhesus), genes were separate based on evolutionary direction (gain/loss) as well as disease directionality (upregulated/downregulated). For plotting top enriched terms which are unique to given cell type/lineage, EnrichR output per cell type was aggregated into a dictionary and processed for those which are not redundant across cell types.

<u>Intercellular communication modeling</u>

Intercellular communication was performed using LIANA, to predict overrepresented ligand receptor (LR) interactions, opting to use their *rank_aggregate* function (consensus score 7 different cell-cell communication tools and 16 resources) under settings (min_proportion = 0.05, specificity rank = 0.05, resource_name='consensus', complex_col='stat'). The significant cell type-LR

dictionary output was then processed to annotate those LR interactions which show cell type-specificity.

For baseline modeling of cell type signaling profiles, all nuclei were aggregated per cell type regardless of species to investigate evolutionarily conserved profiles. For condition-specific modeling of signaling profiles, we utilized the DEA output and extracted nuclei for this condition prior to running LIANA with the same thresholds of *rank_aggregate* function (consensus score 7 different cell-cell communication tools and 16 resources) under settings (min_proportion = 0.05, specificity rank = 0.05, resource_name='consensus', complex_col='stat'). For plotting select terms, we either select top n LR interactions based on a combination of magnitude_rank, specificity_rank and cell type specificity, or in some instances plot all significant LR interactions remaining when numbers allow.

<u>Multiome data processing and SCENIC+ gene regulatory network modeling</u>

10x multiome read files were processed using CellRanger-ARC (v2.0) aligned to the consensus genome under default settings (--include-introns=True, --arc_gex_exclude_introns=False). Following generation of gene-cell count matrices and snATAC fragment files per barcode, we utilized the SCENIC+ framework for GRE modelling (v1.0a1)(*38*). Firstly, RNA data is processed in a standard scanpy workflow as described for scRNA sequencing analysis. Following QC filtering each nuclei's raw gene counts were depth-normalized using the scanpy.*normalize_total* function under default settings per 10,000 unique UMIs (target_depth = 10,000). Depth-normalized counts were processed by natural log-transformation using scanpy.*log1p*.

Dimension reduction was performed using the scanpy.*highly_variable_genes* function (n_top_genes = None, max_mean = 3, min_mean = 0.0125, min_dispersion = 0.5, max_dispersion = None). Following HVG selection we performed principal component analysis (PCA) using scanpy.*pca* (n_pcs = 50, svd_solver = 'arpack', zero_center = True).

Nuclei from all species and batches were integrated using BBKNN(*25*) (v1.3.6) under settings (batch_key = species + age group, n_pcs = 30). The integrated UMAP embedding was drawn using scanpy.*umap* (min_dist = 0.5, spread = 1, n_components = 2, gamma = 1) and cell type clusters were detected using scanpy.*leiden* (flavor = 'leidenalg', resolution = 1, n_iterations = -1).

Following leiden cluster detection, we cross referenced marker genes obtained using scanpy.*rank_genes_groups* (groupby = 'leiden', groups = 'all', reference = 'rest', method = 't-test_overestim_var', corr_method = 'benjamini-hochberg') against known cell type markers(*26*, *27*). Following marker analysis, we re-clustered cells under lower resolution (0.5), as differences in marker expression between closely related leiden clusters at default resolution=1 were not evident.

Following annotation of cell types based on RNA gene counts, ATAC fragment files were aggregated based on RNA directed cell type identity and peak calling was performed using MACS2 (genome_size = 'hs', shift=73, extend=146) per cell type. Performing peak calling separately per cell type has been shown to increase recovery of snATAC peaks specific to rare cell types. snATAC libraries were further filtered based on number of unique fragments with automatically determined thresholds (n= ~10,000-15,000 unique ATAC fragments) and TSS enrichment (> ~7).

Following cell type-specific peak calling, a nonredundant consensus peak set was generated (half_width=500 bp) to create the input peak-cell matrix for latent drichlet allocation (LDA) topic modelling. Topic modelling was performed using pyCisTopic (v2.0a0) to identify coregulated snATAC peak sets which explain cell type variability. A range of topic numbers [2,4,8,16,32,48] were tested and diagnosed for best model fit. We utilized four metrics commonly used in topic modeling, namely Minmo, log-likelihood, Arun, and Cao_Juan. Although there was no consensus best fit between all four metrics, we proceed with the 48-topic model as three of the four metrics find this to be the best fit. Binarization for region-topic assignment was then performed by taking the top 3,000 regions per topic using built in *binarize_topics* function from SCENIC+ (target = 'region', method = 'ntop', ntop= 3000). Cell-topic assignment was done using the same function targeting cells (target = 'cell', method = 'li', bins = 100, smooth_topics = True).

Topic region sets were then annotated for TFs with enriched motifs using pyCisTarget (v1.0a2). Briefly, TF motifs were scored using ClusterBuster on SCREEN region hg38 reference for a given set of regions. Differentially enriched motifs (DEM) for each topic were quantified based on area under the curve (AUC) recovery, and a normalized enrichment score (NES) was calculated based on AUC average of all other motifs – keeping only those TFs with motif NES > 3.

TF-region to region-gene relationships were resolved with GRNboost2, by quantifying the importance of TFs and regions in relation to their target genes using linear correlation. Integrated outputs of each co-expression modality were then integrated to DEM results to formulate eRegulons (i.e. a TF with its linked regions and target genes), in which only high-confidence interactions

(importance score < 0.05) were retained. Furthermore, we only retained clear activator (TF-gene +/+; Region-gene +/+) or suppressor (TF-gene -/-; Region-gene -/-) eRegulons.

Interactions with disease risk genes and disease differentially expressed genes

Neural disease risk genes were obtained from BrainBase(*47*) (https://ngdc.cncb.ac.cn/brainbase/index) as shown by others(*4*). Significance of enrichment was quantified using EnrichR module as before for GO analysis (i.e. hypergeometric test, using all detected genes as background). Multiple testing correction was performed using Benjamini-Hochberg FDR correction implemented in EnrichR. We performed such overrepresentation testing of disease risk genes per cell type and per condition, focusing only on upreg2ulated genes (gains). snRNAseq datasets for prefrontal cortex in ASD patients (n= 33 case vs. 30 controls) were obtained directly from the enclosed DEA tables(*48*). The DEA results (ASD up and ASD down) were obtained for intersection with our evolutionary DEA gene lists per each condition. DEA intersections were performed on a lineage-matched basis (i.e. neurons, oligodendrocytes, astrocytes, microglia) to ensure the evolutionary-disease interaction is biologically relevant not confounded by differential subtype calling.

snRNAseq datasets for PD patients analysing substantia nigra (n= 23 case vs. 10 age-matched controls)(*54*) was obtained directly from prior DE analysis. DEA results were obtained from supplementary materials and intersected with our DEA on a lineage-matched basis. Significance of overlap enrichment was quantified by creating count tables for each condition, per lineage/cell type (grouping rows) and for each disease DE directionality (outcome columns). The resulting contingency tables (in units of raw gene counts) were then analysed with a chi-square test of independence using the built in *chi2_contingency* function from sciPy (v1.14.0). As chi-square tests require mutually exclusive groupings, contingency tables were generated separately for evolutionary gains and losses to ensure no duplicated genes emerged across evolutionary comparisons. Furthermore, evolutionary DEA groupings (rows) were dropped prior to chi-square calculations if expected counts were below 5 as chi-square approximations are known to be biased in low count ranges. Deviations of each cell from expected ranges were calculated with Pearson residuals ([observed-expected]/[sqrt(expected)] for each cell in the contingency table, followed by z-scoring (per column) to quantify groups (rows) relative to each other. P-values were obtained by obtaining right tailed probability densities under a standard normal distribution after z-scoring.

Human-specific genomic features/structural variants and network analysis

Analysis of human-specific genomic features for analysis of open chromatin domains comprises several human specific region definitions, including human accelerated regions (HARs)(*63*), human-specific insertions and deletions (hINS and hDEL)(*62*). Region BED files were downloaded and intersected with gene sets of interest (DE analyses) or multiomic snATAC peaks (TF network analyses). Human specific region definitions described above are collectively called human regions of interest (hROI).

Overrepresentation of such features in DEA gene sets was calculated using a Fisher's exact test, comparing overlap proportions in human infants (per cell type) versus chimpanzee infants as control in cell type matched DEA gene sets. Statistical significance was determined using FDR $q < 0.05$ after Benjamin-Hochberg multiple testing correction.

TF network modelling was performed by subsetting our global SCENIC+ analysis by the following inclusion criteria. For each lineage network of interest, we extracted all regulon components (i.e. individual TF-region-gene triplets) along following requirements depending on which network is assessed. For instance, in generating the human Decore network requirements are: (i) Either the TF or target gene in a triplet represents a human infant-specific gain, (ii) the region (snATAC peak) underlying this TF-gene interaction contains a hROI, (iii) both TF-gene and region-gene relationships are correlated as determined by GRNboost2 (putative enhancer). In the case of disease interactions this same TF/target gene is dysregulated in disease of interest. Such network modelling was performed on the evolutionary DEA (and disease DEA) profiles on a per lineage basis to address lineage convergent and divergent network components in both evolutionary and disease interaction. Human core networks were then visualized using NetworkX software (v3.4.2).

Statistical significance in interlineage distribution of disease implicated genes/TFs was performed using a Fisher's exact test in sciPy (v1.14.0). 2x2 contingency tables were constructed where each row represented a lineage's hiDEcore outcomes (disease or not disease), separately for genes and TFs, and two-tailed p-values and odds ratios were obtained. This enrichment was quantified per disease state.

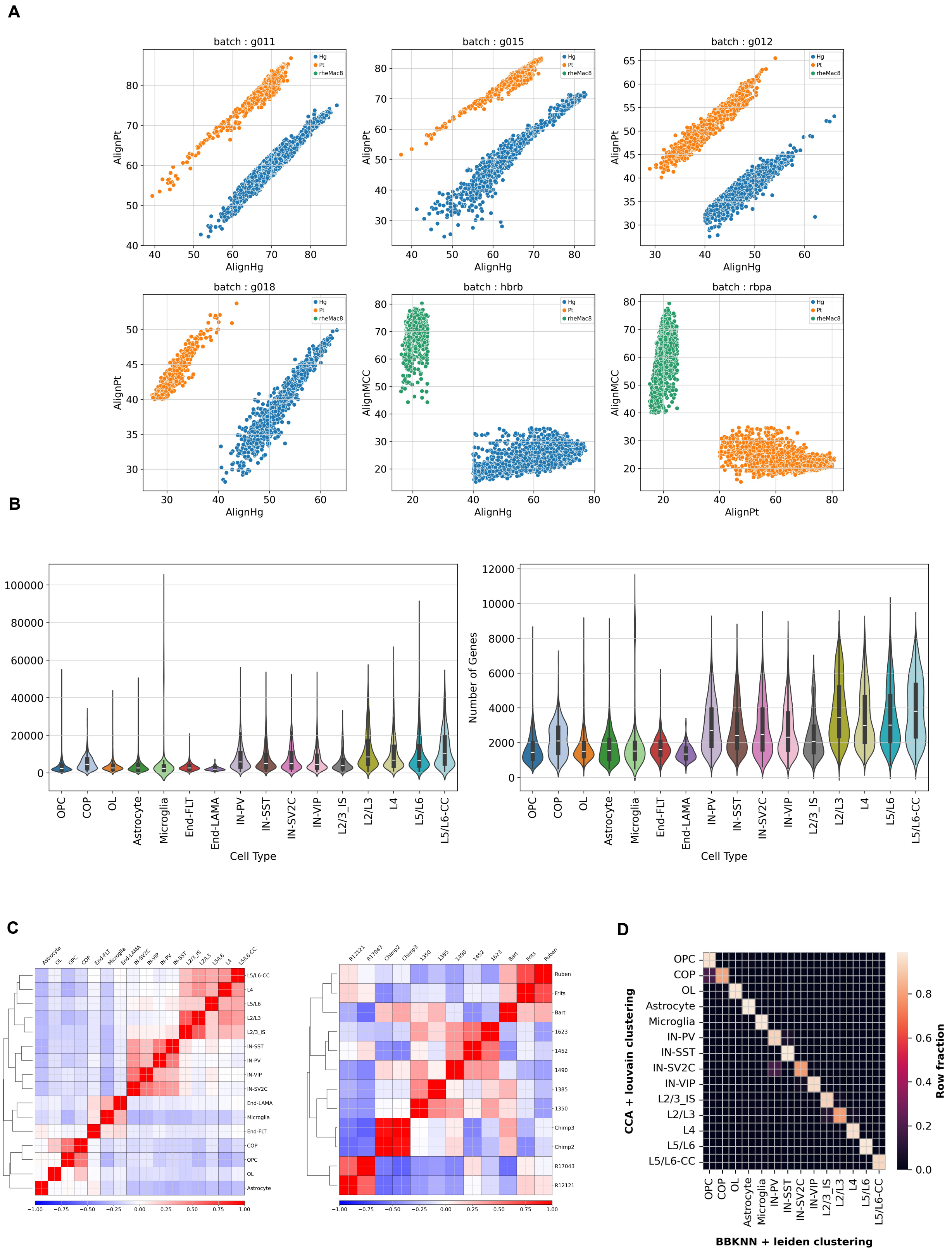
A
batch : g011
batch : g015
batch : g012
batch : g018
batch : hbrb
batch : rbpa
Hg
Pt
rheMac8
AlignPt
AlignHg
AlignMCC
B
Total Counts
Number of Genes
Cell Type
OPC
COP
OL
Astrocyte
Microglia
End-FLT
End-LAMA
IN-PV
IN-SST
IN-SV2C
IN-VIP
L2/3_IS
L2/L3
L4
L5/L6
L5/L6-CC
C
Astrocyte
OL
OPC
COP
End-FLT
Microglia
End-LAMA
IN-SV2C
IN-VIP
IN-PV
IN-SST
L2/3_IS
L2/L3
L5/L6
L4
L5/L6-CC
R12121
R17043
Chimp2
Chimp3
1350
1385
1490
1452
1623
Bart
Frits
Ruben
D
CCA + louvain clustering
BBKNN + leiden clustering
Row fraction

Fig. S1. Species demultiplexing and data quality of a single cell transcriptome for adult and infant prefrontal cortex across primates. (**A**) Graphs showing species identity recovery per nucleus for each sequencing batch. Each sequencing batch contained a species pair as indicated by the colors, and all nuclei within each batch were aligned to both species' genomes indicated per axis. Species identity was assigned based on best fit, and all nuclei with <7% difference in genome alignments were removed. (**B**) Violin plots indicating number of PCR reduplicated reads (left plot) and number of unique genes detected (right plot) per nuclei, split per cell type indicated on the horizontal axis. (**C**) Correlation heatmaps post processing and dataset integration between cell types (annotation based on Seurat, left) and between individual samples (right). Color bar indicates Spearman correlation of global gene expression. (**D**) Heatmap displaying cell type annotation using either Seurat based preprocessing and integration (CCA integration followed by Louvain clustering; rows) versus Scanpy based preprocessing and integration (BBKNN integration and Leiden clustering; columns).

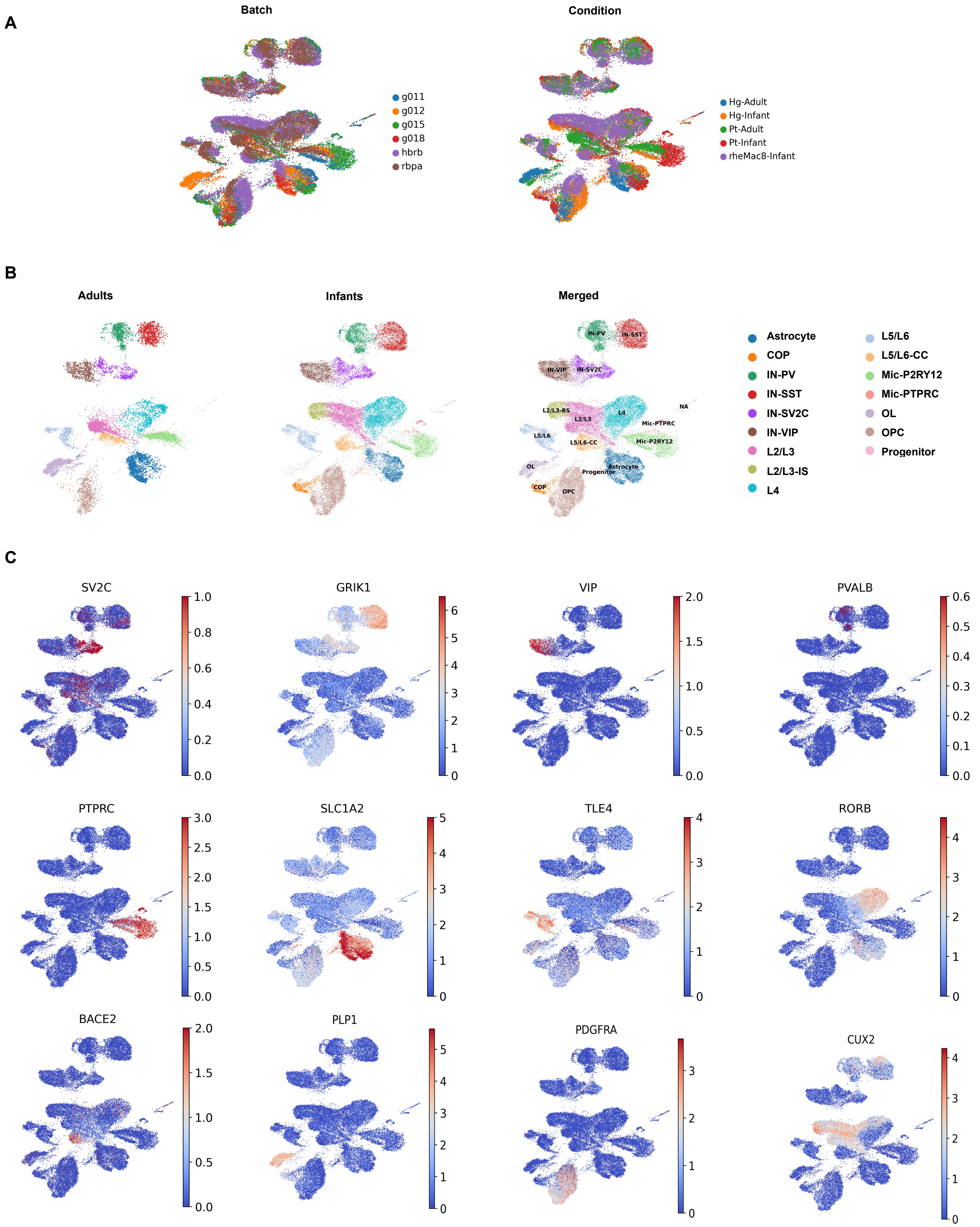
A
Batch
g011
g012
g015
g018
hbrb
rbpa
Condition
Hg-Adult
Hg-Infant
Pt-Adult
Pt-Infant
rheMac8-Infant
B
Adults
Infants
Merged
Astrocyte
COP
IN-PV
IN-SST
IN-SV2C
IN-VIP
L2/L3
L2/L3-IS
L4
L5/L6
L5/L6-CC
Mic-P2RY12
Mic-PTPRC
OL
OPC
Progenitor
C
SV2C
GRIK1
VIP
PVALB
PTPRC
SLC1A2
TLE4
RORB
BACE2
PLP1
PDGFRA
CUX2

Fig. S2. Data integration of a single cell transcriptomes for adult and infant prefrontal cortex across primates. (**A**) UMAP of all nuclei which passed final QC thresholds (n=37,994), colour maps indicate sequencing batch (left), age group (middle), and species /age (right). (**B**) UMAP visualization of infant (left) and adult (middle) splits of the data as shown in the right panel where colors match inferred cell types as indicated by colours. Arrowheads show infant and adult enriched clusters (**C**) Each panel contains a UMAP showing cell type representative markers not shown in Fig 1B. Colours indicate log2 of read density read per 10,000 unique transcripts.

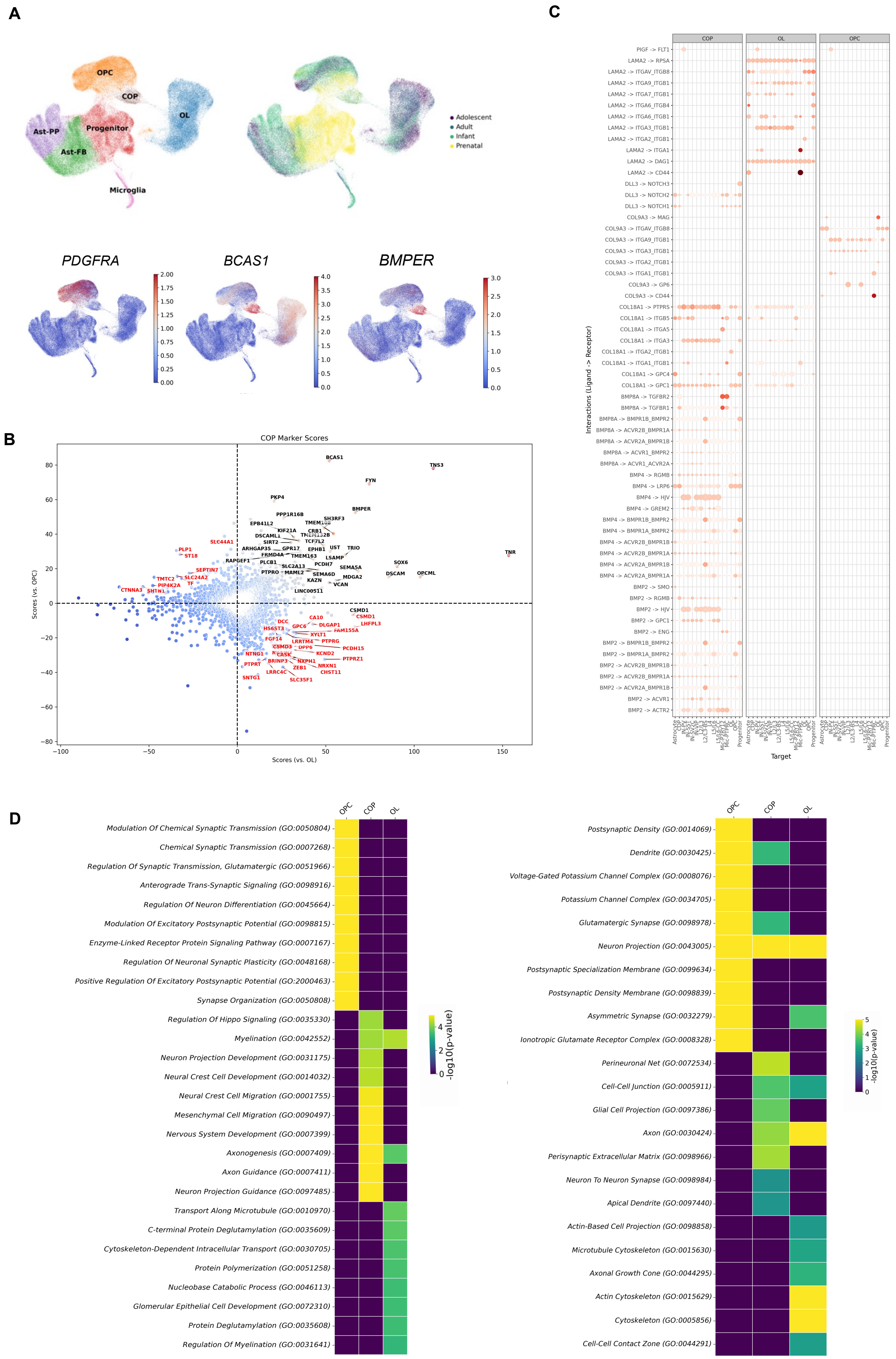
A
OPC
COP
OL
Ast-PP
Progenitor
Ast-FB
Microglia
Adolescent
Adult
Infant
Prenatal
PDGFRA
BCAS1
BMPER
B
COP Marker Scores
Scores (vs. OL)
Scores (vs. OPC)
BCAS1
TNS3
FYN
PKP4
BMPER
PPP1R16B
SH3RF3
EPB41L2
TMEM108
KIF21A
CRB1
DSCAML1
TMEM132B
SIRT2
TCF7L2
ARHGAP35
GPR17
EPHB1
UST
TRIO
FRMD4A
TMEM163
LSAMP
RAPGEF1
PLCB1
PCDH7
SOX6
SLC2A13
SEMA5A
PTPRO
MAML2
SEMA6D
DSCAM
OPCML
MDGA2
KAZN
VCAN
TNR
LINC00511
CSMD1
SLC44A1
PLP1
ST18
SEPTIN7
SLC24A2
TMTC2
PIP4K2A
TF
CTNNA3
SHTN1
CSMD1
LHFPL3
CA10
DLGAP1
DCC
GPC6
FAM155A
HS6ST3
XYLT1
FGF14
LRRTM4
PTPRG
CSMD3
DPP6
PCDH15
NTNG1
KCND2
CASK
PTPRZ1
BRINP3
NXPH1
PTPRT
ZEB1
NRXN1
LRRC4C
CHST11
SNTG1
SLC35F1
C
COP
OL
OPC
Interactions (Ligand -> Receptor)
Target
PIGF -> FLT1
LAMA2 -> RPSA
LAMA2 -> ITGAV_ITGB8
LAMA2 -> ITGA9_ITGB1
LAMA2 -> ITGA7_ITGB1
LAMA2 -> ITGA6_ITGB4
LAMA2 -> ITGA6_ITGB1
LAMA2 -> ITGA3_ITGB1
LAMA2 -> ITGA2_ITGB1
LAMA2 -> ITGA1
LAMA2 -> DAG1
LAMA2 -> CD44
DLL3 -> NOTCH3
DLL3 -> NOTCH2
DLL3 -> NOTCH1
COL9A3 -> MAG
COL9A3 -> ITGAV_ITGB8
COL9A3 -> ITGA9_ITGB1
COL9A3 -> ITGA3_ITGB1
COL9A3 -> ITGA2_ITGB1
COL9A3 -> ITGA1_ITGB1
COL9A3 -> GP6
COL9A3 -> CD44
COL18A1 -> PTPRS
COL18A1 -> ITGB5
COL18A1 -> ITGA5
COL18A1 -> ITGA3
COL18A1 -> ITGA2_ITGB1
COL18A1 -> ITGA1_ITGB1
COL18A1 -> GPC4
COL18A1 -> GPC1
BMP8A -> TGFBR2
BMP8A -> TGFBR1
BMP8A -> BMPR1B_BMPR2
BMP8A -> ACVR2B_BMPR1A
BMP8A -> ACVR2A_BMPR1B
BMP8A -> ACVR1_BMPR2
BMP8A -> ACVR1_ACVR2A
BMP4 -> RGMB
BMP4 -> LRP6
BMP4 -> HJV
BMP4 -> GREM2
BMP4 -> BMPR1B_BMPR2
BMP4 -> BMPR1A_BMPR2
BMP4 -> ACVR2B_BMPR1B
BMP4 -> ACVR2B_BMPR1A
BMP4 -> ACVR2A_BMPR1B
BMP4 -> ACVR2A_BMPR1A
BMP2 -> SMO
BMP2 -> RGMB
BMP2 -> HJV
BMP2 -> GPC1
BMP2 -> ENG
BMP2 -> BMPR1B_BMPR2
BMP2 -> BMPR1A_BMPR2
BMP2 -> ACVR2B_BMPR1B
BMP2 -> ACVR2B_BMPR1A
BMP2 -> ACVR2A_BMPR1B
BMP2 -> ACVR1
BMP2 -> ACTR2
Magnitude_rank
Specificity_rank
D
OPC
COP
OL
Modulation Of Chemical Synaptic Transmission (GO:0050804)
Chemical Synaptic Transmission (GO:0007268)
Regulation Of Synaptic Transmission, Glutamatergic (GO:0051966)
Anterograde Trans-Synaptic Signaling (GO:0098916)
Regulation Of Neuron Differentiation (GO:0045664)
Modulation Of Excitatory Postsynaptic Potential (GO:0098815)
Enzyme-Linked Receptor Protein Signaling Pathway (GO:0007167)
Regulation Of Neuronal Synaptic Plasticity (GO:0048168)
Positive Regulation Of Excitatory Postsynaptic Potential (GO:2000463)
Synapse Organization (GO:0050808)
Regulation Of Hippo Signaling (GO:0035330)
Myelination (GO:0042552)
Neuron Projection Development (GO:0031175)
Neural Crest Cell Development (GO:0014032)
Neural Crest Cell Migration (GO:0001755)
Mesenchymal Cell Migration (GO:0090497)
Nervous System Development (GO:0007399)
Axonogenesis (GO:0007409)
Axon Guidance (GO:0007411)
Neuron Projection Guidance (GO:0097485)
Transport Along Microtubule (GO:0010970)
C-terminal Protein Deglutamylation (GO:0035609)
Cytoskeleton-Dependent Intracellular Transport (GO:0030705)
Protein Polymerization (GO:0051258)
Nucleobase Catabolic Process (GO:0046113)
Glomerular Epithelial Cell Development (GO:0072310)
Protein Deglutamylation (GO:0035608)
Regulation Of Myelination (GO:0031641)
-log10(p-value)
Postsynaptic Density (GO:0014069)
Dendrite (GO:0030425)
Voltage-Gated Potassium Channel Complex (GO:0008076)
Potassium Channel Complex (GO:0034705)
Glutamatergic Synapse (GO:0098978)
Neuron Projection (GO:0043005)
Postsynaptic Specialization Membrane (GO:0099634)
Postsynaptic Density Membrane (GO:0098839)
Asymmetric Synapse (GO:0032279)
Ionotropic Glutamate Receptor Complex (GO:0008328)
Perineuronal Net (GO:0072534)
Cell-Cell Junction (GO:0005911)
Glial Cell Projection (GO:0097386)
Axon (GO:0030424)
Perisynaptic Extracellular Matrix (GO:0098966)
Neuron To Neuron Synapse (GO:0098984)
Apical Dendrite (GO:0097440)
Actin-Based Cell Projection (GO:0098858)
Microtubule Cytoskeleton (GO:0015630)
Axonal Growth Cone (GO:0044295)
Actin Cytoskeleton (GO:0015629)
Cytoskeleton (GO:0005856)
Cell-Cell Contact Zone (GO:0044291)
-log10(p-value)

Fig. S3. Developmental dynamics of glial cell types and infant enriched COPs. (**A**) (UMAP) showing Leiden clustering of additional PFC samples encompassing a span of developmental stages in humans (n= 171,175 glia nuclei; 106 individuals)(*24*). Clusters are labeled for corresponding study and samples by coloring as indicated. (**B**) Scatterplot displaying DE analysis results between COPs cells versus canonical oligodendrocytes (x-axis), and versus OPCs (y-axis). Positive markers for both comparisons are indicated in black text. (**C**) Dot plot visualization of a selection of top marker genes for all glial types for aggregated cluster data as indicates on top of the plot. Blue vs red coloring indicates log2 fold enrichment as shown in the side bar while the size of the dots represents the fraction of cells in the cluster expressing the indicated gene. (**D)** Gene Ontology analysis of positive markers for oligodendrocyte lineage cells obtained from (B), displaying top enriched terms (rows) for biological processes (left), and cellular component (right). Comparisons are depicted between oligodendrocyte subtypes (columns). Color bar indicates significance in -log10 scale (positive enrichment = yellow).

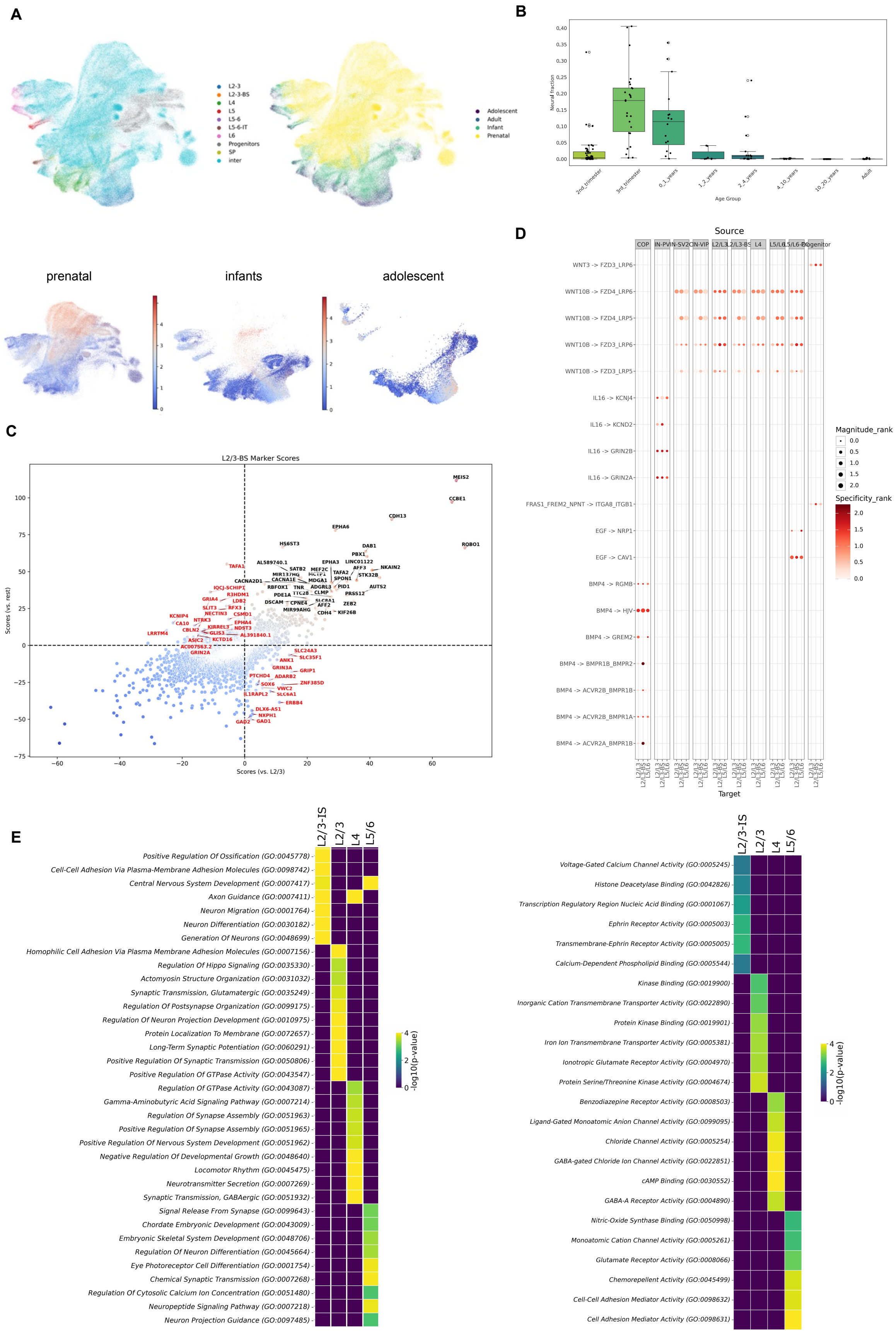

A
L2-3
L2-3-BS
L4
L5
L5-6
L5-6-IT
L6
Progenitors
SP
inter
Adolescent
Adult
Infant
Prenatal
prenatal
infants
adolescent
B
Neural fraction
2nd_trimester
3rd_trimester
0_1_years
1_2_years
2_4_years
4_10_years
10_20_years
Adult
Age Group
C
L2/3-BS Marker Scores
Scores (vs. rest)
Scores (vs. L2/3)
MEIS2
CCBE1
CDH13
EPHA6
ROBO1
HS6ST3
DAB1
PBX1
NKAIN2
SATB2
AFF3
STK32B
AUTS2
TAFA1
TAFA2
SPON1
ZEB2
KIF26B
CDH4
CLMP
TNR
PDE1A
DSCAM
CPNE4
MIR99AHG
IQCJ-SCHIP1
R3HDM1
GRIA4
LDB2
SLIT3
RFX3
NECTIN3
CSMD1
KCNIP4
CA10
NTRK3
EPHA4
KIRREL3
NDST3
LRRTM4
CBLN2
GLIS3
AL391840.1
ASIC2
KCTD16
AC007563.2
GRIN2A
SLC24A3
SLC35F1
ANK1
GRIN3A
GRIP1
PTCHD4
ADARB2
ZNF385D
SOX6
VWC2
IL1RAPL2
SLC6A1
ERBB4
DLX6-AS1
NXPH1
GAD2
GAD1
D
Source
COP
IN-PV
IN-SV2C
IN-VIP
L2/L3
L2/L3-BS
L4
L5/L6
L5/L6-BS
Progenitor
WNT3 -> FZD3_LRP6
WNT10B -> FZD4_LRP6
WNT10B -> FZD4_LRP5
WNT10B -> FZD3_LRP6
WNT10B -> FZD3_LRP5
IL16 -> KCNJ4
IL16 -> KCND2
IL16 -> GRIN2B
IL16 -> GRIN2A
FRAS1_FREM2_NPNT -> ITGA8_ITGB1
EGF -> NRP1
EGF -> CAV1
BMP4 -> RGMB
BMP4 -> HJV
BMP4 -> GREM2
BMP4 -> BMPR1B_BMPR2
BMP4 -> ACVR2B_BMPR1B
BMP4 -> ACVR2B_BMPR1A
BMP4 -> ACVR2A_BMPR1B
Magnitude_rank
Specificity_rank
Target
E
L2/3-IS
L2/3
L4
L5/6
-log10(p-value)
Positive Regulation Of Ossification (GO:0045778)
Cell-Cell Adhesion Via Plasma-Membrane Adhesion Molecules (GO:0098742)
Central Nervous System Development (GO:0007417)
Axon Guidance (GO:0007411)
Neuron Migration (GO:0001764)
Neuron Differentiation (GO:0030182)
Generation Of Neurons (GO:0048699)
Homophilic Cell Adhesion Via Plasma Membrane Adhesion Molecules (GO:0007156)
Regulation Of Hippo Signaling (GO:0035330)
Actomyosin Structure Organization (GO:0031032)
Synaptic Transmission, Glutamatergic (GO:0035249)
Regulation Of Postsynapse Organization (GO:0099175)
Regulation Of Neuron Projection Development (GO:0010975)
Protein Localization To Membrane (GO:0072657)
Long-Term Synaptic Potentiation (GO:0060291)
Positive Regulation Of Synaptic Transmission (GO:0050806)
Positive Regulation Of GTPase Activity (GO:0043547)
Regulation Of GTPase Activity (GO:0043087)
Gamma-Aminobutyric Acid Signaling Pathway (GO:0007214)
Regulation Of Synapse Assembly (GO:0051963)
Positive Regulation Of Synapse Assembly (GO:0051965)
Positive Regulation Of Nervous System Development (GO:0051962)
Negative Regulation Of Developmental Growth (GO:0048640)
Locomotor Rhythm (GO:0045475)
Neurotransmitter Secretion (GO:0007269)
Synaptic Transmission, GABAergic (GO:0051932)
Signal Release From Synapse (GO:0099643)
Chordate Embryonic Development (GO:0043009)
Embryonic Skeletal System Development (GO:0048706)
Regulation Of Neuron Differentiation (GO:0045664)
Eye Photoreceptor Cell Differentiation (GO:0001754)
Chemical Synaptic Transmission (GO:0007268)
Regulation Of Cytosolic Calcium Ion Concentration (GO:0051480)
Neuropeptide Signaling Pathway (GO:0007218)
Neuron Projection Guidance (GO:0097485)
Voltage-Gated Calcium Channel Activity (GO:0005245)
Histone Deacetylase Binding (GO:0042826)
Transcription Regulatory Region Nucleic Acid Binding (GO:0001067)
Ephrin Receptor Activity (GO:0005003)
Transmembrane-Ephrin Receptor Activity (GO:0005005)
Calcium-Dependent Phospholipid Binding (GO:0005544)
Kinase Binding (GO:0019900)
Inorganic Cation Transmembrane Transporter Activity (GO:0022890)
Protein Kinase Binding (GO:0019901)
Iron Ion Transmembrane Transporter Activity (GO:0005381)
Ionotropic Glutamate Receptor Activity (GO:0004970)
Protein Serine/Threonine Kinase Activity (GO:0004674)
Benzodiazepine Receptor Activity (GO:0008503)
Ligand-Gated Monoatomic Anion Channel Activity (GO:0099095)
Chloride Channel Activity (GO:0005254)
GABA-gated Chloride Ion Channel Activity (GO:0022851)
cAMP Binding (GO:0030552)
GABA-A Receptor Activity (GO:0004890)
Nitric-Oxide Synthase Binding (GO:0050998)
Monoatomic Cation Channel Activity (GO:0005261)
Glutamate Receptor Activity (GO:0008066)
Chemorepellent Activity (GO:0045499)
Cell-Cell Adhesion Mediator Activity (GO:0098632)
Cell Adhesion Mediator Activity (GO:0098631)

Fig. S4. Developmental dynamics of infant enriched L2/L3 (L2L3-IS) projection neurons. (**A**) UMAPs showing Leiden clustering of additional PFC samples encompassing a span of developmental stages in humans (n= 374,463 neuronal nuclei; 106 individuals)(*24*). Clusters are labeled for corresponding cell types based on marker gene expression (left) and age group (right) by coloring as indicated. Bottom three UMAPs show module score expression for the L2/3-IS marker gene sets (obtained in Fig. S4C) for prenatal (left), infant (middle) postnatal samples (right). Colours indicate read density read per 10k unique transcripts. (**B**) Quantification of L2L3-IS cell type proportion across age groups. Each data point is calculated per individual, and for visualization grouped into age ranges. Boxplot coloring indicates age range as shown in (A). (**C**) Scatterplot displaying DEA results between L2/3_IS cells versus canonical L2/3 neurons (x-axis), and versus all other neurons (y-axis). Positive markers for both comparisons are indicated in black text. (**D**) Cell-cell communication modeling using LIgand-receptor ANalysis frAmework (LIANA). Each row indicates a unique ligand receptor (LR) interaction. Each separate panel indicates source (sender) cell type as indicated on top, whereas sub columns indicate target (receiver) cell type listed at the bottom. Examples shown are LRs unique to each cell type. Dot color indicates interaction specificity scores, and dot size indicates magnitude scores. (**E**) Gene Ontology analysis of L2/3_IS positive markers obtained from (C), displaying top enriched terms (rows) for biological processes (left), molecular function (middle), and cellular component (right). Comparisons are depicted between other excitatory neuron subtypes (columns). Color bar indicates significance in -log10 scale (positive enrichment = yellow).

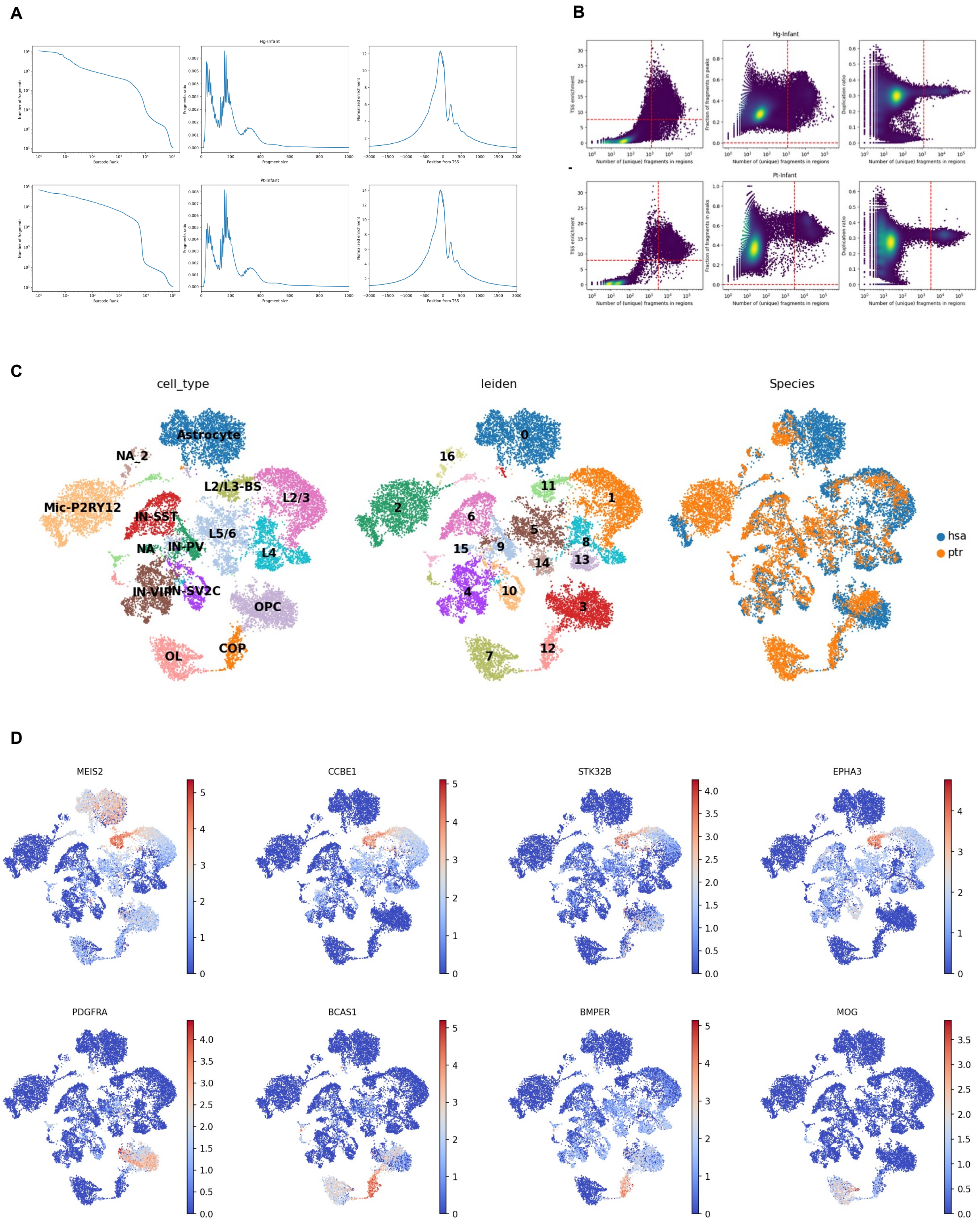

A
Hg-Infant
Pt-Infant
Barcode Rank
Number of fragments
Fragment size
Fragments ratio
Position from TSS
Normalized enrichment
B
Hg-Infant
Pt-Infant
TSS enrichment
Fraction of fragments in peaks
Duplication ratio
Number of (unique) fragments in regions
C
cell_type
leiden
Species
Astrocyte
NA_2
L2/L3-BS
L2/3
Mic-P2RY12
IN-SST
L5/6
NA
IN-PV
L4
IN-VIP
IN-SV2C
OPC
COP
OL
hsa
ptr
D
MEIS2
CCBE1
STK32B
EPHA3
PDGFRA
BCAS1
BMPER
MOG

Fig. S5. Multi-omic characterization of human and chimpanzee infant PFC. (**A**) Density plots displaying number of fragments per nuclei (left), fragment sizes (middle), and position relative to all TSS (right) for snATACseq library in human infant (top panel) versus chimpanzee infant (bottom panel). (**B**) Quality control scatterplots with y-axis displaying TSS enrichment (left), fraction of reads in peak (middle), and PCR duplicates (right) versus number of unique snATAC fragments per nuclei (x-axis) in human infant (top panel) versus chimpanzee infant (bottom panel). Red dashed lines indicate thresholds determined by SCENIC+ based on density separation. (**C**) UMAP showing Leiden clustering (middle panel) for the multiome datasets. Species distribution is shown in the right panel while maker-based cell type assignments are shown in the left panel. (**D**) UMAPs showing marker gene expression with each marker gene displayed at the top, for cell type assignments shown in (C). Colours indicate read density read per 10k normalized.

A

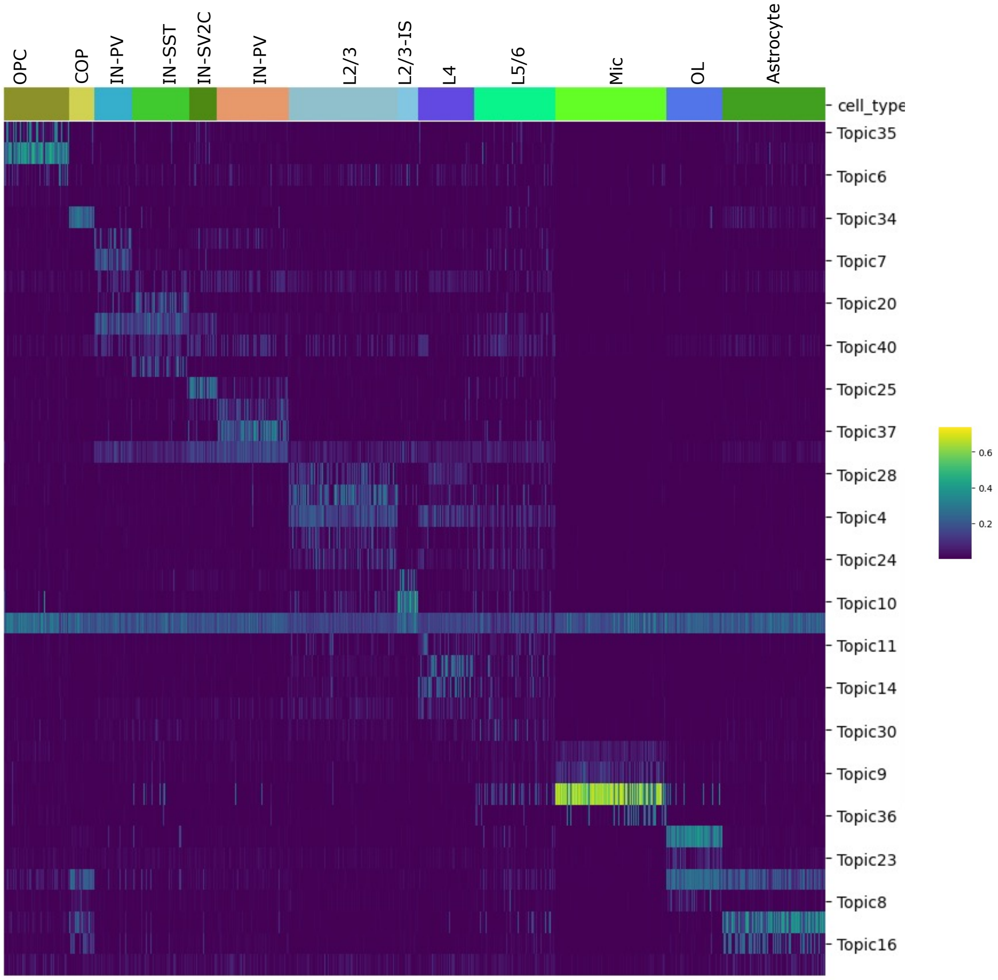
OPC
COP
IN-PV
IN-SST
IN-SV2C
IN-PV
L2/3
L2/3-IS
L4
L5/6
Mic
OL
Astrocyte
cell_type
Topic35
Topic6
Topic34
Topic7
Topic20
Topic40
Topic25
Topic37
Topic28
Topic4
Topic24
Topic10
Topic11
Topic14
Topic30
Topic9
Topic36
Topic23
Topic8
Topic16
0.6
0.4
0.2

B

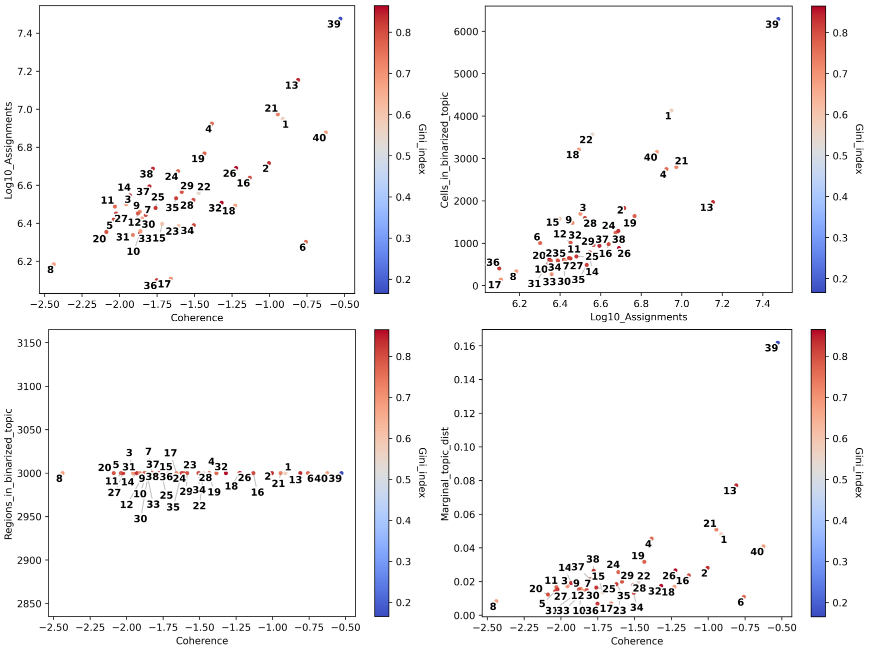
Log10_Assignments
Coherence
Gini_index
Cells_in_binarized_topic
Log10_Assignments
Gini_index
Regions_in_binarized_topic
Coherence
Gini_index
Marginal_topic_dist
Coherence
Gini_index

Fig. S6. **Regulome assignment in human and chimpanzee infant PFC.** (**A**) Heatmap of topics (rows) across cells (columns) following latent drichlet allocation (LDA) topic modelling using pyCisTopic. Color bar indicates normalized score contribution [0,1] per given topic to a cell's snATAC profile. Heatmap indicates all topics from our 40-topic model with no pre-selection for visualization. (**B**) Quality control analyses for identified snATAC topics, quantifying topic coherence (strength of co-accessibility in topics' regions) versus number of assigned regions per topic (top left), number of cells assigned to each topic following cell-topic binarization (top right), number of regions per topic versus coherence (bottom left), and marginal topic distribution (i.e. a given topic's contribution to the overall model) (bottom right). All points are further colored based on Gini index of this topic [0 = nonspecific, 1 = highly specific].

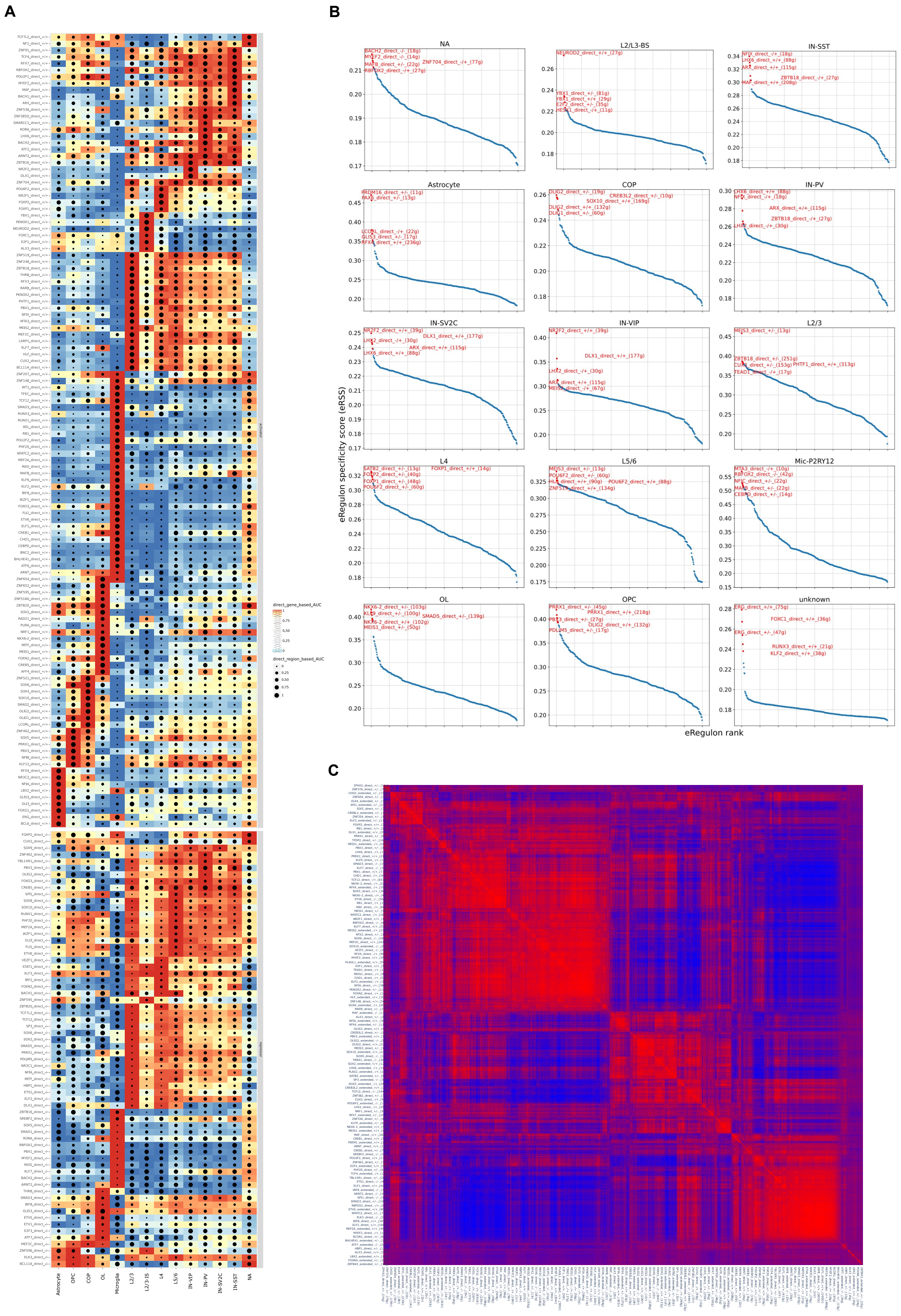
A
B
C
NA
BACH2_direct_-/-_(18g)
MYEF2_direct_-/-_(14g)
MAFB_direct_+/-_(22g)
ZNF704_direct_-/+_(77g)
RBFOX2_direct_-/+_(27g)
L2/L3-BS
NEUROD2_direct_+/+_(27g)
YBX1_direct_+/-_(81g)
YBX1_direct_+/+_(29g)
E2F2_direct_+/-_(35g)
HES1_direct_-/+_(11g)
IN-SST
NFIX_direct_-/+_(18g)
LHX6_direct_+/+_(88g)
ARX_direct_+/+_(115g)
ZBTB18_direct_-/+_(27g)
MAF_direct_+/+_(208g)
Astrocyte
PRDM16_direct_+/-_(11g)
PAX6_direct_+/-_(13g)
LCOR_direct_-/+_(22g)
GLIS3_direct_+/-_(17g)
RFX4_direct_+/+_(236g)
COP
OLIG2_direct_+/-_(19g)
CREB3L2_direct_+/-_(10g)
SOX10_direct_+/+_(169g)
OLIG2_direct_+/+_(132g)
OLIG1_direct_+/-_(60g)
IN-PV
LHX6_direct_+/+_(88g)
NFIX_direct_-/+_(18g)
ARX_direct_+/+_(115g)
ZBTB18_direct_-/+_(27g)
LHX2_direct_-/+_(30g)
IN-SV2C
NR2F2_direct_+/+_(39g)
DLX1_direct_+/+_(177g)
LHX2_direct_-/+_(30g)
ARX_direct_+/+_(115g)
LHX6_direct_+/+_(88g)
IN-VIP
NR2F2_direct_+/+_(39g)
DLX1_direct_+/+_(177g)
LHX2_direct_-/+_(30g)
ARX_direct_+/+_(115g)
MEIS2_direct_-/+_(67g)
L2/3
MEIS3_direct_+/-_(13g)
ZBTB18_direct_+/-_(251g)
CUX1_direct_+/-_(153g)
PHTF1_direct_+/+_(313g)
TEAD1_direct_-/+_(17g)
L4
SATB2_direct_+/-_(13g)
FOXP1_direct_+/+_(14g)
FOXP2_direct_+/-_(40g)
FOXP1_direct_+/-_(48g)
POU6F2_direct_+/-_(60g)
L5/6
MEIS3_direct_+/-_(13g)
POU6F2_direct_+/-_(60g)
HLF_direct_+/+_(90g)
POU6F2_direct_+/+_(88g)
ZNF519_direct_+/+_(134g)
Mic-P2RY12
MTA3_direct_-/+_(10g)
RBFOX2_direct_-/-_(42g)
NFIC_direct_+/-_(22g)
MAFB_direct_+/-_(22g)
CEBPD_direct_+/-_(14g)
OL
NKX6-2_direct_+/-_(103g)
KLF9_direct_+/-_(100g)
SMAD5_direct_+/-_(139g)
NKX6-2_direct_+/+_(102g)
MEIS1_direct_+/-_(50g)
OPC
PRRX1_direct_+/-_(45g)
PRRX1_direct_+/+_(218g)
PBX3_direct_+/-_(27g)
OLIG2_direct_+/+_(132g)
POLM5_direct_+/-_(17g)
unknown
ERG_direct_+/+_(75g)
FOXC1_direct_+/+_(36g)
ERG_direct_+/-_(47g)
RUNX3_direct_+/+_(21g)
KLF2_direct_+/+_(38g)
eRegulon specificity score (eRSS)
eRegulon rank
direct_gene_based_AUC
direct_region_based_AUC
Astrocyte
OPC
COP
OL
Microglia
L2/3
L2/3-IS
L4
L5/6
IN-VIP
IN-PV
IN-SV2C
IN-SST
NA

Fig. S7. Network analysis multi-omic data in human and chimpanzee infant PFC. (**A**) SCENIC+ regulon heatmap dot plot for combined human and chimpanzee infant PFC data with each row representing a unique regulon and each column indicating a cell type's average profile across regulons. Color bar indicates TF-to-gene Area Under the Curve (AUC) ranging from blue (nonspecific) to red (highly specific). Dot size indicates Region-to-gene AUC (larger dots = more specific). Top part of the heatmap contains activator regulons, while the lower part shows repressor regulons. (**B**) Regulon specificity scores (RSS) for each cell type ranked in descending order of specificity. Top 5 most specific regulons per cell type with their driver transcription factors are highlighted in red text. (**C**) Correlation heatmap between all high-quality regulons (n= 186) ranging from dark blue (no correlation) to red (high correlation).

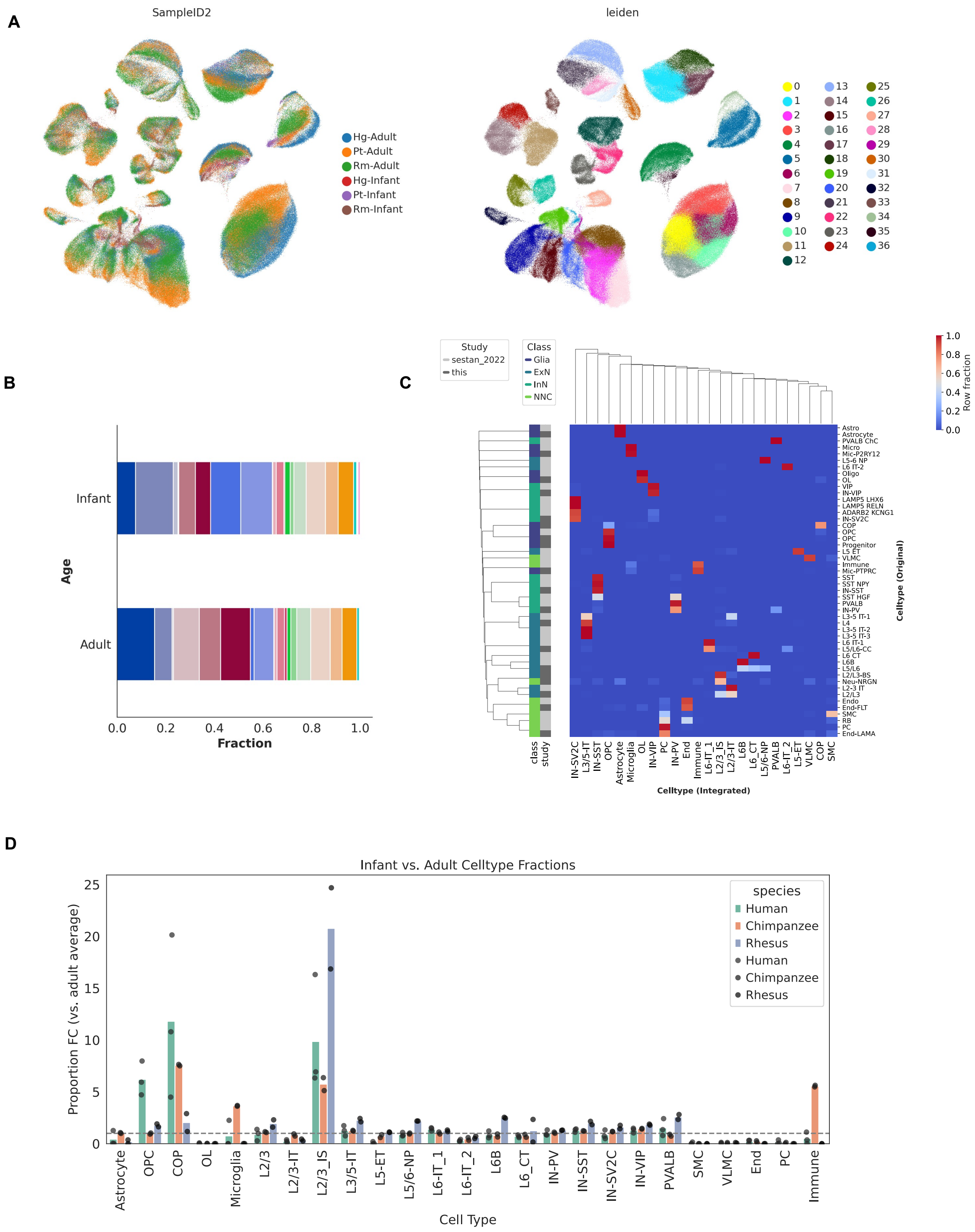
A
SampleID2
Hg-Adult
Pt-Adult
Rm-Adult
Hg-Infant
Pt-Infant
Rm-Infant
leiden
0 1 2 3 4 5 6 7 8 9 10 11 12 13 14 15 16 17 18 19 20 21 22 23 24 25 26 27 28 29 30 31 32 33 34 35 36
B
Infant
Adult
Age
0.0 0.2 0.4 0.6 0.8 1.0
Fraction
C
Study
sestan_2022
this
Class
Glia
ExN
InN
NNC
1.0 0.8 0.6 0.4 0.2 0.0
Row fraction
Astro
Astrocyte
PVALB ChC
Micro
Mic-P2RY12
L5-6 NP
L6 IT-2
Oligo
OL
VIP
IN-VIP
LAMP5 LHX6
LAMP5 RELN
ADARB2 KCNG1
IN-SV2C
COP
OPC
OPC
Progenitor
L5 ET
VLMC
Immune
Mic-PTPRC
SST
SST NPY
IN-SST
SST HGF
PVALB
IN-PV
L3-5 IT-1
L4
L3-5 IT-2
L3-5 IT-3
L6 IT-1
L5/L6-CC
L6 CT
L6B
L5/L6
L2/L3-BS
Neu-NRGN
L2-3 IT
L2/L3
Endo
End-FLT
SMC
RB
PC
End-LAMA
Celltype (Original)
class
study
IN-SV2C
L3/5-IT
IN-SST
OPC
Astrocyte
Microglia
OL
IN-VIP
PC
IN-PV
End
Immune
L6-IT_1
L2/3_IS
L2/3-IT
L6B
L6_CT
L5/6-NP
PVALB
L6-IT_2
L5-ET
VLMC
COP
SMC
Celltype (Integrated)
D
Infant vs. Adult Celltype Fractions
species
Human
Chimpanzee
Rhesus
Human
Chimpanzee
Rhesus
Proportion FC (vs. adult average)
0 5 10 15 20 25
Astrocyte
OPC
COP
OL
Microglia
L2/3
L2/3-IT
L2/3_IS
L3/5-IT
L5-ET
L5/6-NP
L6-IT_1
L6-IT_2
L6B
L6_CT
IN-PV
IN-SST
IN-SV2C
IN-VIP
PVALB
SMC
VLMC
End
PC
Immune
Cell Type

Fig. S8. Integration of adult human, chimpanzee and rhesus macaque data. (**A**) UMAP showing Leiden clustering of BBKNN integrated data comprising of nuclei for infant (n=37,994) and adult human chimpanzee and rhesus macaque PFC samples(*26*) totaling 499,245 nuclei. Left panel shows species integration and right panel shows Leiden clusters as indicated by colours. (**B**) Compositional characterization of infant and adult primate PFC across species. Nuclei were aggregated per age group and proportions of each cell type are quantified as indicated by colors. (**C**) label transfer heatmap between infant and more densely populated adult datasets (rows) showing newly integrated clustering labels (columns). Colorbar indicates proportion of this row's observations in a given cell type. (**D**) Barplot showing quantification of infant vs. adult cell type proportions using the additional adult datasets as a reference. Each dot represents an individual, and bars indicate proportion FC infant vs adult average.

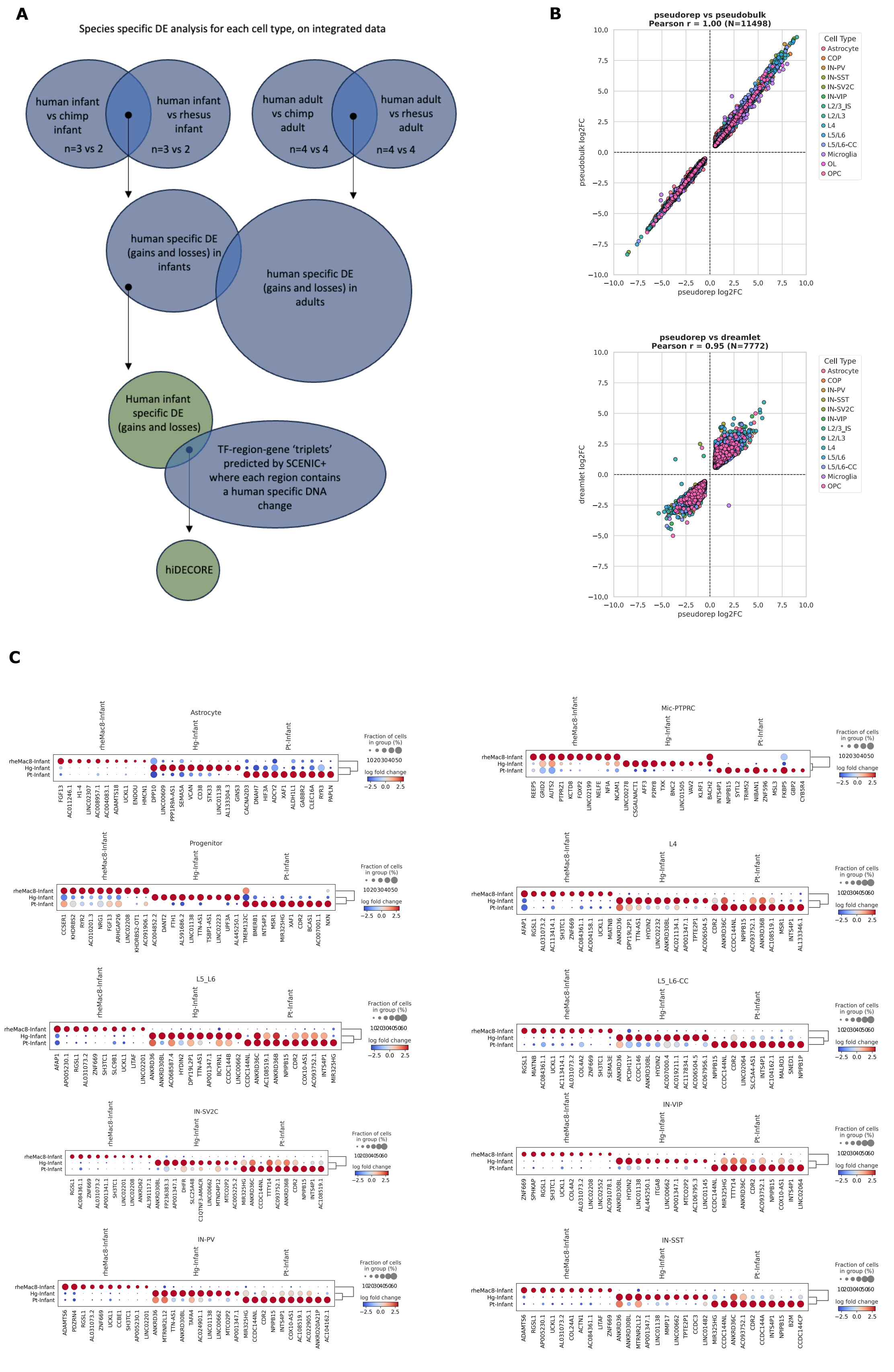

A
Species specific DE analysis for each cell type, on integrated data
human infant vs chimp infant
n=3 vs 2
human infant vs rhesus infant
n=3 vs 2
human adult vs chimp adult
n=4 vs 4
human adult vs rhesus adult
n=4 vs 4
human specific DE (gains and losses) in infants
human specific DE (gains and losses) in adults
Human infant specific DE (gains and losses)
TF-region-gene 'triplets' predicted by SCENIC+ where each region contains a human specific DNA change
hiDECORE
B
pseudorep vs pseudobulk
Pearson r = 1.00 (N=11498)
pseudobulk log2FC
pseudorep log2FC
Cell Type
Astrocyte
COP
IN-PV
IN-SST
IN-SV2C
IN-VIP
L2/3_IS
L2/L3
L4
L5/L6
L5/L6-CC
Microglia
OL
OPC
pseudorep vs dreamlet
Pearson r = 0.95 (N=7772)
dreamlet log2FC
pseudorep log2FC
Cell Type
Astrocyte
COP
IN-PV
IN-SST
IN-SV2C
IN-VIP
L2/3_IS
L2/L3
L4
L5/L6
L5/L6-CC
Microglia
OPC
C
Astrocyte
Mic-PTPRC
Progenitor
L4
L5_L6
L5_L6-CC
IN-SV2C
IN-VIP
IN-PV
IN-SST
rheMac8-Infant
Hg-Infant
Pt-Infant
Fraction of cells in group (%)
log fold change

Fig. S9. Differential gene expression analysis of infant human, chimpanzee and rhesus macaque data. (A) DEA strategy of evolutionary comparisons between primate infants, and comparisons between primate adults. (**B)** Comparison of the main DEA findings versus a standard pseudobulk approach using pyDESEQ (top), and versus dreamlet pseudobulk approach (bottom). Indicated are effect sizes (log2FC in human infants versus chimpanzee and rhesus infants) of overlapped DE genes. Headers indicate how many of the original DEA findings (n=12,908 genes) are captured by each approach, along with correlations of effect sizes. (**C**) Dot plots complementary to Fig. 2D displayed per cell type as indicated on the top of each plot, showing examples of top DE genes between species in infant PFC for human (Top), chimpanzee (middle) and rhesus macaque (Bottom). Dot sizes indicate percentage of cells expressing the indicated gene and colors show average log2 fold change (FC) as shown in the sidebars.

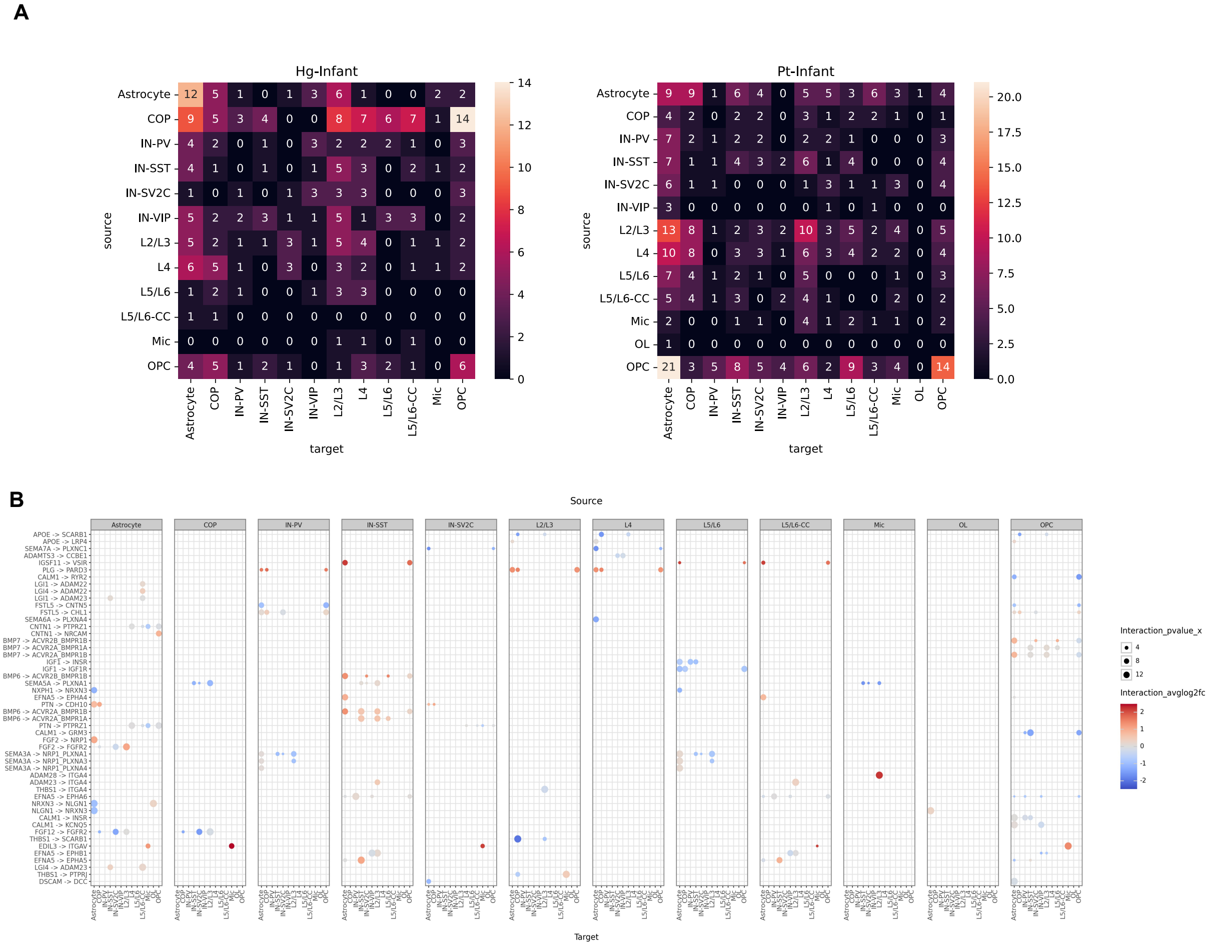
A
Hg-Infant
Pt-Infant
source
target
Astrocyte
COP
IN-PV
IN-SST
IN-SV2C
IN-VIP
L2/L3
L4
L5/L6
L5/L6-CC
Mic
OL
OPC
B
Source
Target
Interactions (Ligand -> Receptor)
Interaction_pvalue_x
Interaction_avglog2fc

Fig. S10. Cell-cell communication modeling of infant human specific gene expression changes compared to chimpanzee and rhesus macaque data in PFC. (A) Context-specific modelling of Cell-cell communication modeling using LIgand-receptor ANalysis frAmework (LIANA+). Sender-receiver matrix of significant (specificity rank < 0.05, magnitude rank < 0.05) ligand-receptor (LR) interactions for this given condition given its DEA profile showing; human infant enriched (left), and chimpanzee infant enriched (right), interactions. Senders are on the y-axis while receivers are on the x-axis. Colors indicate number of interactions per cell type. (**B**) Dot plot visualization of top 50 LRs as in main Fig. 3F. but for chimpanzee infant (top). Each row indicates a unique ligand receptor (LR) interaction. Each separate panel indicates source (sender) cell type as indicated on top, whereas sub columns indicate target (receiver) cell type listed at the bottom. Dot color indicates interaction specificity scores, and dot size indicates magnitude scores.

A

B

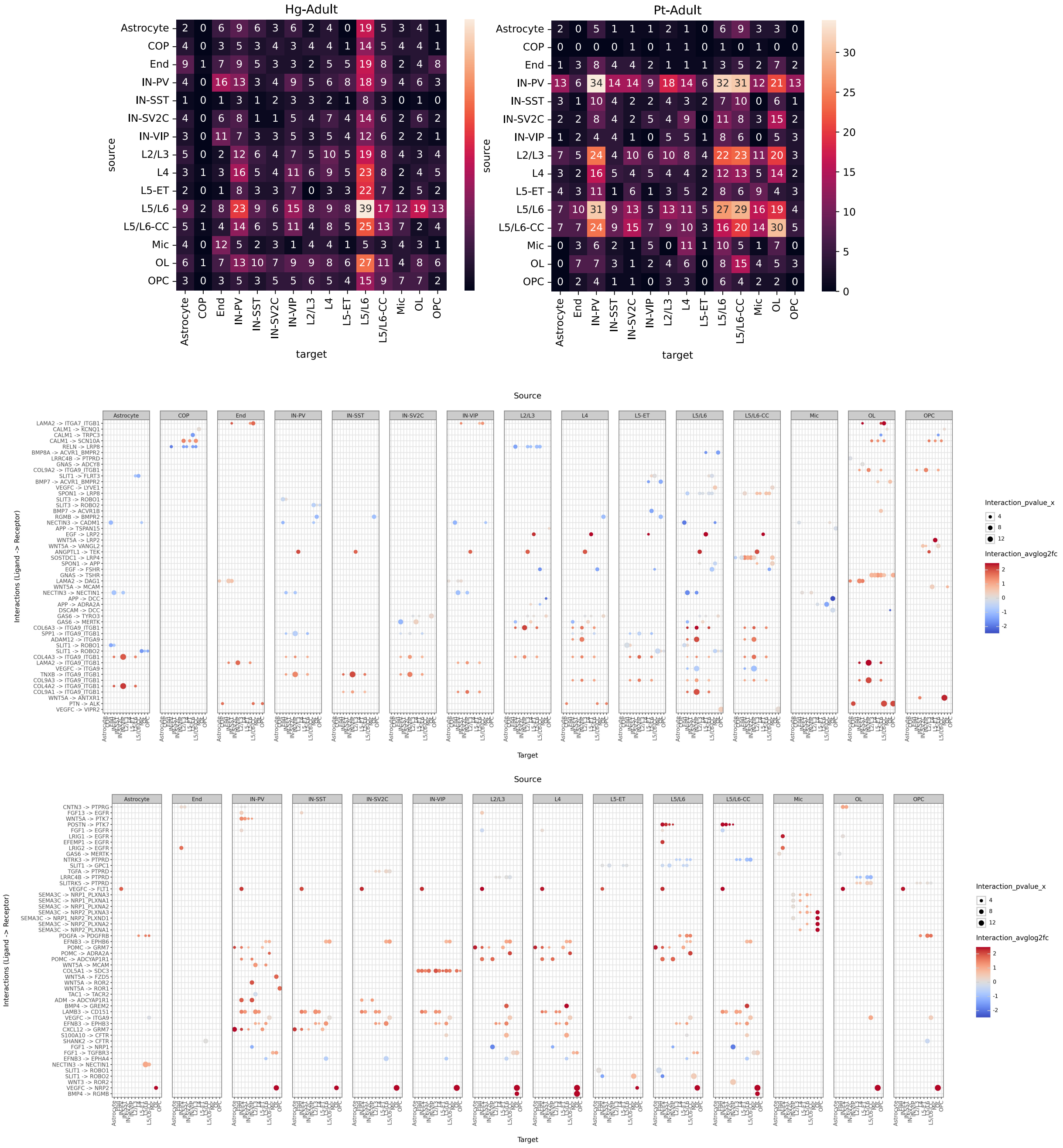

Hg-Adult
Pt-Adult
source
target
Astrocyte
COP
End
IN-PV
IN-SST
IN-SV2C
IN-VIP
L2/L3
L4
L5-ET
L5/L6
L5/L6-CC
Mic
OL
OPC
Source
Target
Interactions (Ligand -> Receptor)
Interaction_pvalue_x
Interaction_avglog2fc

Fig. S11. **Adult cell-cell communication modeling of human specific gene expression changes compared to chimpanzee and rhesus macaque data in PFC. (A)** Context-specific modelling of Cell-cell communication modeling using LIANA+. Sender-receiver matrix of significant (specificity rank < 0.05, magnitude rank < 0.05) ligand-receptor (LR) interactions for this given condition given its DEA profile showing; human adult enriched (left), and chimpanzee adult enriched (right) interactions. Senders are on the y-axis while receivers are on the x-axis. Colors indicate number of interactions per cell type. (**B**) Dot plot visualization of top 50 LRs as in main Fig. 3F. but for human adult (top) and chimpanzee adult (bottom). Each row indicates a unique ligand receptor (LR) interaction. Each separate panel indicates source (sender) cell type as indicated on top, whereas sub columns indicate target (receiver) cell type listed at the bottom. Dot color indicates interaction specificity scores, and dot size indicates magnitude scores.

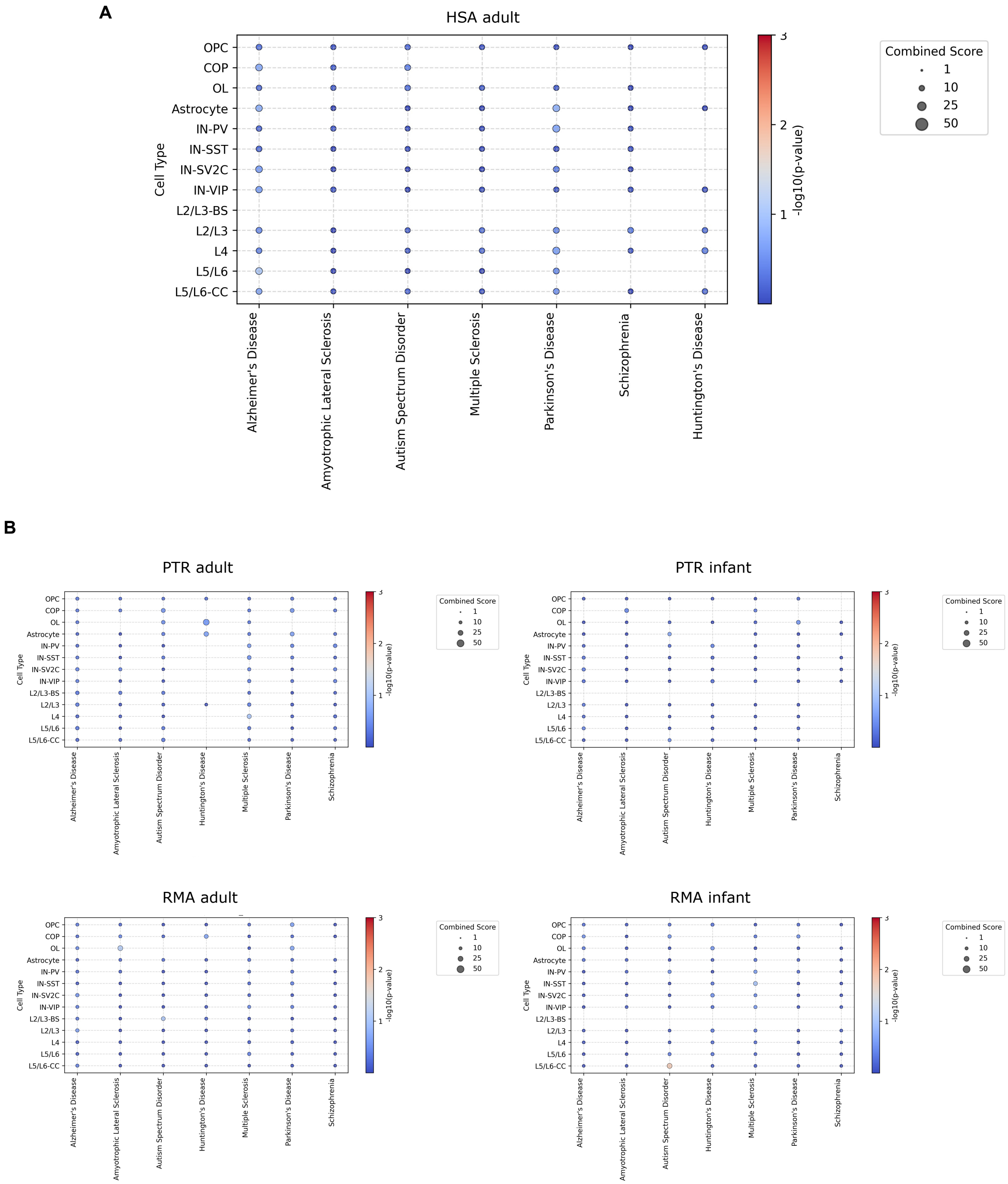

A
HSA adult
OPC
COP
OL
Astrocyte
IN-PV
IN-SST
IN-SV2C
IN-VIP
L2/L3-BS
L2/L3
L4
L5/L6
L5/L6-CC
Cell Type
Alzheimer's Disease
Amyotrophic Lateral Sclerosis
Autism Spectrum Disorder
Multiple Sclerosis
Parkinson's Disease
Schizophrenia
Huntington's Disease
-log10(p-value)
1
2
3
Combined Score
1
10
25
50
B
PTR adult
PTR infant
RMA adult
RMA infant
Huntington's Disease

Fig. S12. Interactions of disease risk genes with gene expression changes specific to human, chimpanzee and rhesus macaque, infants and adults in PFC. (A) Dot plot per cell type DE gains (rows) across representative neural diseases as represented by curated risk genes for each disease(*47*). Dot size indicates the magnitude of EnrichR overrepresentation score, and color bar indicates statistical significance (hypergeometric test with FDR correction). Data is shown for human adults. Panels are separated per each species set of DE gains. (**B**) Same as (A) but for chimpanzee infants (top left), chimpanzee adults (top right), rhesus infants (bottom left), rhesus adults (bottom right).

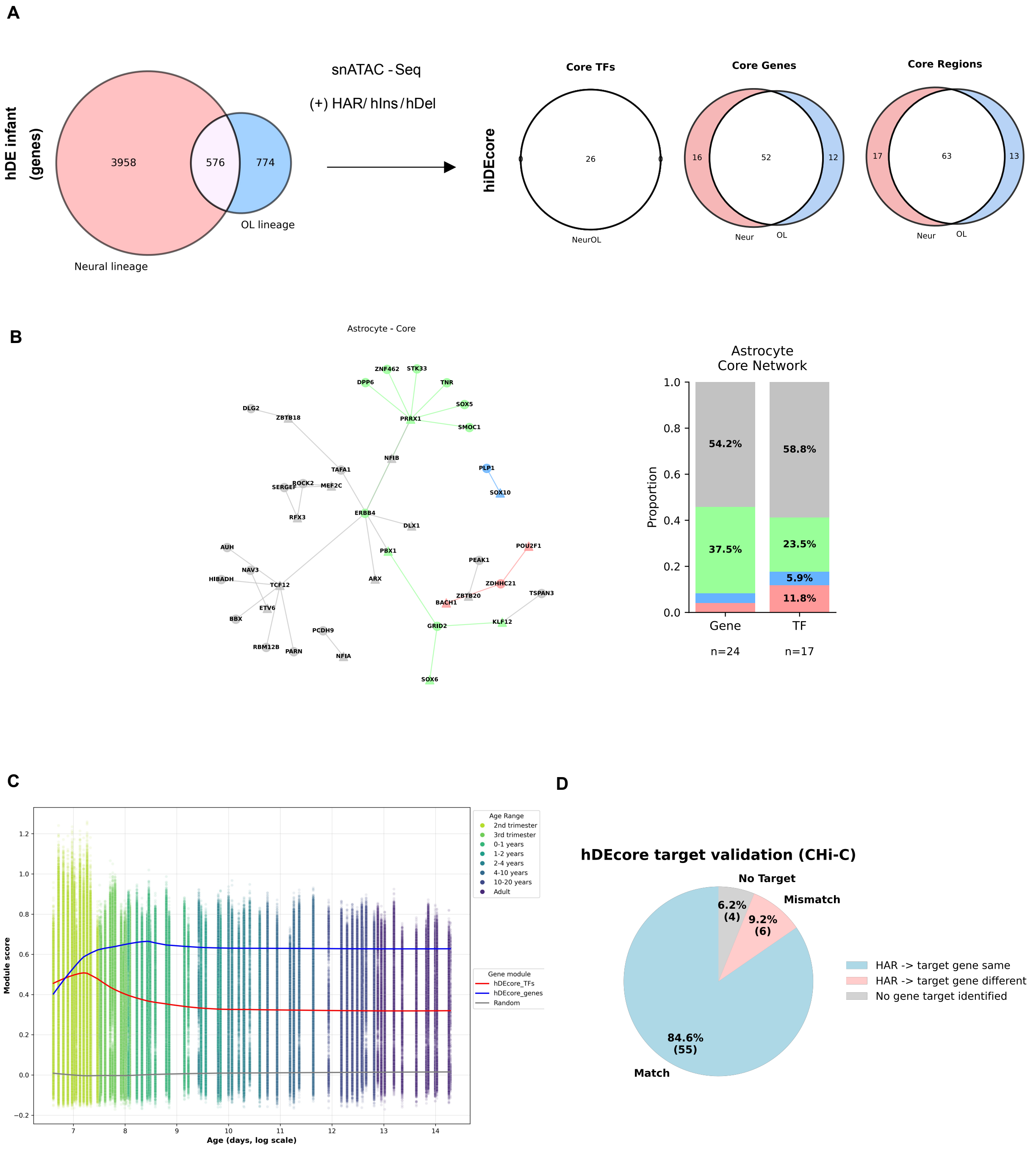
A
hDE infant (genes)
3958
576
774
Neural lineage
OL lineage
snATAC -Seq
(+) HAR/ hIns / hDel
hiDEcore
Core TFs
26
NeurOL
Core Genes
16
52
12
Neur
OL
Core Regions
17
63
13
Neur
OL
B
Astrocyte - Core
ZNF462
STK33
DPP6
TNR
SOX5
DLG2
ZBTB18
PRRX1
SMOC1
NFIB
TAFA1
PLP1
SERGEF
ROCK2
MEF2C
SOX10
RFX3
ERBB4
DLX1
AUH
PBX1
POU2F1
PEAK1
NAV3
HIBADH
TCF12
ARX
ZDHHC21
ZBTB20
TSPAN3
BACH1
ETV6
BBX
PCDH9
GRID2
KLF12
RBM12B
PARN
NFIA
SOX6
Astrocyte Core Network
Proportion
54.2%
58.8%
37.5%
23.5%
5.9%
11.8%
Gene
TF
n=24
n=17
C
Age Range
2nd trimester
3rd trimester
0-1 years
1-2 years
2-4 years
4-10 years
10-20 years
Adult
Gene module
hDEcore_TFs
hDEcore_genes
Random
Module score
Age (days, log scale)
D
hDEcore target validation (CHi-C)
No Target
Mismatch
Match
6.2% (4)
9.2% (6)
84.6% (55)
HAR -> target gene same
HAR -> target gene different
No gene target identified

Fig. S13. Multiomic gene regulatory network modeling of human infant-specific DEA genes linked to ATAC peaks with human-specific accelerated regions insertions/deletions (hDECORE). (A) Generation of hDECORE network derived from human infant DE genes (left venn), analyzing the subset of gains which are positively linked to putative enhancers containing human-specific genomic substitutions/base changes (right venns). Venn diagram each visualize the intersection of genes, TFs, and regions between neural lineage cells (red venns) versus oligodendrocyte lineage cells (blue venns). **(B)** Network visualization of hDECORE in astrocyte lineage, indicating TFs (triangles), target genes (circles), and putative enhancers/ATAC peaks (edges). Nodes are colored according to disease status in astrocytes, with coloring scheme as in figure 5B (left graph). Stacked bar plot of astrocyte hDECORE network with quantification of disease status. **(C)** Human developmental timeline of hDECORE TFs and target gene expression in ~370,000 nuclei and 106 individuals. Each dot represents a nucleus, with x-axis indicating age (days, log scale) and y-axis indicates module score for this given gene set. Gene set averages are quantified and fitted with line of best fit for TFs (red line), target genes (blue line), and a randomized background gene set (grey line). (**D**) Independent CHi-C validation of predicted HAR->target gene interactions of HARs in the hiDEcore network. Interactions were categorized on whether they matched that of our analysis (blue), mismatched (light red), or no gene targets were identified in CHi-C assay (grey). Numbers indicate counts of unique HARs, with percentages indicated in parentheses.

Table S1. Primate prefrontal cortex snRNAseq samples used in this study. Indicated are both samples generated in this study (GEO submission GSE294786), along with adult primate prefrontal cortex samples obtained from a published dataset by Ma (*26*). Ages are in years unless indicated otherwise; *PMD (postmortem delay), indicated in hours. *HSA (*Homo sapiens*; human), PTR (*Pan troglodytes*; chimpanzee), RMA (*Macaca mulatta*; rhesus). *HBCC (Human Brain Collection Core), BPRC (Biomedical Primate Research Center), YSM (Yale School of Medicine), UMB BTB (University of Maryland, Baltimore, Brain & Tissue Bank), NCBR (National Chimpanzee Brain Resource).

species	age	source	PMD	ID	cause of death	sex	study
HSA	2 months	HBCC	36	1452	asphyxia	M	This
HSA	4 months	HBCC	29	1490	asphyxia	M	This
HSA	4 months	HBCC	20	1623	asphyxia	M	This
HSA	24	HBCC	36	1350	bronchial asthma	M	This
HSA	36	HBCC	22	1385	blunt force injury (chest)	M	This
PTR	0 months	BPRC	5	Chimp2	euthanasia due to parental neglect	M	This
PTR	5 months	BPRC	5	Chimp3	euthanasia due to parental neglect	M	This
PTR	50	BPRC	7	Frits	cardiomyopathy	M	This
PTR	18	BPRC	7	Ruben	cardiomyopathy, pneumonia	M	This
PTR	35	BPRC	7	Bart	spontaneous death	M	This
RMA	0 months	BPRC	<1	R12121	euthanasia due to parental neglect	M	This
RMA	0 months	BPRC	<1	R17043	euthanasia due to parental neglect	M	This
HSA	64	YSM	4	HSB106	-	M	Ma (*26*)
HSA	50	YSM	19	HSB628	-	F	Ma (*26*)
HSA	36	UMB BTB	24	HSB189	-	M	Ma (*26*)
HSA	19	UMB BTB	11	HSB340	-	M	Ma (*26*)
PTR	31	NCBR	<5	PTB165	-	M	Ma (*26*)
PTR	27	NCBR	<5	PTB166	-	M	Ma (*26*)
PTR	50	NCBR	<1	PTB1841	-	F	Ma (*26*)
PTR	45	NCBR	<1	PTB2169	-	F	Ma (*26*)
RMA	11	YSM	<1	RMB161	-	F	Ma (*26*)
RMA	11	YSM	<1	RMB196	-	F	Ma (*26*)
RMA	8	YSM	<1	RMB295	-	F	Ma (*26*)
RMA	11	YSM	<1	RMB307	-	F	Ma (*26*)

Table S2. Human snRNAseq frontal cortex atlas samples used in this study, originating from Velmeshev (*24*), Ramos (*82*), Herring (*27*). Age column represents years, unless indicated otherwise. *GW (gestational week); *tm (trimester); *FC (frontal cortex). *PMD (postmortem delay), indicated in hours.

sample	dataset	ID	age	age_range	sex	region	PMD
5387_BA9	Velmeshev	5387	12	10-20 y	M	FC	13
5577_BA9	Velmeshev	5577	21	Adult	M	FC	19
5958_BA9	Velmeshev	5958	22	Adult	M	FC	24
5893_PFC	Velmeshev	5893	19	10-20 y	M	FC	11
4341_BA46	Velmeshev	4341	13	10-20 y	M	FC	16
5538_PFC	Velmeshev	5538	19	10-20 y	F	FC	24
5408_PFC	Velmeshev	5408	6	4-10 y	M	FC	16
5879_PFC	Velmeshev	5879	15	10-20 y	M	FC	18
5936_PFC	Velmeshev	5936	14	10-20 y	F	FC	23
5976_BA9	Velmeshev	5976	4	4-10 y	F	FC	21
5787_BA9	Velmeshev	5787	39	Adult	M	FC	16
5609_BA9	Velmeshev	5609	54	Adult	F	FC	6
5546_BA9	Velmeshev	5546	34	Adult	F	FC	27
5981_BA9	Velmeshev	5981	44	Adult	F	FC	19
4426_BA9	Velmeshev	4426	2.2	2-4 y	M	FC	34
4350_BA9	Velmeshev	4350	0.54	0-1 y	M	FC	20
4369_BA9	Velmeshev	4369	2.7	2-4 y	F	FC	14
A14-103_FC	Velmeshev	A14-103	35 GW	3rd tm	F	FC	18
A15-44_FC	Velmeshev	A15-44	39 GW	3rd tm	F	FC	20
4389_BA9	Velmeshev	4389	0.2	0-1 y	F	FC	27
5608_BA9	Velmeshev	5608	3.28	2-4 y	M	FC	29
4396_BA9	Velmeshev	4396	0.15	0-1 y	M	FC	15
1798_BA9	Velmeshev	1798	1.5	1-2 y	F	FC	24
4942_BA9	Velmeshev	4942	GW25	2nd tm	F	FC	16
779_BA9	Velmeshev	779	GW37	3rd tm	M	FC	5
4283_BA9	Velmeshev	4283	GW27	2nd tm	F	FC	19
5900_BA9	Velmeshev	5900	GW34	3rd tm	F	FC	23
GW27-PFC-2-7-18	Velmeshev	GW27-2-7-18	GW28.5	3rd tm	M	FC	24
GW20-PFC-11-28-18	Velmeshev	GW20-11-28-18	GW20.5	2nd tm	F	FC	20
GW20-PFC-12-7-18	Velmeshev	GW20-12-7-18	GW20	2nd tm	F	FC	0
GW25-PFC-11-17-17	Velmeshev	GW25-11-17-17	GW25	2nd tm	M	FC	0
GW30-PFC-11-6-18	Velmeshev	GW30-11-6-18	GW30	3rd tm	F	FC	24
GW16-2-2-20_PFC	Velmeshev	GW16-2-2-20	GW16	2nd tm	M	FC	0
GW18-6-14-19_PFC	Velmeshev	GW18-6-14-19	GW18	2nd tm	M	FC	0
9C	Ramos	9	GW17	2nd tm	F	FC	40
11C	Ramos	11	GW20	2nd tm	F	FC	24
24C	Ramos	24	GW18	2nd tm	M	FC	4
26C	Ramos	26	GW19	2nd tm	M	FC	7

23C	Ramos	23	GW24	2nd tm	M	FC	12
30C	Ramos	30	GW22	2nd tm	M	FC	28
34C	Ramos	34	GW20.5	2nd tm	M	FC	24
56C	Ramos	56	GW21	2nd tm	F	FC	18
63C	Ramos	63	GW24	2nd tm	M	FC	24
3C	Ramos	3	GW25	2nd tm	F	FC	24
62C	Ramos	62	GW24	2nd tm	M	FC	23
64C	Ramos	64	GW24	2nd tm	M	FC	36
6C	Ramos	6	GW41	3rd tm	F	FC	48
10C	Ramos	10	GW33	3rd tm	M	FC	24
60C	Ramos	60	GW32	3rd tm	F	FC	96
9G	Ramos	9	GW17	2nd tm	F	FC	40
11G	Ramos	11	GW20	2nd tm	F	FC	24
24G	Ramos	24	GW18	2nd tm	M	FC	4
26G	Ramos	26	GW19	2nd tm	M	FC	7
23G	Ramos	23	GW24	2nd tm	M	FC	12
30G	Ramos	30	GW22	2nd tm	M	FC	28
34G	Ramos	34	GW20.5	2nd tm	M	FC	24
56G	Ramos	56	GW21	2nd tm	F	FC	18
63G	Ramos	63	GW24	2nd tm	M	FC	24
3G	Ramos	3	GW25	2nd tm	F	FC	24
62G	Ramos	62	GW24	2nd tm	M	FC	23
64G	Ramos	64	GW24	2nd tm	M	FC	36
6G	Ramos	6	GW41	3rd tm	F	FC	48
10G	Ramos	10	GW33	3rd tm	M	FC	24
60G	Ramos	60	GW32	3rd tm	F	FC	96
RL1612	Herring	RL1612	0.09	0-1 y	M	FC	5
RL1613	Herring	RL1613	2.08	2-4 y	M	FC	12
RL1614	Herring	RL1614	8	4-10 y	M	FC	16
RL1777	Herring	RL1777	0.005	0-1 y	F	FC	26
RL1786	Herring	RL1786	2.1	2-4 y	M	FC	12
RL2100	Herring	RL2100	0.2	0-1 y	M	FC	13
RL2102	Herring	RL2102	16	10-20 y	F	FC	23
RL2103	Herring	RL2103	ga22	2nd tm	M	FC	5
RL2104	Herring	RL2104	0.3	0-1 y	F	FC	21
RL2105	Herring	RL2105	1.5	2-4 y	M	FC	25
RL2106	Herring	RL2106	6.5	4-10 y	M	FC	16
RL2107	Herring	RL2107	ga24	2nd tm	M	FC	9
RL2108	Herring	RL2108	0.5	0-1 y	M	FC	11
RL2109	Herring	RL2109	4	4-10 y	M	FC	25
RL2110	Herring	RL2110	10	10-20 y	M	FC	15
RL2121	Herring	RL2121	ga34	3rd tm	F	FC	23
RL2122	Herring	RL2122	0.8	0-1 y	M	FC	18
RL2123	Herring	RL2123	20	Adult	F	FC	19

RL2124	Herring	RL2124	40	Adult	M	FC	9
RL2125	Herring	RL2125	1.1	2-4 y	F	FC	20
RL2126	Herring	RL2126	10	10-20 y	F	FC	22
RL2127	Herring	RL2127	12	10-20 y	M	FC	13
RL2128	Herring	RL2128	20	Adult	F	FC	17
RL2129	Herring	RL2129	3	2-4 y	F	FC	12
RL2130	Herring	RL2130	14	10-20 y	F	FC	23
RL2131	Herring	RL2131	17	10-20 y	F	FC	12
RL2132	Herring	RL2132	25	Adult	F	FC	24

Data S1. (separate file)

Zip file containing Supplementary tables S1-S9

REFERENCES

1. A. M. M. M. Sousa, K. A. Meyer, G. Santpere, F. O. Gulden, N. Sestan, Evolution of the human nervous system function, structure, and development. *Cell* **170**, 226–247 (2017).

2. T. Sakai, S. Hirata, K. Fuwa, K. Sugama, K. Kusunoki, H. Makishima, T. Eguchi, S. Yamada, N. Ogihara, H. Takeshita, Fetal brain development in chimpanzees versus humans. *Curr. Biol.* **22**, R791–R792 (2012).

3. D. J. Miller, T. Duka, C. D. Stimpson, S. J. Schapiro, W. B. Baze, M. J. McArthur, A. J. Fobbs, A. M. M. Sousa, N. Sestan, D. E. Wildman, L. Lipovich, C. W. Kuzawa, P. R. Hof, C. C. Sherwood, Prolonged myelination in human neocortical evolution. *Proc. Natl. Acad. Sci. U.S.A.* **109**, 16480–16485 (2012).

4. B. Castelijns, M. L. Baak, I. S. Timpanaro, C. R. M. Wiggers, M. W. Vermunt, P. Shang, I. Kondova, G. Geeven, V. Bianchi, W. de Laat, N. Geijsen, M. P. Creyghton, Hominin-specific regulatory elements selectively emerged in oligodendrocytes and are disrupted in autism patients. *Nat. Commun.* **11**, 301 (2020).

5. E. Khrameeva, I. Kurochkin, D. Han, P. Guijarro, S. Kanton, M. Santel, Z. Qian, S. Rong, P. Mazin, M. Sabirov, M. Bulat, O. Efimova, A. Tkachev, S. Guo, C. C. Sherwood, J. Gray Camp, S. Pääbo, B. Treutlein, P. Khaitovich, Single-cell-resolution transcriptome map of human, chimpanzee, bonobo, and macaque brains. *Genome Res.* **30**, 776–789 (2020).

6. M. C. Oldham, G. Konopka, K. Iwamoto, P. Langfelder, T. Kato, S. Horvath, D. H. Geschwind, Functional organization of the transcriptome in human brain. *Nat. Neurosci.* **11**, 1271–1282 (2008).

7. G. Konopka, T. Friedrich, J. Davis-Turak, K. Winden, M. C. Oldham, F. Gao, L. Chen, G. Z. Wang, R. Luo, T. M. Preuss, D. H. Geschwind, Human-specific transcriptional networks in the brain. *Neuron* **75**, 601–617 (2012).

8. M. Somel, X. Liu, P. Khaitovich, Human brain evolution: Transcripts, metabolites and their regulators. *Nat. Rev. Neurosci.* **14**, 112–127 (2013).

9. Z. He, D. Han, O. Efimova, P. Guijarro, Q. Yu, A. Oleksiak, S. Jiang, K. Anokhin, B. Velichkovsky, S. Grünewald, P. Khaitovich, Comprehensive transcriptome analysis of neocortical layers in humans, chimpanzees and macaques. *Nat. Neurosci.* **20**, 886–895 (2017).

10. D. Brawand, M. Soumillon, A. Necsulea, P. Julien, G. Csardi, P. Harrigan, M. Weier, A. Liechti, A. Aximu-Petri, M. Kircher, F. W. Albert, U. Zeller, P. Khaitovich, F. Grutzner, S. Bergmann, R. Nielsen, S. Paabo, H. Kaessmann, The evolution of gene expression levels in mammalian organs. *Nature* **478**, 343–348 (2011).

11. M. W. Vermunt, S. C. Tan, B. Castelijns, G. Geeven, P. Reinink, E. De Bruijn, I. Kondova, S. Persengiev, R. Bontrop, E. Cuppen, W. De Laat, M. P. Creyghton, Epigenomic annotation of gene regulatory alterations during evolution of the primate brain. *Nat. Neurosci.* **19**, 494–503 (2016).

12. S. Kanton, M. J. Boyle, Z. He, M. Santel, A. Weigert, F. Sanchís-Calleja, P. Guijarro, L. Sidow, J. S. Fleck, D. Han, Z. Qian, M. Heide, W. B. Huttner, P. Khaitovich, S. Pääbo, B. Treutlein, J. G. Camp, Organoid single-cell genomic atlas uncovers human-specific features of brain development. *Nature* **574**, 418–422 (2019).

13. T. Otani, M. C. Marchetto, F. H. Gage, B. D. Simons, F. J. Livesey, 2D and 3D stem cell models of primate cortical development identify species-specific differences in progenitor behavior contributing to brain size. *Cell Stem Cell* **18**, 467–480 (2016).

14. F. Mora-Bermúdez, F. Badsha, S. Kanton, J. G. Camp, B. Vernot, K. Köhler, B. Voigt, K. Okita, T. Maricic, Z. He, R. Lachmann, S. Pääbo, B. Treutlein, W. B. Huttner, A. Musacchio, Differences and similarities between human and chimpanzee neural progenitors during cerebral cortex development. *Elife* **5**, e18683 (2016).

15. S. K. Reilly, J. Yin, A. E. Ayoub, D. Emera, J. Leng, J. Cotney, R. Sarro, P. Rakic, J. P. Noonan, Evolutionary genomics. Evolutionary changes in promoter and enhancer activity during human corticogenesis. *Science* **347**, 1155–1159 (2015).

16. Y. Zhu, A. M. M. Sousa, T. Gao, M. Skarica, M. Li, G. Santpere, P. Esteller-Cucala, D. Juan, L. Ferrández-Peral, F. O. Gulden, M. Yang, D. J. Miller, T. Marques-Bonet, Y. Imamura

Kawasawa, H. Zhao, N. Sestan, Spatiotemporal transcriptomic divergence across human and macaque brain development. *Science* **362**, eaat8077 (2018).

17. E. Di Lullo, A. R. Kriegstein, The use of brain organoids to investigate neural development and disease. *Nat. Rev. Neurosci.* **18**, 573–584 (2017).

18. A. Bhaduri, M. G. Andrews, W. Mancia Leon, D. D. Jung, D. Shin, D. Allen, D. D. Jung, G. Schmunk, M. Haeussler, J. Salma, A. A. Pollen, T. J. Nowakowski, A. R. Kriegstein, Cell stress in cortical organoids impairs molecular subtype specification. *Nature* **578**, 142–148 (2020).

19. A. S. Nord, M. J. Blow, C. Attanasio, J. A. Akiyama, A. Holt, R. Hosseini, S. Phouanenavong, I. Plajzer-Frick, M. Shoukry, V. Afzal, J. L. R. Rubenstein, E. M. Rubin, L. A. Pennacchio, A. Visel, Rapid and pervasive changes in genome-wide enhancer usage during mammalian development. *Cell* **155**, 1521–1531 (2013).

20. M. Makinodan, K. M. Rosen, S. Ito, G. Corfas, A critical period for social experience-dependent oligodendrocyte maturation and myelination. *Science* **337**, 1357–1360 (2012).

21. K. Roy, J. C. Murtie, B. F. El-Khodor, N. Edgar, S. P. Sardi, B. M. Hooks, M. Benoit-Marand, C. Chen, H. Moore, P. O'Donnell, D. Brunner, G. Corfas, Loss of erbB signaling in oligodendrocytes alters myelin and dopaminergic function, a potential mechanism for neuropsychiatric disorders. *Proc. Natl. Acad. Sci. U.S.A.* **104**, 8131–8136 (2007).

22. H. J. Kang, Y. I. Kawasawa, F. Cheng, Y. Zhu, X. Xu, M. Li, A. M. Sousa, M. Pletikos, K. A. Meyer, G. Sedmak, T. Guennel, Y. Shin, M. B. Johnson, Z. Krsnik, S. Mayer, S. Fertuzinhos, S. Umlauf, S. N. Lisgo, A. Vortmeyer, D. R. Weinberger, S. Mane, T. M. Hyde, A. Huttner, M. Reimers, J. E. Kleinman, N. Sestan, Spatio-temporal transcriptome of the human brain. *Nature* **478**, 483–489 (2011).

23. E. Caglayan, Y. Liu, G. Konopka, Neuronal ambient RNA contamination causes misinterpreted and masked cell types in brain single-nuclei datasets. *Neuron* **110**, 4043–4056.e5 (2022).

24. D. Velmeshev, Y. Perez, Z. Yan, J. E. Valencia, D. R. Castaneda-Castellanos, L. Wang, L. Schirmer, S. Mayer, B. Wick, S. Wang, T. J. Nowakowski, M. Paredes, E. J. Huang, A. R. Kriegstein, Single-cell analysis of prenatal and postnatal human cortical development. *Science* **382**, eadf0834 (2023).

25. K. Polański, M. D. Young, Z. Miao, K. B. Meyer, S. A. Teichmann, J. E. Park, BBKNN: Fast batch alignment of single cell transcriptomes. *Bioinformatics* **36**, 964–965 (2020).

26. S. Ma, M. Skarica, Q. Li, C. Xu, R. D. Risgaard, A. T. N. Tebbenkamp, X. Mato-Blanco, R. Kovner, Ž. Krsnik, X. de Martin, V. Luria, X. Martí-Pérez, D. Liang, A. Karger, D. K. Schmidt, Z. Gomez-Sanchez, C. Qi, K. T. Gobeske, S. Pochareddy, A. Debnath, C. J. Hottman, J. Spurrier, L. Teo, A. G. Boghdadi, J. Homman-Ludiye, J. J. Ely, E. W. Daadi, D. Mi, M. Daadi, O. Marín, P. R. Hof, M. R. Rasin, J. Bourne, C. C. Sherwood, G. Santpere, M. J. Girgenti, S. M. Strittmatter, A. M. M. Sousa, N. Sestan, Molecular and cellular evolution of the primate dorsolateral prefrontal cortex. *Science* **377**, eabo7257 (2022).

27. C. A. Herring, R. K. Simmons, S. Freytag, D. Poppe, J. J. D. Moffet, J. Pflueger, S. Buckberry, D. B. Vargas-Landin, O. Clément, E. G. Echeverría, G. J. Sutton, A. Alvarez-Franco, R. Hou, C. Pflueger, K. McDonald, J. M. Polo, A. R. R. Forrest, A. K. Nowak, I. Voineagu, L. Martelotto, R. Lister, Human prefrontal cortex gene regulatory dynamics from gestation to adulthood at single-cell resolution. *Cell* **185**, 4428–4447.e28 (2022).

28. A. Fujita, B. Isidor, H. Piloquet, P. Corre, N. Okamoto, M. Nakashima, Y. Tsurusaki, H. Saitsu, N. Miyake, N. Matsumoto, De novo MEIS2 mutation causes syndromic developmental delay with persistent gastro-esophageal reflux. *J. Hum. Genet.* **61**, 835–838 (2016).

29. M. K. Fard, F. Van der Meer, P. Sánchez, L. Cantuti-Castelvetri, S. Mandad, S. Jäkel, E. F. Fornasiero, S. Schmitt, M. Ehrlich, L. Starost, T. Kuhlmann, C. Sergiou, V. Schultz, C. Wrzos, W. Brück, H. Urlaub, L. Dimou, C. Stadelmann, M. Simons, BCAS1 expression defines a population of early myelinating oligodendrocytes in multiple sclerosis lesions. *Sci. Transl. Med.* **9**, 7816 (2017).

30. T. Ishimoto, K. Ninomiya, R. Inoue, M. Koike, Y. Uchiyama, H. Mori, Mice lacking BCAS1, a novel myelin-associated protein, display hypomyelination, schizophrenia-like abnormal behaviors, and upregulation of inflammatory genes in the brain. *Glia* **65**, 727–739 (2017).

31. E. Caglayan, F. Ayhan, Y. Liu, R. M. Vollmer, E. Oh, C. C. Sherwood, T. M. Preuss, S. V. Yi, G. Konopka, Molecular features driving cellular complexity of human brain evolution. *Nature* **620**, 145–153 (2023).

32. A. Benítez-Burraco, M. S. Jiménez-Romero, M. Fernández-Urquiza, Delving into the genetic causes of language impairment in a case of partial deletion of NRXN1. *Mol. Syndromol.* **13**, 496 (2023).

33. G. Liu, J. Peng, Z. Liao, J. J. Locascio, J. C. Corvol, F. Zhu, X. Dong, J. Maple-Grødem, M. C. Campbell, A. Elbaz, S. Lesage, A. Brice, G. Mangone, J. H. Growdon, A. Y. Hung, M. A. Schwarzschild, M. T. Hayes, A. M. Wills, T. M. Herrington, B. Ravina, I. Shoulson, P. Taba, S. Kõks, T. G. Beach, F. Cormier-Dequaire, G. Alves, O. B. Tysnes, J. S. Perlmutter, P. Heutink, S. S. Amr, J. J. van Hilten, M. Kasten, B. Mollenhauer, C. Trenkwalder, C. Klein, R. A. Barker, C. H. Williams-Gray, J. Marinus, J. J. van Hilten, C. R. Scherzer, Genome-wide survival study identifies a novel synaptic locus and polygenic score for cognitive progression in Parkinson's disease. *Nat. Genet.* **53**, 787–793 (2021).

34. T. J. Nowakowski, A. Bhaduri, A. A. Pollen, B. Alvarado, M. A. Mostajo-Radji, E. Di Lullo, M. Haeussler, C. Sandoval-Espinosa, S. J. Liu, D. Velmeshev, J. R. Ounadjela, J. Shuga, X. Wang, D. A. Lim, J. A. West, A. A. Leyrat, W. J. Kent, A. R. Kriegstein, Spatiotemporal gene expression trajectories reveal developmental hierarchies of the human cortex. *Science* **358**, 1318–1323 (2017).

35. D. Dimitrov, D. Türei, M. Garrido-Rodriguez, P. L. Burmedi, J. S. Nagai, C. Boys, R. O. Ramirez Flores, H. Kim, B. Szalai, I. G. Costa, A. Valdeolivas, A. Dugourd, J. Saez-Rodriguez, Comparison of methods and resources for cell-cell communication inference from single-cell RNA-Seq data. *Nat. Commun.* **13**, 3224 (2022).

36. J. Heinke, L. Wehofsits, Q. Zhou, C. Zoeller, K. M. Baar, T. Helbing, A. Laib, H. Augustin, C. Bode, C. Patterson, M. Moser, BMPER is an endothelial cell regulator and controls bone morphogenetic protein-4-dependent angiogenesis. *Circ. Res.* **103**, 804–812 (2008).

37. D. Villar, C. Berthelot, S. Aldridge, T. F. Rayner, M. Lukk, M. Pignatelli, T. J. Park, R. Deaville, J. T. Erichsen, A. J. Jasinska, J. M. Turner, M. F. Bertelsen, E. P. Murchison, P. Flicek, D. T. Odom, Enhancer evolution across 20 mammalian species. *Cell* **160**, 554–566 (2015).

38. C. Bravo González-Blas, S. De Winter, G. Hulselmans, N. Hecker, I. Matetovici, V. Christiaens, S. Poovathingal, J. Wouters, S. Aibar, S. Aerts, SCENIC+: Single-cell multiomic inference of enhancers and gene regulatory networks. *Nat. Methods* **20**, 1355–1367 (2023).

39. L. A. Pennacchio, N. Ahituv, A. M. Moses, S. Prabhakar, M. A. Nobrega, M. Shoukry, S. Minovitsky, I. Dubchak, A. Holt, K. D. Lewis, I. Plajzer-Frick, J. Akiyama, S. De Val, V. Afzal, B. L. Black, O. Couronne, M. B. Eisen, A. Visel, E. M. Rubin, In vivo enhancer analysis of human conserved non-coding sequences. *Nature* **444**, 499–502 (2006).

40. N. L. Jorstad, J. H. T. Song, D. Exposito-Alonso, H. Suresh, N. Castro-Pacheco, F. M. Krienen, A. M. Yanny, J. Close, E. Gelfand, B. Long, S. C. Seeman, K. J. Travaglini, S. Basu, M. Beaudin, D. Bertagnolli, M. Crow, S. L. Ding, J. Eggermont, A. Glandon, J. Goldy, K. Kiick, T. Kroes, D. McMillen, T. Pham, C. Rimorin, K. Siletti, S. Somasundaram, M. Tieu, A. Torkelson, G. Feng, W. D. Hopkins, T. Höllt, C. D. Keene, S. Linnarsson, S. A. McCarroll, B. P. Lelieveldt, C. C. Sherwood, K. Smith, C. A. Walsh, A. Dobin, J. Gillis, E. S. Lein, R. D. Hodge, T. E. Bakken, Comparative transcriptomics reveals human-specific cortical features. *Science* **382**, eade9516 (2023).

41. P. J. Wittkopp, G. Kalay, Cis-regulatory elements: Molecular mechanisms and evolutionary processes underlying divergence. *Nat. Rev. Genet.* **13**, 59–69 (2012).

42. P. S. Emani, J. J. Liu, D. Clarke, M. Jensen, J. Warrell, C. Gupta, R. Meng, C. Y. Lee, S. Xu, C. Dursun, S. Lou, Y. Chen, Z. Chu, T. Galeev, A. Hwang, Y. Li, P. Ni, X. Zhou, T. E. Bakken, J. Bendl, L. Bicks, T. Chatterjee, L. Cheng, Y. Cheng, Y. Dai, Z. Duan, M. Flaherty, J. F. Fullard, M. Gancz, D. Garrido-Martín, S. Gaynor-Gillett, J. Grundman, N. Hawken, E.

Henry, G. E. Hoffman, A. Huang, Y. Jiang, T. Jin, N. L. Jorstad, R. Kawaguchi, S. Khullar, J. Liu, J. Liu, S. Liu, S. Ma, M. Margolis, S. Mazariegos, J. Moore, J. R. Moran, E. Nguyen, N. Phalke, M. Pjanic, H. Pratt, D. Quintero, A. S. Rajagopalan, T. R. Riesenmy, N. Shedd, M. Shi, M. Spector, R. Terwilliger, K. J. Travaglini, B. Wamsley, G. Wang, Y. Xia, S. Xiao, A. C. Yang, S. Zheng, M. J. Gandal, D. Lee, E. S. Lein, P. Roussos, N. Sestan, Z. Weng, K. P. White, H. Won, M. J. Girgenti, J. Zhang, D. Wang, D. Geschwind, M. Gerstein, S. Akbarian, A. Abyzov, N. Ahituv, D. Arasappan, J. J. Almagro Armenteros, B. J. Beliveau, S. Berretta, R. A. Bharadwaj, A. Bhattacharya, K. Brennand, D. Capauto, F. A. Champagne, C. Chatzinakos, H. I. Chen, L. Cheng, A. Chess, J. Chien, A. Clement, L. Collado-Torres, G. M. Cooper, G. E. Crawford, R. Dai, N. P. Daskalakis, J. Davila-Velderrain, A. Deep-Soboslay, C. Deng, C. P. DiPietro, S. Dracheva, S. Drusinsky, D. Duong, N. J. Eagles, J. Edelstein, K. Galani, K. Girdhar, F. S. Goes, W. Greenleaf, H. Guo, Q. Guo, Y. Hadas, J. Hallmayer, X. Han, V. Haroutunian, C. He, S. C. Hicks, M. Ho, L.-L. Ho, Y. Huang, L. A. Huuki-Myers, T. M. Hyde, A. Iatrou, F. Inoue, A. Jajoo, L. Jiang, P. Jin, C. Jops, A. Jourdon, M. Kellis, J. E. Kleinman, S. P. Kleopoulos, A. Kozlenkov, A. Kriegstein, A. Kundaje, S. Kundu, J. Li, M. Li, X. Lin, S. Liu, C. Liu, J. M. Loupe, D. Lu, L. Ma, J. Mariani, K. Martinowich, K. R. Maynard, R. M. Myers, C. Micallef, T. Mikhailova, G. Ming, S. Mohammadi, E. Monte, K. S. Montgomery, E. A. Mukamel, A. C. Nairn, C. B. Nemeroff, S. Norton, T. Nowakowski, L. Omberg, S. C. Page, S. Park, A. Patowary, R. Pattni, G. Pertea, M. A. Peters, D. Pinto, S. Pochareddy, K. S. Pollard, A. Pollen, P. F. Przytycki, C. Purmann, Z. S. Qin, P.-P. Qu, T. Raj, S. Reach, T. Reimonn, K. J. Ressler, D. Ross, J. Rozowsky, M. Ruth, W. B. Ruzicka, S. J. Sanders, J. M. Schneider, S. Scuderi, R. Sebra, N. Seyfried, Z. Shao, A. W. Shieh, J. H. Shin, M. Skarica, C. Snijders, H. Song, M. W. State, J. Stein, M. Steyert, S. Subburaju, T. Sudhof, M. Snyder, R. Tao, K. Therrien, L.-H. Tsai, A. E. Urban, F. M. Vaccarino, H. van Bakel, D. Vo, G. Voloudakis, T. Wang, S. H. Wang, Y. Wang, Y. Wei, A. K. Weimer, D. R. Weinberger, C. Wen, S. Whalen, A. J. Willsey, W. Wong, H. Wu, F. Wu, S. Wuchty, D. Wylie, C. X. Yap, B. Zeng, P. Zhang, C. Zhang, B. Zhang, Y. Zhang, R. Ziffra, Z. R. Zeier, T. M. Zintel, Single-cell genomics and regulatory networks for 388 human brains. *Science* **384**, eadi5199 (2024).

43. M. Fossati, R. Pizzarelli, E. R. Schmidt, J. V. Kupferman, D. Stroebel, F. Polleux, C. Charrier, SRGAP2 and its human-specific paralog co-regulate the development of excitatory and inhibitory synapses. *Neuron* **91**, 356–369 (2016).

44. T. C. Südhof, Synaptic neurexin complexes: A molecular code for the logic of neural circuits. *Cell* **171**, 745–769 (2017).

45. H. Won, J. Huang, C. K. Opland, C. L. Hartl, D. H. Geschwind, Human evolved regulatory elements modulate genes involved in cortical expansion and neurodevelopmental disease susceptibility. *Nat. Commun.* **10**, 2396 (2019).

46. J. M. McClellan, A. W. Zoghbi, J. D. Buxbaum, C. Cappi, J. J. Crowley, J. Flint, D. E. Grice, S. Gulsuner, C. Iyegbe, S. Jain, P. H. Kuo, M. C. Lattig, M. R. Passos-Bueno, M. Purushottam, D. J. Stein, A. B. Sunshine, E. S. Susser, C. A. Walsh, O. Wootton, M. C. King, An evolutionary perspective on complex neuropsychiatric disease. *Neuron* **112**, 7–24 (2024).

47. L. Liu, Y. Zhang, G. Niu, Q. Li, Z. Li, T. Zhu, C. Feng, X. Liu, Y. Zhang, T. Xu, R. Chen, X. Teng, R. Zhang, D. Zou, L. Ma, Z. Zhang, BrainBase: A curated knowledgebase for brain disease. *Nucleic Acids Res.* **50**, D1131–D1138 (2022).

48. B. Wamsley, L. Bicks, Y. Cheng, R. Kawaguchi, D. Quintero, M. Margolis, J. Grundman, J. Liu, S. Xiao, N. Hawken, S. Mazariegos, D. H. Geschwind, Molecular cascades and cell type–specific signatures in ASD revealed by single-cell genomics. *Science* **384**, eadh2606 (2024).

49. M. R. Cookson, Evolution of neurodegeneration. *Curr. Biol.* **22**, R753–R761 (2012).

50. A. M. Jeffries, T. Yu, J. S. Ziegenfuss, A. K. Tolles, C. E. Baer, C. B. Sotelo, Y. Kim, Z. Weng, M. A. Lodato, Single-cell transcriptomic and genomic changes in the ageing human brain. *Nature* **646**, 657–666 (2025).

51. Y. Yang, S. Chen, L. Zhang, G. Zhang, Y. Liu, Y. Li, L. Zou, L. Meng, Y. Tian, L. Dai, M. Xiong, L. Pan, J. Xiong, L. Chen, H. Hou, Z. Yu, Z. Zhang, The PM20D1-NADA pathway protects against Parkinson's disease. *Cell Death Differ.* **31**, 1545–1560 (2024).

52. J. Z. Long, K. J. Svensson, L. A. Bateman, H. Lin, T. Kamenecka, I. A. Lokurkar, J. Lou, R. R. Rao, M. R. R. Chang, M. P. Jedrychowski, J. A. Paulo, S. P. Gygi, P. R. Griffin, D. K.

Nomura, B. M. Spiegelman, The secreted enzyme PM20D1 regulates lipidated amino acid uncouplers of mitochondria. *Cell* **166**, 424–435 (2016).

53. N. Song, Y. Fang, H. Zhu, J. Liu, S. Jiang, S. Sun, R. Xu, J. Ding, G. Hu, M. Lu, Kir6.2 is essential to maintain neurite features by modulating PM20D1-reduced mitochondrial ATP generation. *Redox Biol.* **47**, 102168 (2021).

54. Q. Wang, M. Wang, I. Choi, L. Sarrafha, M. Liang, L. Ho, K. Farrell, K. G. Beaumont, R. Sebra, C. De Sanctis, J. F. Crary, T. Ahfeldt, J. Blanchard, D. Neavin, J. Powell, D. A. Davis, X. Sun, B. Zhang, Z. Yue, Molecular profiling of human substantia nigra identifies diverse neuron types associated with vulnerability in Parkinson's disease. *Sci. Adv.* **10**, eadi8287 (2024).

55. D. J. Miller, P. E. Fort, Heat shock proteins regulatory role in neurodevelopment. *Front. Neurosci.* **12**, 412741 (2018).

56. W. Luo, W. Sun, T. Taldone, A. Rodina, G. Chiosis, Heat shock protein 90 in neurodegenerative diseases. *Mol. Neurodegener.* **5**, 24 (2010).

57. H. M. Mansour, A. F. Mohamed, M. M. Khattab, A. S. El-Khatib, Heat shock protein 90 in Parkinson's disease: Profile of a serial killer. *Neuroscience* **537**, 32–46 (2024).

58. B. Porton, W. C. Wetsel, H. T. Kao, Synapsin III: Role in neuronal plasticity and disease. *Semin. Cell Dev. Biol.* **22**, 416–424 (2011).

59. G. Faustini, F. Longhena, A. Masato, V. Bassareo, R. Frau, T. Klingstedt, H. Shirani, V. Brembati, E. Parrella, M. Vezzoli, K. P. R. Nilsson, M. Pizzi, M. G. Spillantini, L. Bubacco, A. Bellucci, Synapsin III gene silencing redeems alpha-synuclein transgenic mice from Parkinson's disease-like phenotype. *Mol. Ther.* **30**, 1465–1483 (2022).

60. G. Y. Ou, W. W. Lin, W. J. Zhao, Neuregulins in neurodegenerative diseases. *Front. Aging Neurosci.* **13**, 662474 (2021).

61. Y. Takahashi, Y. Fukuda, J. Yoshimura, A. Toyoda, K. Kurppa, H. Moritoyo, V. V. Belzil, P. A. Dion, K. Higasa, K. Doi, H. Ishiura, J. Mitsui, H. Date, B. Ahsan, T. Matsukawa, Y.

Ichikawa, T. Moritoyo, M. Ikoma, T. Hashimoto, F. Kimura, S. Murayama, O. Onodera, M. Nishizawa, M. Yoshida, N. Atsuta, G. Sobue, J. Cals, J. A. Fifita, K. L. Williams, I. P. Blair, G. A. Nicholson, P. Gonzalez-Perez, R. H. Brown, M. Nomoto, K. Elenius, G. A. Rouleau, A. Fujiyama, S. Morishita, J. Goto, S. Tsuji, ERBB4 mutations that disrupt the neuregulin-ErbB4 pathway cause amyotrophic lateral sclerosis type 19. *Am. J. Hum. Genet.* **93**, 900–905 (2013).

62. Z. N. Kronenberg, I. T. Fiddes, D. Gordon, S. Murali, S. Cantsilieris, O. S. Meyerson, J. G. Underwood, B. J. Nelson, M. J. P. Chaisson, M. L. Dougherty, K. M. Munson, A. R. Hastie, M. Diekhans, F. Hormozdiari, N. Lorusso, K. Hoekzema, R. Qiu, K. Clark, A. Raja, A. E. Welch, M. Sorensen, C. Baker, R. S. Fulton, J. Armstrong, T. A. Graves-Lindsay, A. M. Denli, E. R. Hoppe, P. Hsieh, C. M. Hill, A. W. C. Pang, J. Lee, E. T. Lam, S. K. Dutcher, F. H. Gage, W. C. Warren, J. Shendure, D. Haussler, V. A. Schneider, H. Cao, M. Ventura, R. K. Wilson, B. Paten, A. Pollen, E. E. Eichler, High-resolution comparative analysis of great ape genomes. *Science* **360**, eaar6343 (2018).

63. K. M. Girskis, A. B. Stergachis, E. M. DeGennaro, R. N. Doan, X. Qian, M. B. Johnson, P. P. Wang, G. M. Sejourne, M. A. Nagy, E. A. Pollina, A. M. M. Sousa, T. Shin, C. J. Kenny, J. L. Scotellaro, B. M. Debo, D. M. Gonzalez, L. M. Rento, R. C. Yeh, J. H. T. Song, M. Beaudin, J. Fan, P. V. Kharchenko, N. Sestan, M. E. Greenberg, C. A. Walsh, Rewiring of human neurodevelopmental gene regulatory programs by human accelerated regions. *Neuron* **109**, 3239–3251.e7 (2021).

64. A. Pal, M. A. Noble, M. Morales, R. Pal, M. Baumgartner, J. W. Yang, K. M. Yim, S. Uebbing, J. P. Noonan, Resolving the three-dimensional interactome of human accelerated regions during human and chimpanzee neurodevelopment. *Cell* **188**, 1504–1523.e27 (2025).

65. J. Tchieu, E. L. Calder, S. R. Guttikonda, E. M. Gutzwiller, K. A. Aromolaran, J. A. Steinbeck, P. A. Goldstein, L. Studer, NFIA is a gliogenic switch enabling rapid derivation of functional human astrocytes from pluripotent stem cells. *Nat. Biotechnol.* **37**, 267–275 (2019).

66. T. Wang, K. Hoekzema, D. Vecchio, H. Wu, A. Sulovari, B. P. Coe, M. A. Gillentine, A. B. Wilfert, L. A. Perez-Jurado, M. Kvarnung, Y. Sleyp, R. K. Earl, J. A. Rosenfeld, M. R.

Geisheker, L. Han, B. Du, C. Barnett, E. Thompson, M. Shaw, R. Carroll, K. Friend, R. Catford, E. E. Palmer, X. Zou, J. Ou, H. Li, H. Guo, J. Gerdts, E. Avola, G. Calabrese, M. Elia, D. Greco, A. Lindstrand, A. Nordgren, B. M. Anderlid, G. Vandeweyer, A. Van Dijck, N. Van der Aa, B. McKenna, M. Hancarova, S. Bendova, M. Havlovicova, G. Malerba, B. D. Bernardina, P. Muglia, A. van Haeringen, M. J. V. Hoffer, B. Franke, G. Cappuccio, M. Delatycki, P. J. Lockhart, M. A. Manning, P. Liu, I. E. Scheffer, N. Brunetti-Pierri, N. Rommelse, D. G. Amaral, G. W. E. Santen, E. Trabetti, Z. Sedláček, J. J. Michaelson, K. Pierce, E. Courchesne, R. F. Kooy, J. Acampado, A. J. Ace, A. Amatya, I. Astrovskaya, A. Bashar, E. Brooks, M. E. Butler, L. A. Cartner, W. Chin, W. K. Chung, A. M. Daniels, P. Feliciano, C. Fleisch, S. Ganesan, W. Jensen, A. E. Lash, R. Marini, V. J. Myers, E. O'Connor, C. Rigby, B. E. Robertson, N. Shah, S. Shah, E. Singer, L. A. G. Snyder, A. N. Stephens, J. Tjernagel, B. M. Vernoia, N. Volfovsky, L. C. White, A. Hsieh, Y. Shen, X. Zhou, T. N. Turner, E. Bahl, T. R. Thomas, L. Brueggeman, T. Koomar, R. J. Michael, B. J. O'Roak, R. A. Barnard, R. A. Gibbs, D. Muzny, A. Sabo, K. L. B. Ahmed, E. E. Eichler, M. Siegel, L. Abbeduto, D. G. Amaral, B. A. Hilscher, D. Li, K. Smith, S. Thompson, C. Albright, E. M. Butter, S. Eldred, N. Hanna, M. Jones, D. L. Coury, J. Scherr, T. Pifher, E. Roby, B. Dennis, L. Higgins, M. Brown, M. Alessandri, A. Gutierrez, M. N. Hale, L. M. Herbert, H. L. Schneider, G. David, R. D. Annett, D. E. Sarver, I. Arriaga, A. Camba, A. C. Gulsrud, M. Haley, J. T. McCracken, S. Sandhu, M. Tafolla, W. S. Yang, L. A. Carpenter, C. C. Bradley, F. Gwynette, P. Manning, R. Shaffer, C. Thomas, R. A. Bernier, E. A. Fox, J. A. Gerdts, M. Pepper, T. Ho, D. Cho, J. Piven, H. Lechniak, L. V. Soorya, R. Gordon, A. Wainer, L. Yeh, C. Ochoa-Lubinoff, N. Russo, E. Berry-Kravis, S. Booker, C. A. Erickson, L. M. Prock, K. G. Pawlowski, E. T. Matthews, S. J. Brewster, M. A. Hojlo, E. Abada, E. Lamarche, T. Wang, S. C. Murali, W. T. Harvey, H. E. Kaplan, K. L. Pierce, L. DeMarco, S. Horner, J. Pandey, S. Plate, M. Sahin, K. D. Riley, E. Carmody, J. Constantini, A. Esler, A. Fatemi, H. Hutter, R. J. Landa, A. P. McKenzie, J. Neely, V. Singh, B. Van Metre, E. L. Wodka, E. J. Fombonne, L. Y. Huang-Storms, L. D. Pacheco, S. A. Mastel, L. A. Coppola, S. Francis, A. Jarrett, S. Jacob, N. Lillie, J. Gunderson, D. Istephanous, L. Simon, O. Wasserberg, A. L. Rachubinski, C. R. Rosenberg, S. M. Kanne, A. D. Shocklee, N. Takahashi, S. L. Bridwell, R. L. Klimczac, M. A. Mahurin, H. E. Cotrell, C. A. Grant, S. G. Hunter, C. L. Martin, C. M. Taylor, L. K. Walsh, K. A. Dent, A. Mason, A. Sziklay, C. J. Smith, M. Nordenskjöld, C. Romano, H. Peeters, R. A. Bernier, J. Gecz, K. Xia, Large-scale

targeted sequencing identifies risk genes for neurodevelopmental disorders. *Nat. Commun.* **11**, 4932 (2020).

67. Y. Jiang, M. You, S. Li, Y. Xu, Y. Wang, Perinatal exposure to nonylphenol delayed myelination in offspring cerebellum. *Biochem. Pharmacol.* **178**, 114120 (2020).

68. T. C. Südhof, Neuroligins and neurexins link synaptic function to cognitive disease. *Nature* **455**, 903–911 (2008).

69. R. N. Doan, B.-I. Bae, B. Cubelos, C. Chang, A. A. Hossain, S. Al-Saad, N. M. Mukaddes, O. Oner, M. Al-Saffar, S. Balkhy, G. G. Gascon, Homozygosity Mapping Consortium for Autism, M. Nieto, C. A. Walsh, Mutations in human accelerated regions disrupt cognition and social behavior. *Cell* **167**, 341–354.e12 (2016).

70. A. Kawamura, Y. Katayama, M. Nishiyama, H. Shoji, K. Tokuoka, Y. Ueta, M. Miyata, T. Isa, T. Miyakawa, A. Hayashi-Takagi, K. I. Nakayama, Oligodendrocyte dysfunction due to Chd8 mutation gives rise to behavioral deficits in mice. *Hum. Mol. Genet.* **29**, 1274–1291 (2020).

71. D. Velmeshev, L. Schirmer, D. Jung, M. Haeussler, Y. Perez, S. Mayer, A. Bhaduri, N. Goyal, D. H. Rowitch, A. R. Kriegstein, Single-cell genomics identifies cell type–specific molecular changes in autism. *Science* **364**, 685–689 (2019).

72. C. E. Finch, S. N. Austad, Commentary: Is Alzheimer's disease uniquely human? *Neurobiol. Aging* **36**, 553–555 (2015).

73. J. P. Bolam, E. K. Pissadaki, Living on the edge with too many mouths to feed: Why dopamine neurons die. *Mov. Disord.* **27**, 1478–1483 (2012).

74. M. P. Mattson, T. Magnus, Ageing and neuronal vulnerability. *Nat. Rev. Neurosci.* **7**, 278–294 (2006).

75. W. Yin, A. Reichenberg, M. Schnaider Beeri, S. Z. Levine, J. F. Ludvigsson, M. Figee, S. Sandin, Risk of Parkinson disease in individuals with autism spectrum disorder. *JAMA Neurol.* **82**, 687–695 (2025).

76. M. Naddaf, "Autistic people three times more likely to develop Parkinson's-like symptoms," *Nature*, 28 May 2024; https://doi.org/10.1038/D41586-024-01572-W.

77. T. Stuart, A. Butler, P. Hoffman, C. Hafemeister, E. Papalexi, W. M. Mauck, Y. Hao, M. Stoeckius, P. Smibert, R. Satija, Comprehensive integration of single-cell data. *Cell* **177**, 1888–1902.e21 (2019).

78. B. Muzellec, M. Teleńczuk, V. Cabeli, M. Andreux, PyDESeq2: A python package for bulk RNA-seq differential expression analysis. *Bioinformatics* **39**, btad547 (2023).

79. G. E. Hoffman, D. Lee, J. Bendl, P. Fnu, A. Hong, C. Casey, M. Alvia, Z. Shao, S. Argyriou, K. Therrien, S. Venkatesh, G. Voloudakis, V. Haroutunian, J. F. Fullard, P. Roussos, Efficient differential expression analysis of large-scale single cell transcriptomics data using dreamlet. bioRxiv 2023.03.17.533005 [Preprint] (2024). https://doi.org/10.1101/2023.03.17.533005.

80. N. J. Schurch, P. Schofield, M. Gierliński, C. Cole, A. Sherstnev, V. Singh, N. Wrobel, K. Gharbi, G. G. Simpson, T. Owen-Hughes, M. Blaxter, G. J. Barton, How many biological replicates are needed in an RNA-seq experiment and which differential expression tool should you use? *RNA* **22**, 839–851 (2016).

81. S. Das, A. Rai, M. L. Merchant, M. C. Cave, S. N. Rai, A comprehensive survey of statistical approaches for differential expression analysis in single-cell rna sequencing studies. *Genes (Basel)* **12**, 1947 (2021).

82. S. I. Ramos, Z. M. Mussa, E. N. Falk, B. Pai, B. Giotti, K. Allette, P. Cai, F. Dekio, R. Sebra, K. G. Beaumont, A. M. Tsankov, N. M. Tsankova, An atlas of late prenatal human neurodevelopment resolved by single-nucleus transcriptomics. *Nat. Commun.* **13**, 7671 (2022).
